# Mitigating algorithmic unfairness arising from forgetfulness of medical records in clinical artificial intelligence

**DOI:** 10.1038/s41467-026-72601-7

**Published:** 2026-05-04

**Authors:** Yixuan Chen, Anshul Thakur, Andrew A. S. Soltan, Yifei Shen, Dongsheng Li, Mingzhi Dong, Li Shang, David A. Clifton, Yujiang Wang

**Affiliations:** 1 Oxford Suzhou Centre for Advanced Research, University of Oxford, Suzhou, China; 2 Department of Engineering Science, University of Oxford, Oxford, UK; 3 Oxford University Hospitals NHS Foundation Trust, Oxford, UK; 4 Department of Oncology, University of Oxford, Oxford, UK; 5 Microsoft Research Asia, Shanghai, China; 6 Department of Computer Science, University of Bath, Bath, UK; 7 College of Computer Science and Artificial Intelligence, Fudan University, Shanghai, China; 8 Intelligent Medicine Institute, Fudan University, Shanghai, China

**Keywords:** Medical ethics, Diagnosis, Viral infection

## Abstract

Protecting patients’ right to be forgotten (RtbF) in the digital era underscores algorithms to delete health records from deployed artificial intelligence (AI) models upon requests, a paradigm also known as machine unlearning. Such forgetfulness, however, will alter AI models’ views of subpopulations and could yield discriminating clinical decisions that undermine healthcare equality and endanger patient trust, a common phenomenon confirmed by our thorough investigations. This observation reveals an ethical dilemma in clinical AI: protecting the RtbF of certain patients will compromise the delivery of equitable decisions and, thus, is against the AI fairness principle, necessitating a safeguarding approach. To this end, we propose a fair unlearning strategy to effectively remove medical records from trained models while mitigating decision biases to improve algorithmic equality. This strategy avoids negative interventions between potentially conflicting objectives by enforcing their gradient orthogonality. We perform extensive evaluations on real-world, multi-hospital datasets of rapid COVID-19 screening, in-hospital mortality and shock predictions. Compared with state-of-the-art approaches, our method can teach models to more effectively forget patient records with better predictive performance while, more importantly, mitigating demographic unfairness across subpopulations of ethnicities and sites (hospitals), accommodating the first generalised solution for the dilemma of RtbF and fairness.

## Abstract (web summary)

Applying the right to be forgotten to electronic health records that have been used to train artificial intelligence models could compromise model accuracy and fairness. Here, the authors develop a machine unlearning model that aims to remove data whilst preserving algorithmic fairness across subgroups.

## Introduction

We live in a society that admires learning and remembering but commonly associates forgetting with failures and frustrations. However, judicious forgetting is a fundamental value for humans^1^. As realised by scholars, it is an indispensable component in the design of our memory^1^, a condition for forgiveness^1^ and wound healing^2^, and plays a critical role in our decision-making by permitting abstractions of daily experiences^3^. In medical research, the capability of forgetting or being forgotten is a prerequisite for individual privacy protections and is, therefore, pivotal to winning patient trust, which will benefit all stakeholders in the healthcare system. The virtue of forgetting has been recently legitimised as the Right to be Forgotten (RtbF), previously known as “Right to Oblivion” in some countries, under Article 17 of the General Data Protection Regulation (GDPR)^4^ applied across the European Union and the United Kingdom. RtbF ensures that individuals have the right to demand their personal data be deleted, and data controllers are mandated to erase it once they receive the request.

Notably, RtbF enforces a retro-active erasure^5^. Personal data, such as health records, should be removed from all available sources where the data resides, including the artificial intelligence (AI) algorithms and models built upon. This is due to the fact that AI models, once trained on medical data, could remember sensitive personal information that could be extracted by attackers and threaten individual privacy^6–8^. From a legal perspective, the capability of performing forgetfulness in AI models is necessary for giving control over personal data back to patients who are usually unaware of the value of their health records. With the growing penetration of AI into medical research and clinical routine tasks^9–11^, designing, interpreting, and reflecting on the methods to achieve RtbF in clinical AI studies are essential for constructing clinicians’ trustworthiness among patients and securing diverse datasets vital to fostering AI-based innovations. However, challenges prevail.

Unlike the straightforwardness of deleting medical documents from a digital database system, data removal in intelligent algorithms and models is a challenging issue with potential societal consequences. The most naive way is to retrain an AI model on a dataset that excludes the withdrawn patient data. However, it is also an impractical method in healthcare. The worldwide application of clinical AI systems also indicates a high frequency of data revocations, e.g., large techs like Google could receive millions of data deletion requests per month^12^. Therefore, it is resourcefully and timely impossible to entirely retrain an AI model every time such a request is made, especially when the model has been deployed to clinics with limited computational hardware. The infeasibility of performing retraining for RtbF underscores the algorithms to directly and efficiently remove medical records from deployed AI models, a paradigm termed *machine unlearning*^13,14^ in the AI community.

Just like how we underrate the virtue of forgetting in our daily lives, the cruciality of forgetfulness in machine intelligence, driven by machine unlearning algorithms, is also undervalued. Studies of removing records from AIs in healthcare and medical fields substantially lag behind their counterparts with reversed objectives, i.e., machine learning methods for memorising knowledge. The ignorance of the importance of forgetfulness has resulted in two notable challenges that significantly hurdle the advancement of clinical AI and the winning of patient trust. From an algorithmic view, machine unlearning approaches commonly suffer from the *catastrophic forgetting* issue, i.e., removing specific records will lead AI models to forget other data points with similar patterns. Thus, the overall predictive performance of AIs could drop sharply after forgetting, impairing their clinical utilities, generalisations, and abilities to deliver algorithmic insights. More importantly, the potential societal impacts of AI forgetfulness are mostly uncharted, even though those unlearning methods will alter the views, behaviours, and decision-making of intelligent models that could affect the diagnosis, care, and life qualities of an expansive range of patients.

This manuscript investigates and identifies a crucial yet overlooked societal influence of AI forgetfulness: clinical AI models will make more discriminatory decisions among subpopulations after unlearning. Specifically, we thoroughly evaluate prevalent machine unlearning algorithms on multiple real-world clinical tasks, and we have confirmed that those unlearning methods will yield AI models with more imbalanced performance across sensitive patient subgroups, including ethnicities and hospital sites (see Section “Results” for details).

The introduced algorithmic biases from data removals could, therefore, lead to inaccurate, life-changing clinical decisions among minority groups, undermine the equality of patient-care delivery, and accumulatively exacerbate existing healthcare inequities that could worsen disparate health outcomes among subpopulations. In addition, a biased AI system could never win the trust of underserved communities, putting the trustworthiness of clinicians and researchers at risk and endangering the cultivation of AI innovations.

More profoundly, we interpret our observations as the collision of two ethical and legal pillars for clinical AI research: RtbF and AI fairness. The latter regulation is legitimated under Article 22 of GDPR (and also Annex A), which states that AI systems are obligated to address the risks of bias and discrimination to secure the fair treatment and non-discrimination of individuals. However, implementing RtbF using current machine unlearning methods will amplify the inequities of medical AI systems and, consequently, violate the fairness regulation. In other words, current technology cannot simultaneously satisfy the two key regulations in AIs, while, legally speaking, protecting the right to be forgotten of individuals should not compromise AI models’ decision fairness and the right to non-discrimination of other patients. Those observations unveil a dilemma between implementing RtbF and fairness regulations in clinical AI studies. As more and more intelligent algorithms and models are being approved by the Food and Drug Administration (FDA) as AI-based software as a medical device (AI-SaMD)^15,16^, devising a practical and effective solution to the RtbF-fairness dilemma is, therefore, a missing but crucial puzzle to the picture of safeguarding the legal rights of all patients in clinical routine tasks deeply penetrated by AI-based services and products.

To this end, we present such a solution by proposing a novel and generalised machine unlearning framework. Standing in the heart is a *fair forgetting* mechanism, which aims to minimise the decision discrimination stratified across patient subgroups while removing health records from deep neural networks (DNNs). The potentially negative, undesirable interventions between potentially contrastive objectives, e.g., mitigating fairness and removing data, are avoided by enforcing the orthogonalities of their gradients in DNNs’ latent space. Moreover, the mechanism prevents catastrophic forgetting by enforcing the orthogonalities between the gradients from unlearning records and those from learning the rest, minimising the negative impacts of forgetting specific data on the already-learned knowledge of other patients. We unify all orthogonal conditions into a concise, straightforward objective for better computational efficiency. Specifically, we apply the Gram-Schmidt orthogonalisation to project the forgetting gradient onto a subspace orthogonal to the utility and fairness gradients. Generally, our design is a stone that kills two birds: the algorithmic unfairness of forgetting medical records can be adequately mitigated, and the catastrophic forgetting issue can be effectively addressed.

We adopt multiple prevalent machine unlearning methods as the baselines. We evaluate baselines and our method on four real-world, multiple-site datasets, CURIAL^17,18^, CURIAL-Combined (a variant of CURIAL), eICU Collaborative Research Database (eICU-CRD or eICU)^19^, and Medical Information Mart for Intensive Care IV (MIMIC-IV)^20^, across various clinical tasks, including COVID-19 screening from tabular electronic health records (EHRs), in-hospital mortality and shock predictions from time-series EHRs. We report the diagnostic performance and the privacy attack results of unlearned DNNs to evaluate the effectiveness of unlearning methods. We evaluate the decision biases across patient subgroups of ethnicities and hospitals to measure the algorithmic fairness after unlearning. It is shown that our fair unlearning framework has attained the best forgetfulness performance and, more favourably, it significantly mitigates the decision unfairness prominent in baselines. Our unlearning framework, with the first fair forgetting mechanism for healthcare, can be applied to different neural network architectures, EHR structures, clinical scenarios, etc., and, therefore, serves as the first generalised solution to the RtbF-fairness dilemma in clinical AI.

## Results

### Datasets

We employ the following datasets to evaluate the clinical performance and unfairness of different machine unlearning algorithms:

- The CURIAL dataset^17,18^ consists of anonymised electronic health record (EHR) data from emergency departments across four UK National Health Service (NHS) Trusts: Oxford University Hospitals (OUH), University Hospitals Birmingham (UHB), Bedfordshire Hospitals (BH), and Portsmouth Hospitals University (PUH). We adopt a pre-processed version of CURIAL^21^ that includes 215,652 patients’ clinical data (blood tests, blood gas, and vital signs) routinely collected within the first hour of arrival to the emergency department. The clinical task is to rapidly identify the COVID-19 status of patients using the medical records from emergency admissions.
  We follow the timeline in the previous work^21^ to separate data into two distinct pandemic waves, and we train models exclusively on patient presentations from the OUH trust, including positive samples from the first COVID-19 pandemic wave (wave 1) in the UK and controls from the pre-pandemic stage. Prospective and external evaluations are conducted on the second pandemic wave (wave 2) data from OUH, PUH and BH, respectively, and wave 1 data from UHB. Clinical records are curated and pre-processed as tabular data following prior works^22–24^, and we train a DNN of two fully-connected layers, i.e., a multilayer perceptron (MLP), to predict the infection of COVID-19, assessing the ethnicity unfairness of machine unlearning methods.
- CURIAL-Combined is a dataset that combines the data from all four trusts in CURIAL with a cohort of 215,652 patients. We split the dataset into train and test sets by an 80:20 ratio. We train a two-layer MLP to determine COVID-19 status and examine the geographic inequalities (patients attending different hospital sites) after forgetfulness.
- The eICU dataset^19^ is a large-scale, publicly available, multi-centre clinical research database. We pre-process EHRs into time-series data following the prior work^25^, and we perform evaluations on two clinical tasks: 1). in-hospital mortality predictions using 30,529 EHRs from the first 48 hours of ICU stay, and 2). Shock predictions within the next 4 hours using 48,288 ICU stays. The dataset is split into an 80:20 ratio for training and testing. Across both tasks, we train a long short-term memory (LSTM) network for time-series data and evaluate the ethnicity inequalities of different unlearning algorithms.
- The MIMIC-IV dataset^20^ contains de-identified EHRs from patients admitted to the intensive care units at Beth Israel Deaconess Medical Centre in Boston, Massachusetts, between 2008 and 2019. We generally follow the existing protocol^26^ to curate and pre-process 54,831 EHRs collected during the first 24 h after ICU admission into time-series data. The clinical task is to predict in-hospital mortality. We split all records into an 80:20 ratio for training and testing, and we train an LSTM network to evaluate the ethnicity unfairness of various unlearning methods.

Table 1 summarises the key cohort characteristics across all the datasets. It can be observed that the demographic information, such as ethnicities and attending hospitals, demonstrates highly skewed distributions. Therefore, unlearning models trained on such biased data will inevitably exacerbate the unfairness of their predictions and call for an effective mitigating strategy. We thoroughly outline the data splits, pre-processing and clinical variables in Supplementary Note 1 and specify the network architectures in Supplementary Table 5.

**Table 1:** Summary of population characteristics of CURIAL, CURIAL-Combined, eICU, and MIMIC-IV datasets

CURIAL						
	OUH (pre-pandemic)	OUH (wave 1)	OUH (wave 2)	PUH (wave 2)	UHB (wave 1)	BH (wave 2)
Cohort	1/12/2018–30/11/2019	13/3/2020–29/10/2020	01/11/2020–06/03/2021	01/11/2020–01/03/2021	18/05/2019–08/05/2020	01/01/2021–31/03/2021
*n*, total	85,607	855	18,499	13,592	95,236	1863
*n*, positive	0	855	1908 (10.3)	1488 (10.9)	790 (0.8)	210 (11.3)
Ethnicity (%)						
White	69,234 (80.9)	575 (67.3)	14,035 (75.9)	10,205 (75.1)	59,683 (62.7)	1585 (85.1)
Unknown	10,923 (12.8)	161 (18.9)	3340 (18.0)	3086 (22.7)	11,573 (12.2)	*
South Asian	2007 (2.3)	38 (4.4)	369 (2.0)	65 (0.5)	13,883 (14.6)	123 (6.6)
Other	1432 (1.7)	37 (4.3)	347 (1.9)	96 (0.7)	3011 (3.2)	46 (2.5)
Black	1016 (1.2)	29 (3.4)	238 (1.3)	72 (0.5)	4669 (4.9)	76 (4.1)
Mixed	792 (0.9)	13 (1.5)	126 (0.7)	53 (0.4)	1944 (2.0)	27 (1.4)
Chinese	203 (0.2)	*	44 (0.2)	15 (0.1)	473 (0.5)	*
Age (%)						
18–39	21,027 (24.6)	90 (10.5)	3030 (16.4)	2558 (18.8)	29,652 (31.1)	389 (21.3)
40–64	25,724 (30.0)	295 (34.5)	5545 (30)	3354 (24.7)	32,031 (33.6)	619 (33.8)
> 65	38,856 (45.4)	470 (55.0)	9924 (53.6)	7680 (56.5)	33,553 (35.2)	822 (44.9)

CURIAL-Combined					
	Hospital (%)				Combined
	OUH	PUH	UHB	BH	
*n*, total	104,961 (48.7)	13,592 (6.3)	95,236 (44.2)	1863 (0.9)	215,652
*n*, positive	2763 (2.6)	1488 (10.9)	790 (0.8)	210 (11.3)	5251

eICU							
Task	*n*, total	Ethnicity(%)					
		Caucasian	Afr. Am.	Hispanic	Asian	Nat. Am.	Other/Unk.
Mortality	30,529	23,655 (77.5)	3402 (11.1)	1111 (3.6)	492 (1.6)	–	1869 (6.1)
Shock	48,288	36,803 (76.2)	5562 (11.5)	2254 (4.7)	725 (1.5)	262 (0.5)	2682 (5.6)

MIMIC-IV							
Task	*n*, total	Ethnicity(%)					
		White	Unknown	Black	Other	Hispanic	Asian
Mortality	54,831	37,118 (67.7)	6077 (11.1)	5722 (10.4)	2320 (4.2)	2014 (3.7)	1580 (2.9)

Cohort characteristics of ethnicities and ages across four UK NHS trusts. Data is divided into two COVID-19 pandemic waves. Models are trained on positive cases of OUH wave 1 data and OUH pre-pandemic controls. Prospective and external evaluations are performed on the wave 2 data of OUH, PUH, PH, and wave 1 of UHB. * We merged cases from these subgroups into the “Other” cohort for statistical disclosure controls. Age groups (18–39, 40–64,  > 65) are reported for adult patients with available age information. Patients younger than 18 years in the BH cohort (*n* = 33) were excluded from all corresponding analyses.

Cohort characteristics across four NHS trusts.

Cohort characteristics of ethnicities for the mortality and shock prediction tasks in eICU. Abbreviations: *Afr. Am* African American; *Nat. Am* Native American; *Other/Unk* Other/Unknown.

Cohort characteristics of ethnicities for the mortality prediction task in MIMIC-IV.

### Unlearning medical records

Across four datasets, we first train a DNN on the full training set to represent the well-learned, ready-made AI models deployed to clinical scenarios. This trained network is denoted as the *original model* throughout the manuscript. To implement RtbF, we need to remove specific data records in the training set from the original model, i.e., unlearning those records. We simulate the different amounts of medical records to be forgotten by randomly selecting varying ratios of the training data as the forgetting sets. Ideally and traditionally, after unlearning, the model should: (1). possess no knowledge of the forgetting set, or more concisely, achieve *unlearning effectiveness*, and (2). preserve the predictive performance on the remaining training data (the remaining set) and prospective cohorts, i.e., retain *clinical utilities*. Notably, this manuscript underlines a previously overlooked but clinically and societally crucial criterion: (3). Unlearning should not lead to more discriminatory predictions across sensitive subgroups, i.e., preserved or improved *algorithmic fairness*.

As such, we propose a fair unlearning (FU) method that enforces orthogonalities in the gradient space to simultaneously address the catastrophic forgetting issue and mitigate algorithmic inequalities from machine unlearning. We thoroughly describe our FU strategy in Section “Methods”. On each dataset, we randomly sample 1%, 5%, and 10% of training records as the forgetting sets of varying sizes, and we apply prevalent machine unlearning algorithms and our FU method to the original model to remove each of those forgetting sets. Three facets of the unlearned models are measured: unlearning effectiveness, clinical utilities (diagnostic performance), and algorithmic unfairness. We also investigate the fine-grained behaviours of unlearned models to understand the underlying mechanism of various unlearning algorithms and to illustrate the clinical advantages of the proposed FU method.

### Baselines and evaluation metrics

Specifically, we compare the proposed FU with the following machine-unlearning baselines:

- Gradient Ascent (GA)^27^ is probably the most classic and extensively used approach for unlearning data by ascending the gradients of the forgetting set by a few steps.
- Certified Removal (CR)^28^ is a one-step gradient ascent method using the idea of Newton update.
- Catastrophic Forgetting (CF)^29^ is a GA variant that freezes several layers of DNN and conducts unlearning on the rest of the network.
- Scalable Remembering and Unlearning unBound (SCRUB)^30^ involves the idea of knowledge distillation to perform machine unlearning.
- Orthogonalisation Gradient (ORTHO)^31^ performs per-sample gradient orthogonalisation by projecting forget gradients onto the orthogonal subspace of retain gradients.
- We additionally report the performance of the model fully retrained on the remaining set (the training set excluding the records to be forgotten), denoted as “Retrained”. Retraining the model is a routine for achieving genuine forgetting; however, the high computational cost makes it an infeasible solution for clinical deployments.

The three criteria for unlearning are measured by the following metrics:

- Clinical utility, or diagnostic performance, is evaluated by the unlearned model’s average area under the ROC curve (AUROC) on test sets. Higher AUROC values indicate better performance.
- Unlearning effectiveness is quantified by two types of privacy attacks, membership inference attack^32^ (MIA) and model inversion^33^ (MI), on those records in the forgetting set. The result of MIA is an AUROC score, denoted as MIA-AUROC. 0.5 implies a perfect forgetting, while overly high or low MIA-AUROC values indicate risks of information leaks of the forgetting set, suggesting inadequate forgetfulness.
  We follow the prior MI attack implementation^33^ and adopt K-Nearest Neighbour Distance^34^, denoted as MI-KnnDist, to measure the similarity between each reconstructed patient record and its K nearest neighbours in the forgetting set (we set K to 10 across all experiments). A lower MI-KnnDist indicates that the reconstructed records better resemble the records to be forgotten, suggesting a higher privacy-leak risk. In contrast, a higher MI-KnnDist value indicates more thorough unlearning and stronger privacy protections.
- Algorithmic fairness is measured by well-known fairness metrics, equalised odds (EO) and demographic parity (DP). The former is computed as the standard deviation (SD) of the true positive (TP) and false positive (FP) rates across patient subgroups, denoted EO-TP and EO-FP, respectively. DP is defined as the positive prediction rates across demographic groups, which complements EO by concentrating on selection-rate disparities independent of ground-truth labels. Lower EO and DP values indicate better algorithmic equality and vice versa. We provide the formal definition of EO and DP in Section “Methods”.

We repeat each unlearning experiment with five independent runs and report the average performance with SDs to ensure evaluation robustness. A detailed description of the experimental settings is provided in Section “Methods”.

### Our FU effectively mitigates algorithmic unfairness after forgetting medical records

We first present the results of algorithmic fairness after unlearning forgetting sets of varying sizes from the original model on CURIAL, CURIAL-Combined, eICU, and MIMIC-IV datasets in Tables 2 and 3. From those tables, we can discover that all baseline unlearning methods will commonly yield AI models with exacerbated inequalities compared to the original ones across various clinical datasets, diagnostic tasks, and protected subgroups like ethnicities and hospital sites. For example, at the 5% forgetting ratio, baseline methods increase algorithmic unfairness by 1–109% on CURIAL, 4–115% on CURIAL-Combined, 2–88% on eICU, and 4–71% on MIMIC-IV, respectively. Generally, the more records are forgotten, the more discriminatory predictions will be made. Those observations reveal that if they were clinically employed, those unlearning algorithms could potentially reinforce existing healthcare disparities and inequitable care that are already affecting socioeconomically disadvantaged populations.

**Table 2:** Ethnicity-specific algorithmic unfairness of various methods on the four UK NHS trusts of CURIAL

	EO-TP *↓* (SD)				EO-FP *↓* (SD)				DP *↓* (SD)			
Forg. Ratio	0%	1%	5%	10%	0%	1%	5%	10%	0%	1%	5%	10%
OUH (wave 2)												
Original	0.060	–	–	–	0.052	–	-	–	0.089	–	–	–
Retrained	–	0.048(0.006)	0.055(0.004)	0.054(0.004)	–	0.043(0.007)	0.036(0.004)	0.040(0.006)	–	0.079(0.006)	0.075(0.002)	0.080(0.003)
Unlearning Methods												
FU (Ours)	–	**0.057**(0.001)	**0.049**(0.001)	**0.046**(0.001)	–	**0.049**(0.000)	**0.045**(0.001)	**0.044**(0.000)	–	0.082(0.002)	0.079(0.002)	**0.072**(0.001)
GA	–	0.060(0.002)	0.063(0.009)	0.066(0.008)	–	0.053(0.002)	0.064(0.003)	0.064(0.003)	–	0.132(0.007)	0.090(0.006)	0.085(0.006)
CR	–	0.057(0.004)	0.064(0.007)	0.066(0.006)	–	0.050(0.005)	0.058(0.011)	0.066(0.007)	–	0.084(0.005)	0.087(0.009)	0.092(0.007)
CF	–	0.062(0.001)	0.075(0.009)	0.076(0.007)	–	0.052(0.009)	0.068(0.009)	0.076(0.011)	–	0.092(0.006)	0.087(0.007)	0.080(0.013)
SCRUB	–	0.061(0.006)	0.062(0.003)	0.070(0.013)	–	0.052(0.005)	0.053(0.004)	0.054(0.010)	–	**0.079**(0.004)	**0.073**(0.004)	0.073(0.002)
ORTHO	–	0.064(0.008)	0.068(0.006)	0.063(0.008)	–	0.056(0.017)	0.053(0.013)	0.058(0.010)	–	0.098(0.026)	0.078(0.020)	0.076(0.009)
PUH (wave 2)												
Original	0.087	–	–	–	0.046	–	-	–	0.042	–	–	–
Retrained	–	0.078(0.008)	0.099(0.016)	0.112(0.005)	–	0.047(0.007)	0.042(0.005)	0.040(0.007)	–	0.060(0.014)	0.063(0.016)	0.053(0.012)
Unlearning Methods												
FU (Ours)	–	**0.077**(0.008)	**0.075**(0.008)	**0.055**(0.013)	–	**0.046**(0.002)	**0.039**(0.003)	**0.040**(0.004)	–	**0.036**(0.002)	**0.036**(0.006)	**0.040**(0.006)
GA	–	0.089(0.005)	0.118(0.016)	0.148(0.018)	–	0.047(0.002)	0.054(0.008)	0.049(0.003)	–	0.048(0.011)	0.049(0.012)	0.050(0.003)
CR	–	0.084(0.002)	0.096(0.012)	0.106(0.014)	–	0.046(0.004)	0.052(0.009)	0.058(0.009)	–	0.044(0.009)	0.047(0.006)	0.054(0.014)
CF	–	0.087(0.005)	0.101(0.009)	0.090(0.005)	–	0.052(0.013)	0.062(0.020)	0.068(0.019)	–	0.051(0.017)	0.042(0.005)	0.054(0.005)
SCRUB	–	0.083(0.004)	0.122(0.018)	0.131(0.016)	–	0.046(0.006)	0.049(0.005)	0.051(0.002)	–	0.046(0.015)	0.046(0.005)	0.051(0.013)
ORTHO	–	0.086(0.010)	0.085(0.005)	0.075(0.011)	–	0.046(0.002)	0.049(0.010)	0.058(0.015)	–	0.048(0.014)	0.045(0.005)	0.054(0.010)
UHB (wave 1)												
Original	0.092	–	–	–	0.021	–	-	–	0.020	–	–	–
Retrained	–	0.093(0.002)	0.070(0.014)	0.095(0.021)	–	0.026(0.003)	0.028(0.003)	0.026(0.002)	–	0.019(0.002)	0.022(0.003)	0.023(0.004)
Unlearning Methods												
FU (Ours)	–	**0.090**(0.001)	0.093(0.005)	**0.087**(0.016)	–	**0.020**(0.006)	**0.021**(0.002)	**0.021**(0.002)	–	**0.024**(0.001)	**0.024**(0.005)	**0.025**(0.009)
GA	–	0.090(0.005)	0.092(0.009)	0.113(0.021)	–	0.023(0.003)	0.034(0.009)	0.044(0.008)	–	0.024(0.003)	0.027(0.006)	0.037(0.011)
CR	–	0.092(0.005)	0.125(0.009)	0.109(0.009)	–	0.023(0.003)	0.044(0.014)	0.036(0.012)	–	0.025(0.002)	0.027(0.001)	0.029(0.002)
CF	–	0.090(0.009)	0.125(0.018)	0.125(0.011)	–	0.023(0.003)	0.044(0.012)	0.045(0.012)	–	0.028(0.005)	0.036(0.006)	0.035(0.004)
SCRUB	–	0.095(0.006)	**0.090**(0.009)	0.111(0.009)	–	0.024(0.005)	0.030(0.004)	0.033(0.004)	–	0.026(0.002)	0.026(0.002)	0.035(0.004)
ORTHO	–	0.092(0.009)	0.092(0.006)	0.087(0.007)	–	0.024(0.004)	0.023(0.003)	0.025(0.002)	–	0.025(0.005)	0.024(0.003)	0.026(0.002)
BH (wave 2)												
Original	0.148	–	–	–	0.151	–	-	–	0.190	–	–	–
Retrained	–	0.137(0.016)	0.116(0.004)	0.129(0.018)	–	0.150(0.025)	0.142(0.012)	0.117(0.029)	–	0.151(0.041)	0.166(0.027)	0.180(0.023)
Unlearning Methods												
FU (Ours)	–	**0.140**(0.009)	**0.098**(0.017)	**0.102**(0.020)	–	0.151(0.001)	**0.140**(0.005)	**0.124**(0.011)	–	**0.186**(0.001)	0.180(0.004)	**0.182**(0.000)
GA	–	0.145(0.008)	0.149(0.003)	0.152(0.002)	–	0.151(0.002)	0.146(0.004)	0.146(0.016)	–	0.194(0.011)	0.166(0.037)	0.189(0.020)
CR	–	0.149(0.001)	0.144(0.005)	0.236(0.031)	–	0.152(0.006)	0.149(0.007)	0.151(0.008)	–	0.195(0.021)	0.188(0.012)	0.186(0.069)
CF	–	0.151(0.005)	0.152(0.004)	0.160(0.011)	–	**0.144**(0.012)	0.144(0.006)	0.147(0.011)	–	0.186(0.028)	0.188(0.012)	0.189(0.008)
SCRUB	–	0.144(0.014)	0.136(0.018)	0.154(0.016)	–	0.152(0.005)	0.157(0.019)	0.144(0.017)	–	0.189(0.015)	**0.155**(0.052)	0.183(0.007)
ORTHO	–	0.151(0.003)	0.146(0.006)	0.152(0.004)	–	0.150(0.002)	0.152(0.003)	0.141(0.009)	–	0.188(0.012)	0.186(0.036)	0.183(0.007)

We evaluate three different forgetting set sizes: 1%, 5%, and 10% ("forgetting ratios”) of the training set. “0%” denotes the full training set on which the original model is learned. “Retrained” denotes the same model entirely retrained on the remaining set. Bold values indicate the best-performing result in each metric.

**Table 3:** Site-specific (CURIAL-Combined) and ethnicity-specific (eICU and MIMIC-IV) algorithmic unfairness of various methods on the three datasets

CURIAL-Combined												
	EO-TP *↓* (SD)				EO-FP *↓* (SD)				DP *↓* (SD)			
Forg. Ratio	0%	1%	5%	10%	0%	1%	5%	10%	0%	1%	5%	10%
Original	0.013	–	–	–	0.023	–	–	–	0.058	–	–	–
Retrained	–	0.014(0.006)	0.016(0.006)	0.026(0.003)	–	0.023(0.006)	0.017(0.003)	0.023(0.005)	–	0.069(0.004)	0.060(0.011)	0.059(0.014)
Unlearning Methods												
FU (Ours)	–	**0.010**(0.002)	**0.012**(0.002)	**0.007**(0.002)	–	**0.017**(0.003)	**0.014**(0.001)	**0.010**(0.001)	–	**0.053**(0.003)	**0.049**(0.003)	**0.046**(0.001)
GA	–	0.016(0.002)	0.021(0.007)	0.017(0.010)	–	0.023(0.002)	0.025(0.004)	0.025(0.003)	–	0.061(0.008)	0.083(0.010)	0.052(0.003)
CR	–	0.013(0.005)	0.028(0.009)	0.030(0.007)	–	0.025(0.004)	0.026(0.004)	0.033(0.011)	–	0.066(0.011)	0.066(0.016)	0.061(0.015)
CF	–	0.016(0.005)	0.017(0.005)	0.015(0.002)	–	0.023(0.005)	0.023(0.011)	0.033(0.009)	–	0.061(0.016)	0.064(0.025)	0.062(0.025)
SCRUB	–	0.020(0.007)	0.024(0.008)	0.022(0.008)	–	0.023(0.004)	0.024(0.005)	0.027(0.002)	–	0.073(0.007)	0.071(0.004)	0.069(0.015)
ORTHO	–	0.014(0.002)	0.026(0.007)	0.021(0.003)	–	0.038(0.012)	0.024(0.007)	0.024(0.006)	–	0.071(0.007)	0.083(0.010)	0.086(0.012)

eICU												
	EO-TP *↓* (SD)				EO-FP *↓* (SD)				DP *↓* (SD)			
Forg. Ratio	0%	1%	5%	10%	0%	1%	5%	10%	0%	1%	5%	10%
Mortality Prediction												
Original	0.041	–	–	–	0.014	–	–	–	0.021	–	–	–
Retrained	–	0.038(0.006)	0.041(0.019)	0.043(0.013)	–	0.015(0.002)	0.012(0.002)	0.014(0.004)	–	0.016(0.002)	0.015(0.003)	0.019(0.003)
Unlearning Methods												
FU (Ours)	–	**0.032**(0.001)	**0.039**(0.002)	**0.036**(0.001)	–	**0.013**(0.001)	0.018(0.001)	**0.013**(0.001)	–	**0.019**(0.001)	**0.020**(0.000)	**0.019**(0.001)
GA	–	0.043(0.006)	0.048(0.010)	0.057(0.003)	–	0.018(0.002)	0.019(0.002)	0.026(0.003)	–	0.020(0.004)	0.037(0.005)	0.030(0.003)
CR	–	0.044(0.008)	0.077(0.023)	0.068(0.024)	–	0.013(0.003)	0.024(0.005)	0.028(0.010)	–	0.022(0.006)	0.036(0.021)	0.034(0.009)
CF	–	0.048(0.004)	0.070(0.005)	0.078(0.014)	–	0.014(0.003)	0.020(0.010)	0.019(0.003)	–	0.024(0.006)	0.024(0.004)	0.031(0.006)
SCRUB	–	0.047(0.009)	0.075(0.014)	0.075(0.019)	–	0.019(0.008)	0.023(0.004)	0.020(0.005)	–	0.023(0.008)	0.027(0.007)	0.027(0.004)
ORTHO	–	0.047(0.014)	0.068(0.006)	0.075(0.023)	–	0.017(0.002)	**0.017**(0.006)	0.022(0.003)	–	0.022(0.002)	0.021(0.004)	0.026(0.004)
Shock Prediction												
Original	0.070	–	–	–	0.045	–	–	–	0.052	–	–	–
Retrained	–	0.069(0.012)	0.067(0.018)	0.074(0.013)	–	0.043(0.009)	0.039(0.004)	0.039(0.014)	–	0.051(0.004)	0.049(0.004)	0.054(0.004)
Unlearning Methods												
FU (Ours)	–	**0.058**(0.001)	**0.054**(0.008)	**0.048**(0.001)	–	**0.040**(0.001)	**0.040**(0.003)	**0.035**(0.001)	–	**0.044**(0.001)	**0.038**(0.001)	**0.027**(0.001)
GA	–	0.083(0.008)	0.083(0.003)	0.097(0.020)	–	0.044(0.003)	0.050(0.005)	0.076(0.009)	–	0.047(0.010)	0.047(0.011)	0.077(0.014)
CR	–	0.084(0.004)	0.087(0.010)	0.088(0.009)	–	0.050(0.008)	0.062(0.007)	0.056(0.005)	–	0.079(0.011)	0.062(0.012)	0.059(0.009)
CF	–	0.064(0.016)	0.091(0.012)	0.093(0.007)	–	0.045(0.010)	0.056(0.019)	0.056(0.006)	–	0.055(0.015)	0.048(0.007)	0.044(0.004)
SCRUB	–	0.083(0.016)	0.078(0.013)	0.089(0.017)	–	0.049(0.010)	0.056(0.008)	0.061(0.017)	–	0.045(0.007)	0.049(0.006)	0.044(0.012)
ORTHO	–	0.075(0.011)	0.077(0.009)	0.094(0.037)	–	0.051(0.004)	0.046(0.005)	0.037(0.008)	–	0.053(0.004)	0.049(0.004)	0.041(0.006)

MIMIC-IV												
	EO-TP *↓* (SD)				EO-FP *↓* (SD)				DP *↓* (SD)			
Forg. Ratio	0%	1%	5%	10%	0%	1%	5%	10%	0%	1%	5%	10%
Original	0.043	–	–	–	0.031	–	–	–	0.045	–	–	–
Retrained	–	0.040(0.006)	0.039(0.008)	0.043(0.016)	–	0.028(0.003)	0.027(0.004)	0.029(0.006)	–	0.038(0.006)	0.044(0.003)	0.042(0.004)
Unlearning Methods												
FU (Ours)	–	**0.041**(0.001)	**0.034**(0.002)	**0.032**(0.001)	–	**0.027**(0.003)	**0.025**(0.002)	**0.022**(0.001)	–	**0.038**(0.002)	**0.036**(0.001)	**0.032**(0.000)
GA	–	0.047(0.004)	0.045(0.006)	0.048(0.004)	–	0.032(0.005)	0.045(0.007)	0.044(0.006)	–	0.046(0.005)	0.056(0.007)	0.050(0.009)
CR	–	0.046(0.008)	0.048(0.007)	0.052(0.020)	–	0.034(0.008)	0.033(0.007)	0.038(0.014)	–	0.046(0.010)	0.041(0.008)	0.049(0.011)
CF	–	0.042(0.007)	0.040(0.003)	0.051(0.007)	–	0.041(0.004)	0.040(0.010)	0.032(0.012)	–	0.054(0.003)	0.052(0.011)	0.042(0.013)
SCRUB	–	0.067(0.024)	0.052(0.002)	0.058(0.017)	–	0.031(0.005)	0.030(0.004)	0.027(0.004)	–	0.043(0.004)	0.042(0.003)	0.040(0.003)
ORTHO	–	0.043(0.008)	0.039(0.005)	0.044(0.010)	–	0.043(0.008)	0.053(0.007)	0.055(0.001)	–	0.055(0.007)	0.064(0.006)	0.065(0.001)

Bold values indicate the best-performing result in each metric.

In contrast, our FU method has attained impressive performance in debiasing the original models towards more fair diagnostic decisions during the forgetfulness of medical records. Table 2 demonstrates that our FU has significantly mitigated the ethnicity-specific unfairness prominent in baseline unlearning methods across four NHS trusts and two pandemic waves. On the OUH wave 2 data, our FU demonstrates more substantial EO improvements over baselines with more data removed from the original model, i.e., EO-TP = 0.060 (original)  → 0.057, 0.049, 0.046, EO-FP = 0.052 (original)  → 0.049, 0.045, 0.044 and DP = 0.089 (original)  → 0.082, 0.079, 0.072 for the 1%, 5%, and 10% forgetting ratios, respectively, representing an overall improvement of 3%, 10%, and 18% compared to the best-performing baseline SCRUB. More favourably, even when compared to the model completely retrained on the remaining sets (training data excluding forget sets), models unlearned by our FU could still achieve commonly improved or at least comparable EOs, highlighting its efficiency and utility for clinical deployments. Similar trends can be observed from evaluation results on the PUH, UHB, and BH data of different pandemic waves, revealing FU’s strong generality and superiority for prospective and external applications. Notably, on BH (the second pandemic wave) and UHB (the first pandemic wave), the two external sites with more ethnically diverse cohorts, the proposed FU still delivers outstanding EO-TPs and EO-FPs, showing that the debiasing effects of the proposed fair unlearning mechanism can generalise well to varying clinical environments.

FU’s significant advantage in debiasing unlearned models is also evidenced by site-specific unfairness evaluations on the CURIAL-Combined dataset and also by ethnicity-specific inequality results on the eICU and MIMIC-IV datasets (Table 3). Specifically, on CURIAL-Combined, FU exhibits consistent hospital site fairness enhancement across increasing forgetting ratios, with EO-TP = 0.013 (original)  → 0.010, 0.012, 0.007, EO-FP = 0.023 (original)  → 0.017, 0.014, 0.010, DP = 0.058 (original)  → 0.053, 0.049, 0.046 for the 1%, 5%, and 10% forgetting ratios, achieving 26%, 43%, and 43% improvement over the best baseline GA. Turning to the eICU dataset, mortality prediction results demonstrate remarkable ethnicity fairness preservation even as more patient records are forgotten, where FU consistently outperforms the best baseline CR with improvements ranging from 25% to 49% across all forgetting ratios (EO-TP: 0.041  → 0.032, 0.039, 0.036; EO-FP: 0.014  → 0.017, 0.018, 0.013; DP: 0.021  → 0.019, 0.020, 0.019). Furthermore, extending to shock prediction on eICU, FU achieves substantial ethnicity bias reduction with progressively larger forgetting sets, delivering EO-TP = 0.070 (original)  → 0.058, 0.053, 0.048, EO-FP = 0.045 (original)  → 0.039, 0.041, 0.035, DP = 0.052 (original)  → 0.044, 0.038, 0.027, representing 14%, 30%, and 42% improvement over the best baseline CF, respectively. As for the MIMIC-IV dataset for mortality prediction, FU substantially mitigates ethnicity bias as the forgetting ratio increases, with EO-TP = 0.043 (original)  → 0.041, 0.034, 0.032, EO-FP = 0.031 (original)  → 0.027, 0.025, 0.022, DP = 0.045 (original)  → 0.038, 0.036, 0.032, corresponding to 21%, 22%, and 28% average improvements over the best baseline SCRUB.

**Fig. 1:**
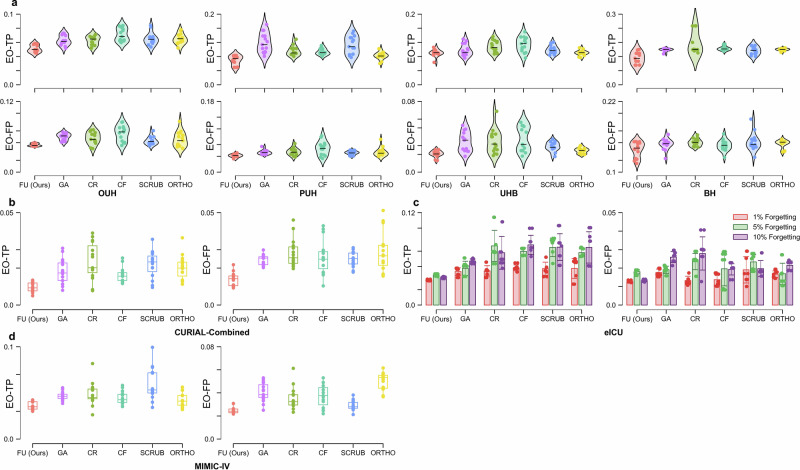
Algorithmic unfairness comparisons of various unlearning methods on four datasets. **a**. Violin plots of ethnicity-specific per-run EO-TP and EO-FP results across three forgetting ratios from the four NHS trusts of CURIAL (15 independent runs; 5 runs per forgetting ratio). **b**. Box plots of site-specific per-run EO-TP and EO-FP results across three forgetting ratios on the CURIAL-Combined dataset. **c**. Bar plots of ethnicity-specific per-run EO-TP and EO-FP results (average performance of mortality and shock predictions is reported) across three forgetting ratios on the eICU dataset. **d**. Box plots of ethnicity-specific per-run EO-TP and EO-FP results across three forgetting ratios on the MIMIC-IV dataset. Each box plot in **b,** **d** displays the median (central line), interquartile range (IQR; box), and 1.5 × IQR range (whiskers), obtained from 15 independent runs (5 runs per forgetting ratio). Each bar plot in **c** depicts the mean and standard deviation from 10 independent runs (5 runs per prediction task). Source data are provided as a Source Data file.

We visualise the per-run EO-TP and EO-FP values of various unlearning methods on the four CURIAL trusts, CURIAL-Combined, eICU (both tasks), and MIMIC-IV in Fig. 1a–d, respectively. The overall distribution of our FU results is consistently tighter and lower than others, implying a more stable and reliable equality improvement.

Generally, our FU has outperformed all machine unlearning baselines with the most unbiased performance across sensitive patient subgroups (ethnicities and hospital sites), DNN architectures (MLPs and LSTMs), EHR types (tabular or time-series), clinical tasks (rapid COVID-19 screening, mortality and shock predictions), and forgetting ratios. Even when compared to the fully retrained model, a method deemed as “golden solution” for unlearning but highly inefficient, FU can lead to surprisingly better, or at least matched, prediction equalities across protected subgroups. Those properties underline FU’s potential as a practical solution to mitigate algorithmic unfairness while clinically implementing RtbF.

### FU sufficiently preserves the clinical utility and protects the privacy of patients to be forgotten

**Fig. 2:**
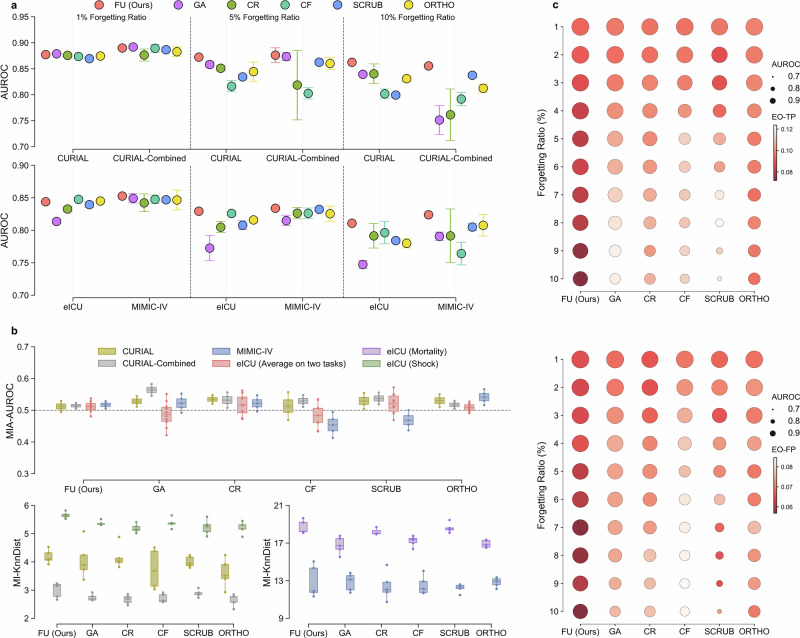
Diagnostic performance and unlearning effectiveness of various unlearning methods. **a** Comparisons of diagnostic performance of various machine unlearning methods on CURIAL, CURIAL-Combined, eICU, and MIMIC-IV datasets, categorised by the sizes of the forgetting sets (1, 5, and 10% forgetting ratios). Each point with the overlaid error bar shows the mean and SD from 5 independent runs. **b** Comparisons of the unlearning effectiveness of different methods on CURIAL, CURIAL-Combined, eICU, and MIMIC-IV datasets under the 10% forgetting ratio. MIA-AUROC values closer to 0.5 (dashed line) or higher MI-KnnDist values indicate more thorough forgetfulness and, thus, better privacy protection for the patients in the forgetting set. Each box plot displays the median (central line), IQR (box), and 1.5 × IQR range (whiskers), from 5 independent runs. **c** Bubble plots to illustrate the effects of varying forgetting set sizes, from 1% forgetting ratio to 10%, on the algorithmic fairness (EO-TP and EO-FP) and diagnostic performance (AUROC) of different unlearning methods on CURIAL. The mean EO-TP, EO-FP, and AUROC values of 5 independent runs across the four NHS trusts in CURIAL are reported. Deeper colour intensity represents better algorithmic fairness, while larger bubble size implies higher AUROC and better diagnostic performance. Source data are provided as a Source Data file.

In addition to improved algorithmic fairness, our FU also exhibited superior performance in the other two criteria of unlearning: effectively removing data from and preserving the diagnostic performance of the original model. We first report the prospective and external COVID-19 screening AUROCs of unlearned models across four NHS trusts in Table 4. It can be observed that several baseline unlearning methods, such as SCRUB, could lead to sharply decreased COVID-19 diagnostic performance, e.g., from the original model’s 0.880 (original) to 0.869, 0.834, 0.799 under 1, 5, and 10% forgetting ratios, representing an average drop of 1.3, 5.2, and 9.2% on the temporally prospective OUH cohorts of the second pandemic wave. Those AUROC drops indicate that those baselines have potentially triggered catastrophic forgetting in the original models and, thus, significantly impair their clinical utilities. On the other hand, our FU has substantially preserved the clinical utilities. For example, on the external BH cohorts of the second pandemic wave, models unlearned by FU present a slightly reduced AUROC of 0.887, 0.878, 0.863 under 1, 5, and 10% forgetting ratios, which are an improvement of 0.1, 1.6, and 3.5% compared to the best baseline GA. Similar promising screening performance can be found across the four CURIAL evaluation sets. More favourably, when compared to the entirely retrained models, our FU can still deliver unlearned models with comparable or slightly inferior AUROCs. AUROC results on the CURIAL-Combined (COVID-19 screening), eICU (mortality and shock predictions), and MIMIC-IV (mortality prediction) datasets can be found in Tables 4 and 5, signifying that the proposed FU could adequately mitigate the catastrophic forgetting phenomenon across clinical scenarios and tasks to retain the diagnostic performance after forgetfulness in medical AIs. We intuitively visualise the overall diagnostic performance of various unlearning methods across the four datasets in Fig. 2a.

**Table 4:** Diagnostic performance and unlearning effectiveness of various methods on CURIAL and CURIAL-Combined

CURIAL, Prospective evaluation on OUH										
	AUROC *↑* (SD) (OUH wave 2)				MIA-AUROC → 0.5 (SD)			MI-KnnDist *↑* (SD)		
Forgetting Ratio	0%	1%	5%	10%	1%	5%	10%	1%	5%	10%
Original	0.880	–	–	–	–	–	–	–	–	–
Retrained	–	0.876(0.001)	0.876(0.001)	0.875(0.002)	0.521(0.002)	0.503(0.002)	0.511(0.002)	3.73(0.331)	4.28(0.443)	4.33(0.750)
Unlearning Methods										
FU (Ours)	–	0.877(0.000)	**0.872**(0.000)	**0.862**(0.000)	0.746(0.019)	**0.657**(0.016)	**0.512**(0.013)	3.67(0.499)	**4.19**(0.169)	**4.19**(0.242)
GA	–	**0.879**(0.001)	0.858(0.006)	0.839(0.003)	0.801(0.010)	0.707(0.026)	0.528(0.013)	3.45(0.457)	4.00(0.982)	4.06(0.652)
CR	–	0.876(0.003)	0.851(0.006)	0.840(0.021)	0.767(0.015)	0.671(0.028)	0.534(0.010)	3.53(0.426)	4.11(1.011)	4.17(0.415)
CF	–	0.874(0.004)	0.816(0.012)	0.802(0.011)	0.795(0.010)	0.712(0.014)	0.513(0.034)	3.61(0.246)	4.18(0.669)	3.75(0.683)
SCRUB	–	0.869(0.001)	0.834(0.005)	0.799(0.005)	0.800(0.022)	0.741(0.025)	0.529(0.019)	**3.86**(0.588)	3.63(0.667)	4.00(0.171)
ORTHO	–	0.874(0.003)	0.844(0.021)	0.831(0.006)	**0.726**(0.024)	0.696(0.014)	0.530(0.016)	3.52(0.354)	3.41(0.472)	3.60(0.500)

CURIAL, External evaluations												
	AUROC *↑* (SD) (PUH wave 2)				AUROC *↑* (SD) (UHB wave 1)				AUROC *↑* (SD) (BH wave 2)			
Forgetting Ratio	0%	1%	5%	10%	0%	1%	5%	10%	0%	1%	5%	10%
Original	0.856	–	–	–	0.885	–	–	–	0.890	–	–	–
Retrained	–	0.857 (0.002)	0.856 (0.001)	0.855 (0.002)	–	0.884 (0.002)	0.884 (0.004)	0.883 (0.004)	–	0.895(0.002)	0.894(0.003)	0.894(0.004)
Unlearning Methods												
FU (Ours)	–	**0.851** (0.000)	**0.842** (0.001)	**0.830** (0.001)	–	**0.884** (0.000)	**0.875** (0.000)	**0.865** (0.001)	–	**0.887** (0.000)	**0.878** (0.000)	**0.863** (0.000)
GA	–	0.851 (0.001)	0.827 (0.005)	0.808 (0.002)	–	0.881 (0.001)	0.866 (0.004)	0.847 (0.003)	–	0.886 (0.003)	0.864 (0.007)	0.834 (0.003)
CR	–	0.851 (0.004)	0.824 (0.008)	0.810 (0.027)	–	0.879 (0.006)	0.855 (0.011)	0.846 (0.025)	–	0.888 (0.002)	0.871 (0.004)	0.858 (0.018)
CF	–	0.849 (0.002)	0.785 (0.013)	0.771 (0.012)	–	0.878 (0.004)	0.815 (0.022)	0.803 (0.033)	–	0.878 (0.007)	0.809 (0.015)	0.796 (0.011)
SCRUB	–	0.847 (0.001)	0.816 (0.007)	0.781 (0.010)	–	0.869 (0.003)	0.826 (0.008)	0.785 (0.012)	–	0.875 (0.004)	0.840 (0.007)	0.790 (0.012)
ORTHO	–	0.851 (0.004)	0.834 (0.003)	0.829 (0.007)	–	0.880 (0.005)	0.863 (0.010)	0.846 (0.007)	–	0.886 (0.002)	0.870 (0.015)	0.857 (0.009)

CURIAL-Combined										
	AUROC *↑* (SD)				MIA-AUROC → 0.5 (SD)			MI-KnnDist *↑* (SD)		
Forgetting Ratio	0%	1%	5%	10%	1%	5%	10%	1%	5%	10%
Original	0.907	–	–	–	–	–	–	–	–	–
Retrained	–	0.904(0.001)	0.904(0.001)	0.905(0.001)	0.527(0.021)	0.521(0.026)	0.518(0.014)	3.22(0.109)	3.31(0.089)	3.43(0.079)
Unlearning Methods										
FU (Ours)	–	0.890(0.002)	**0.876**(0.016)	**0.855**(0.001)	**0.659**(0.013)	**0.590**(0.018)	**0.514**(0.007)	**2.50**(0.178)	**2.98**(0.139)	**3.01**(0.266)
GA	–	**0.892**(0.003)	0.873(0.008)	0.751(0.031)	0.708(0.021)	0.644(0.026)	0.564(0.014)	2.21(0.133)	2.62(0.157)	2.74(0.116)
CR	–	0.876(0.013)	0.819(0.075)	0.761(0.055)	0.733(0.012)	0.620(0.007)	0.532(0.019)	2.32(0.137)	2.64(0.098)	2.67(0.155)
CF	–	0.889(0.008)	0.802(0.012)	0.792(0.015)	0.731(0.027)	0.681(0.027)	0.529(0.014)	2.34(0.132)	2.71(0.162)	2.71(0.156)
SCRUB	–	0.887(0.004)	0.863(0.005)	0.837(0.004)	0.716(0.019)	0.639(0.017)	0.537(0.014)	2.21(0.135)	2.64(0.142)	2.88(0.125)
ORTHO	–	0.883(0.010)	0.860(0.013)	0.812(0.009)	0.682(0.011)	0.631(0.016)	0.517(0.010)	2.28(0.156)	2.62(0.101)	2.62(0.204)

Prospective and external evaluations are conducted on the four UK NHS trusts of CURIAL. Diagnostic performance is evaluated by AUROC, and unlearning effectiveness is evaluated by MIA-AUROC and MI-KnnDist. Note that MIA-AUROC and MI-KnnDist are privacy attack results on the forgetting sets from the training data. Bold values indicate the best-performing result in each metric.

**Table 5:** Diagnostic performance and unlearning effectiveness of various methods on eICU and MIMIC-IV

eICU										
	AUROC *↑* (SD)				MIA-AUROC → 0.5 (SD)			MI-KnnDist *↑* (SD)		
Forgetting Ratio	0%	1%	5%	10%	1%	5%	10%	1%	5%	10%
Mortality Prediction										
Original	0.853	–	–	–	–	–	–	–	–	–
Retrained	–	0.847(0.003)	0.847(0.002)	0.847(0.002)	0.504(0.021)	0.483(0.020)	0.503(0.014)	20.80(0.249)	20.98(0.434)	21.29(0.283)
Unlearning Methods										
FU (Ours)	–	0.844(0.001)	**0.829**(0.002)	**0.811**(0.001)	0.617(0.017)	**0.506**(0.012)	0.514(0.011)	**17.97**(0.605)	**19.02**(0.453)	**18.68**(0.710)
GA	–	0.814(0.004)	0.773(0.022)	0.747(0.007)	0.702(0.022)	0.565(0.012)	0.463(0.032)	12.62(0.930)	15.40(0.410)	16.77(0.898)
CR	–	0.833(0.004)	0.805(0.009)	0.791(0.021)	0.665(0.009)	0.572(0.007)	0.515(0.040)	12.41(0.765)	18.22(0.461)	18.22(0.416)
CF	–	**0.848**(0.003)	0.826(0.003)	0.796(0.019)	**0.617**(0.013)	0.640(0.012)	0.484(0.038)	13.13(1.042)	14.37(0.856)	17.21(0.536)
SCRUB	–	0.840(0.005)	0.808(0.008)	0.784(0.003)	0.744(0.003)	0.709(0.003)	0.531(0.032)	12.42(0.795)	16.06(1.033)	18.62(0.510)
ORTHO	–	0.845(0.003)	0.816(0.004)	0.780(0.008)	0.665(0.009)	0.617(0.009)	**0.506**(0.012)	15.35(0.866)	16.02(0.604)	16.91(0.395)
Shock Prediction										
Original	0.862	–	–	–	–	–	–	–	–	–
Retrained	–	0.859(0.002)	0.858(0.001)	0.856(0.002)	0.503(0.021)	0.479(0.020)	0.504(0.014)	6.25(0.067)	6.07(0.149)	6.19(0.119)
Unlearning Methods										
FU (Ours)	–	**0.853**(0.000)	**0.841**(0.000)	**0.801**(0.000)	**0.585**(0.019)	**0.492**(0.010)	**0.509**(0.022)	**5.61**(0.176)	**5.62**(0.125)	**5.66**(0.109)
GA	–	0.845(0.002)	0.824(0.029)	0.749(0.038)	0.639(0.018)	0.544(0.017)	0.511(0.031)	4.31(0.350)	5.23(0.188)	5.37(0.085)
CR	–	0.852(0.002)	0.824(0.025)	0.788(0.036)	0.598(0.013)	0.525(0.008)	0.517(0.035)	4.58(0.301)	5.28(0.249)	5.19(0.149)
CF	–	0.846(0.006)	0.822(0.002)	0.790(0.008)	0.614(0.011)	0.642(0.012)	0.484(0.039)	5.50(0.270)	5.29(0.300)	5.38(0.175)
SCRUB	–	0.842(0.002)	0.806(0.004)	0.773(0.010)	0.784(0.003)	0.680(0.003)	0.510(0.032)	4.81(0.102)	5.17(0.213)	5.23(0.262)
ORTHO	–	0.845(0.002)	0.826(0.003)	0.784(0.018)	0.632(0.009)	0.584(0.009)	0.512(0.012)	4.69(0.312)	5.04(0.153)	5.23(0.214)

MIMIC-IV										
	AUROC *↑* (SD)				MIA-AUROC → 0.5 (SD)			MI-KnnDist *↑* (SD)		
Forgetting Ratio	0%	1%	5%	10%	1%	5%	10%	1%	5%	10%
Original	0.870	–	–	–	–	–	–	–	–	–
Retrained	–	0.860(0.006)	0.859(0.002)	0.856(0.004)	0.492(0.010)	0.512(0.007)	0.499(0.002)	12.88(1.287)	12.71(0.858)	12.95(1.910)
Unlearning Methods										
FU (Ours)	–	**0.853**(0.002)	**0.834**(0.000)	**0.824**(0.000)	**0.753**(0.026)	**0.614**(0.017)	**0.517**(0.009)	12.54(0.525)	**12.61**(0.312)	**12.88**(1.691)
GA	–	0.849(0.008)	0.815(0.009)	0.791(0.007)	0.775(0.031)	0.686(0.019)	0.522(0.023)	12.38(1.000)	12.20(0.783)	12.86(0.929)
CR	–	0.842(0.015)	0.826(0.010)	0.791(0.046)	0.763(0.021)	0.638(0.014)	0.522(0.019)	**12.66**(1.162)	11.97(0.657)	12.42(1.473)
CF	–	0.848(0.007)	0.826(0.009)	0.764(0.020)	0.791(0.019)	0.687(0.010)	0.454(0.032)	12.17(0.625)	12.37(1.078)	12.51(0.998)
SCRUB	–	0.847(0.004)	0.832(0.003)	0.805(0.006)	0.789(0.010)	0.749(0.033)	0.468(0.025)	12.05(1.237)	12.15(1.060)	12.22(0.448)
ORTHO	–	0.847(0.017)	0.826(0.013)	0.808(0.019)	0.808(0.010)	0.701(0.013)	0.541(0.019)	12.08(0.503)	11.94(0.857)	12.84(0.491)

Diagnostic performance is evaluated by AUROC, and unlearning effectiveness is evaluated by MIA-AUROC and MI-KnnDist. Note that MIA-AUROC and MI-KnnDist are privacy attack results on the forgetting sets from the training data. Bold values indicate the best-performing result in each metric.

We thoroughly examine the effects of varying forgetting set sizes on the diagnostic performance and algorithmic unfairness by visualising the average performance across four CURIAL trusts, from 1% forgetting ratio to 10% with a 1% increase. Figure 2c portrays the bubble plot with colour representing algorithmic unfairness (EO-TP and EO-FP) and bubble size for diagnostic performance (AUROC). We can intuitively conclude that, as forgetting ratios gradually increase, our FU method consistently demonstrates larger bubble sizes (higher diagnostic AUROC) with deeper colour saturation (more fair predictions). In contrast, those baseline methods exhibit significantly shrunk bubbles (reduced diagnostic AUROC) and lighter colours (less algorithmic fairness). Therefore, our FU has achieved robust, reliable, and generalised fairness and utility improvements over baselines across forgetting sets of varying sizes.

We measure the unlearning effectiveness as MIA-AUROC and MI-KnnDist values, and generally, the closer MIA-AUROC is to 0.5, or the higher MI-KnnDist is, the better privacy protection (of the removed patients) against MIA or MI it indicates. MIA-AUROC and MI-KnnDist scores across the CURIAL, CURIAL-Combined, eICU, and MIMIC-IV datasets are presented in Tables 4 and 5. We could observe that as more records are removed (forgetting ratios increase), MIA-AUROC values are closer to 0.5, and MIA-AUROC values also increase, indicating a more thorough forgetfulness. A possible reason for this trend is that when the forgetting set size is small, most pre-learned knowledge in the original model will still be preserved after unlearning. As a result, the knowledge distribution of the unlearned model is still close to the original one, and it would be comparably easier to distinguish the forgotten samples from other unseen ones. On the other hand, removing a larger set from DNNs will more significantly alter their inherent knowledge structures and, therefore, the forgotten records will be more likely to be viewed as the new data with MIA-AUROC values closer to 0.5 or higher MI-KnnDists. Despite the MIA-AUROC or MI-KnnDist fluctuations with forgetting set sizes, our FU demonstrates the overall best unlearning effectiveness, with most MIA-AUROC values closest to 0.5 and generally best MI-KnnDists compared to all baselines across datasets and clinical tasks. We compare the unlearning effectiveness of various methods on CURIAL, CURIAL-Combined, eICU, and MIMIC-IV under the 10% forgetting ratio in Fig. 2b, and it can be intuitively discovered that our FU can yield unlearned models with generally better privacy protections than others when under membership inference or model inversion attacks on the medical records to be removed. Additional experimental results are provided in Supplementary Note 3.

**Fig. 3:**
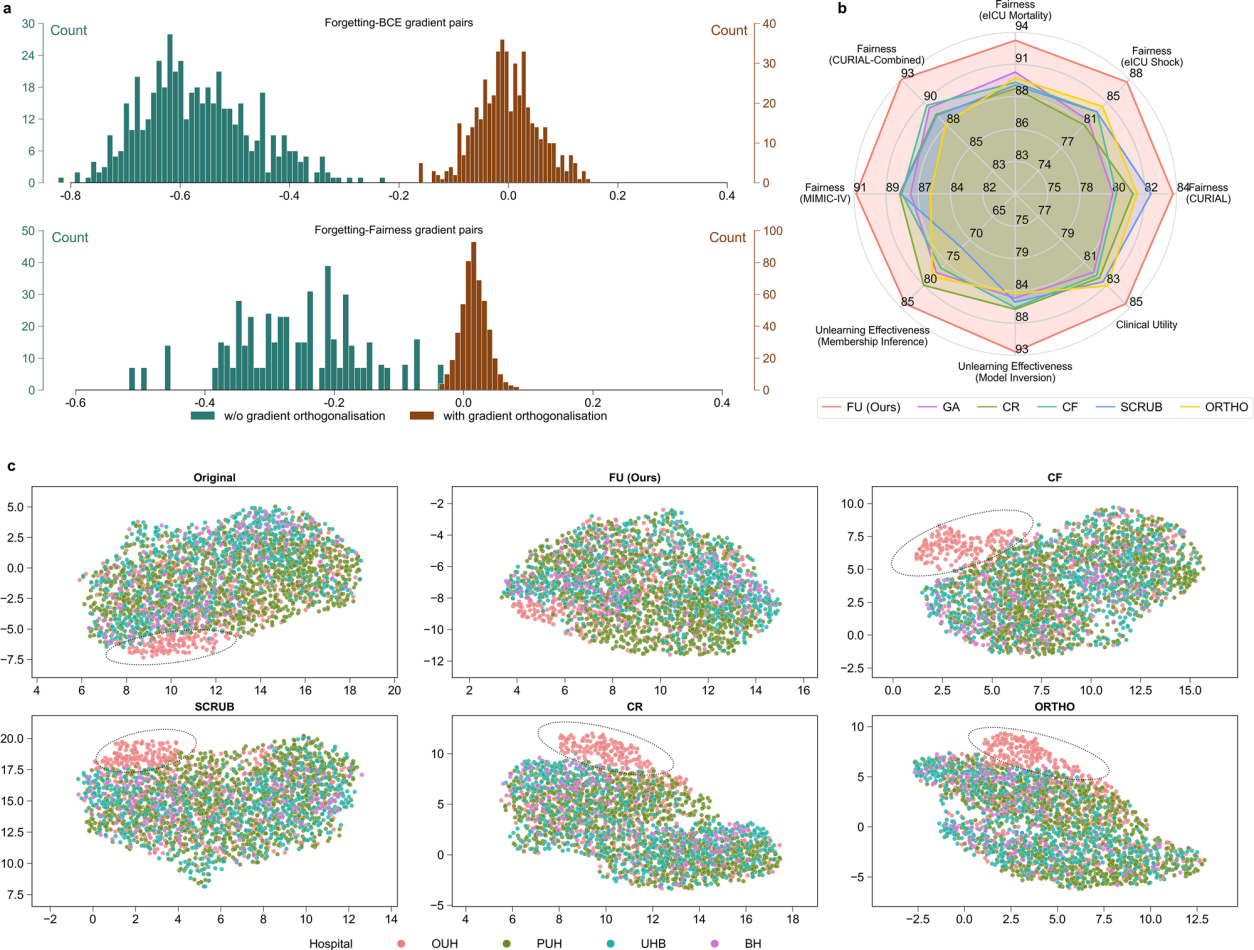
Algorithmic insight analyses and overall performance. **a** Histogram plots of the cosine similarities between gradient pairs from potentially conflicting objectives, with or w/o gradient orthogonalisations. The top shows gradient pairs from the forgetting and BCE losses. Bottom shows gradient pairs from the forgetting and fairness losses. **b** The proposed FU achieves superior performance than prevalent unlearning baselines on clinical utility, unlearning effectiveness, and, more desirably, algorithmic fairness across various real-world medical datasets and clinical tasks. Each radii in the radar plot represents a normalised, overall score of a specific unlearning criterion averaged across the 1, 5, and 10% forgetting ratios, which is the higher, the better. **c** t-SNE visualisations on the COVID-19 positive cases of the CURIAL-Combined dataset. Data points are coloured by the four hospital sites. t-SNE representations are from: the original model, and unlearned models by FU (ours), CF, SCRUB, CR, and ORTHO, respectively. Source data are provided as a Source Data file.

We provide a comprehensive performance summarisation of all unlearning methods in Fig. 3b. Each radii in the radar plot represents a normalised, overall score of a specific unlearning criterion. We summarise the average performance of clinical utility and unlearn effectiveness across all four datasets and three forgetting ratios, while algorithmic fairness is more detailedly illustrated by specific datasets and tasks since it is the focus of this work. Our FU has demonstrated remarkable improvement over all unlearning baselines on all the radii scores, clearly showing its universal advantages for clinical employment. We thoroughly describe the conversions from unlearning metrics to radii scores in the plot in Section “Methods”.

### Algorithmic insights behind fair unlearning: benefits of orthogonalities in the gradient space

We further analyse the algorithmic insights of our FU method to better illustrate the underlying mechanism that drives the unfairness mitigation. We attribute this improvement to a key design in FU: enforcing the orthogonalities of gradients from different objectives. Specifically, three main objectives are adopted in FU: the usual machine learning objective (binary cross-entropy or BCE loss), the unlearning objective (forgetting loss), and the equality objective (fairness loss). When updating a DNN with the gradients from above, it is conceivable that the gradient of one objective could conflict with that of another of an inconsistent or even contrary purpose. Therefore, we propose maintaining orthogonalities in the gradient space to minimise the potentially conflicting impacts between varying objectives. That is, each objective’s gradient is constrained to be orthogonal to those of others, which indicates mathematically independent and, therefore, could lead DNN to fulfil each potentially conflicting functionality faithfully, smoothly, and undisturbedly. Moreover, our orthogonalisation design naturally alleviates catastrophic forgetting by ensuring that the unlearning process does not interfere with previously learned knowledge representations. As a result, our FU could simultaneously attain superior unlearning effectiveness, preserved clinical utility, and unfairness mitigation as depicted in Fig. 3b.

To better illustrate the importance of maintaining gradient orthogonalities, we visualise the histograms of similarities of gradient pairs (measured by cosine similarities) of different objectives with or w/o gradient orthogonalisations. Figure 3a plots gradient pairs from the forgetting loss (for unlearning) and BCE loss (for clinical utility), and it can be discovered that w/o gradient orthogonalisations (as in most unlearning baselines), the histograms of their cosine similarities exhibit a heavily negative distribution, implying that most gradient pairs will yield inconsistent or conflicting parameter updates that will negatively impact the model’s performance. On the other hand, applying our gradient orthogonalisations has significantly altered the overall distribution of cosine similarities of gradient pairs to centre around 0. This distinct pattern discloses that our FU’s gradient orthogonalisation has sufficiently prevented the conflicts from unlearning and predictive objectives. Similar observations can be made on gradient pairs from the forgetting and fairness losses in Fig. 3a, which substantially verifies the positive effects of the orthogonality-preserving mechanism on the unlearning and fairness objectives.

We further inspect fairness-related evidence to intuitively understand the effects of gradient orthogonalisations on unlearned models. As shown in Fig. 3c, we adopt t-stochastic neighbour embedding^35^ (t-SNE) to obtain the 2D representations of all positive COVID-19 cases in CURIAL-Combined from the original model, our FU, CF, SCRUB, CR, and ORTHO, respectively. We colour the t-SNE representations of each patient by the NHS trust they attended. We can read from Fig. 3c that t-SNE embeddings from the original model announce a highly distinguishable cluster of a specific site (OUH), marked by a dotted circle. This prominent t-SNE cluster arguably reveals that the original model can potentially rely on hospital-specific features, such as data collection tools, ways of data annotations, curations, and pre-processings, etc., to predict the COVID-19 status of patients, and thus, represent a notable degree of site-specific unfairness. As depicted in Fig. 3c, t-SNE representations of unlearned models with CF, SCRUB, CR, and ORTHO picture a similarly distinct OUH cluster but covering a broader region with more records. This phenomenon intuitively evidences that forgetfulness of medical records with baseline unlearning methods will amplify models’ reliance on site-specific features, yielding diagnostic results with exacerbated inequalities. In contrast, the t-SNE embedding of our FU in Fig. 3c delivers a distribution essentially unclusterable by hospital sites. This pattern reasonably implies that the unlearned model by our FU, thanks to the gradient orthogonalisations, has sufficiently debiased the original model after unlearning to attain a site-independent diagnosis across four NHS trusts.

**Fig. 4:**
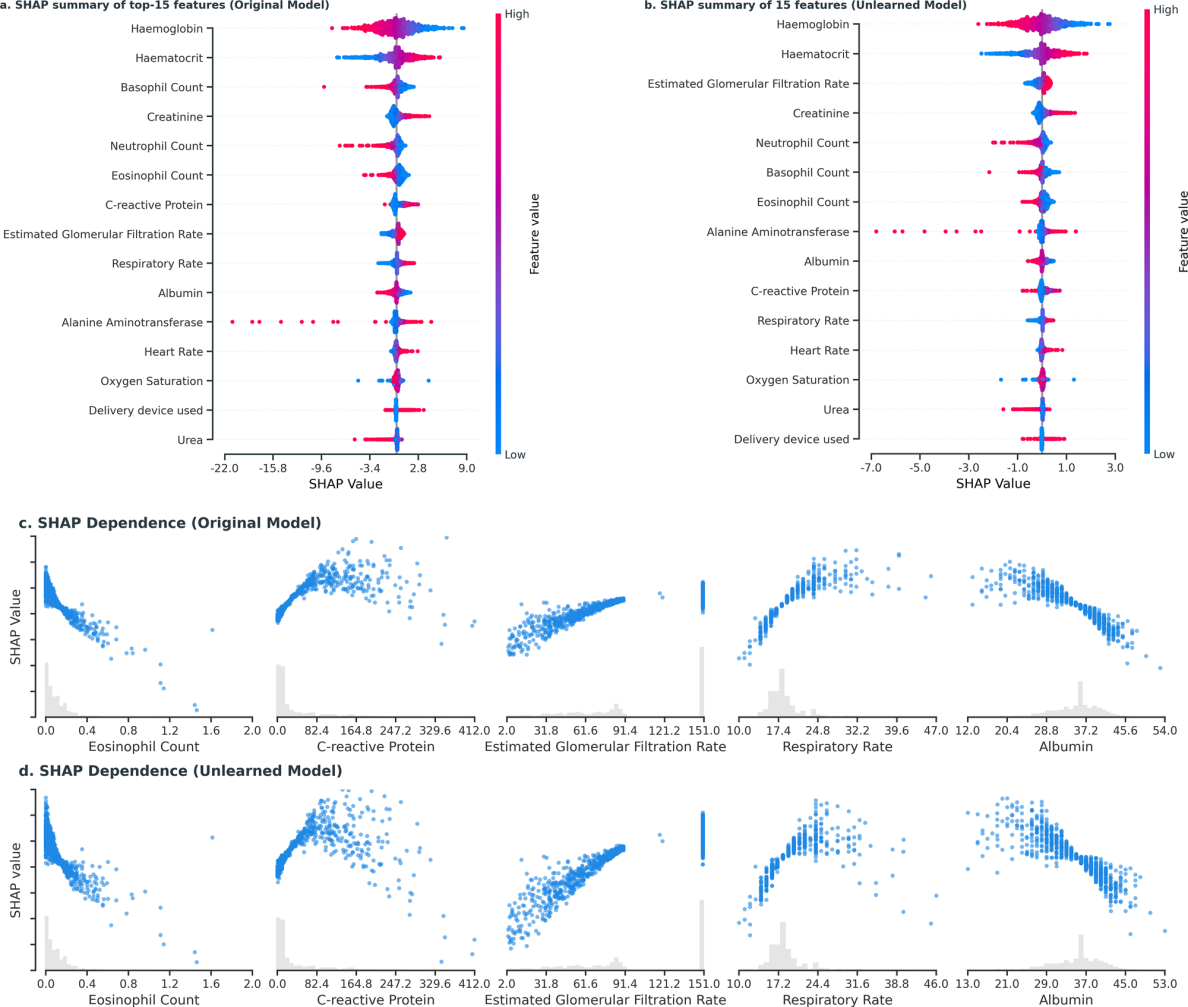
SHAP-based explainability of the original and unlearned models. **a**,** b** SHAP summary beeswarm plots for the original model and the unlearned model. Variables are ordered by the mean absolute SHAP value, and the top 15 ones are displayed. **c**,** d** SHAP dependence plots for the top-5 variables with the most significant ranking variants before and after unlearning, with points coloured by raw feature values. Analyses are conducted on the original and unlearned models for predicting COVID-19 infections on 1024 patient records from the OUH wave 2 cohort; each dot represents a single patient record, and positive SHAP values indicate a change in the model’s prediction towards a positive COVID-19 outcome. Source data are provided as a Source Data file.

We adopt Shapley Additive exPlanations (SHAP)^36^ to illustrate the effects of individual features on the model predictions for COVID-19 infections, before and after unlearning 10% training data with our FU, on the 1024 patient records of the OUH wave 2 cohort. The SHAP summary beeswarm plots for the top 15 most important features between the original and unlearned models are shown in Fig. 4a–b. We can see that the unlearning operation does not significantly alter the clinical explainability of model predictions. For example, “Haemoglobin” and “Haematocrit” remained the two most important clinical variables for determining infection status, while the effects of “Estimated Glomerular Filtration Rate”, though ranked higher after forgetting, still exhibit a generally similar trend as the original model predictions. No substantial changes in explainability are observed in other top influential variables, despite some ranking differences. We further investigate the five clinical variables with the most significant ranking variants after unlearning: “Eosinophil Count”, “C-reative protein”, “Estimated Glomerular Filtration Rate”, “Respiratory Rate”, and “Albumin”, by plotting their SHAP dependence plots in Fig. 4c, d. As can be seen from the plots, despite slight or moderate changes in several data points, removing medical records using our FU does not significantly alter the overall SHAP dependence trends of the clinical variables. These pieces of evidence imply that our FU algorithm does not significantly alter or impair clinical explainability, and that post-unlearning models could still provide feature importance explanations consistent with the original models to support clinical decision-making.

## Discussion

RtbF in GDPR evolves from the need to forget in our everyday lives and establishes the legal grounds to safeguard patients’ rights to have their data deleted from healthcare organisations and systems. Since the first day of RtbF’s introduction, there have been unceasing social and philosophical discussions and debates around its ambiguities, legal scopes, and more^5,37,38^. However, the impacts of RtbF in intelligent healthcare are still a less-discussed field^39^ that requires more profound and thorough investigations. In this work, we inspect RtbF through the lens of clinical AI, revealing one unpictured yet critical outcome of its clinical implementations: exacerbated healthcare inequalities. Existing machine unlearning methods, as an essential tool to remove patient data from intelligent algorithms and models, are discovered to amplify the unfairness of AI-based diagnosis across sensitive subpopulations based on our extensive studies.

The societal impacts of such undesirable amplifications are two-fold. After data removal, clinical AI models will yield more discriminatory decisions, negatively influencing those underrepresented patients who are already suffering from healthcare disparities. For instance, if an AI system is deployed to prescribe medications for common diseases, and specific patient records are occasionally deleted from the AI model with unlearning algorithms upon requests, patients of minority ethnicities could gradually find themselves receiving fewer treatment opportunities than the majority. Accumulations of such algorithmic unfairness will, in the long-term view, inevitably exacerbate healthcare inequalities with less-satisfying treatments and outcomes among socioeconomically disadvantaged people. The resulting public distrust in AI-based products and services will unquestionably dim the future of intelligent healthcare. Another negative impact could be the increased risks of privacy leaks from statistical disclosures. Minority groups are generally more vulnerable to re-identification risks due to their small population size. A sufficiently fair model can gain extra biases and exhibit less satisfying accuracy on the minorities; the overly poor performance on the minority cohorts will introduce additional re-identification risks for them, as there may be very few cases of a sensitive attribute in a particular region, such as a given ethnicity.

Overall, the amplified algorithmic inequalities from unlearning algorithms can incur detrimental long-term societal impacts and represent a de facto violation of another ethical requirement of AI studies: the fairness principle. We, therefore, formalise the conflict between RtbF and the fairness rule as a crucial, fundamental dilemma to be resolved towards more trustworthy and privacy-preserving clinical AI systems. As such, we present the first fair unlearning system for clinical AI as a generalised and robust solution for the RtbF-fairness dilemma. The evaluation of unlearning algorithms is a multi-dimensional issue, and we comprehensively examine three crucial properties of medical AI models after forgetfulness: the clinical utility, the unlearning effectiveness, and algorithmic fairness. The experimental results on four large-scale, real-world clinical datasets, CURIAL, CURIAL-Combined, eICU, and MIMIC-IV, demonstrate the superiority of our fair unlearning framework on all three unlearning criteria across diverse clinical tasks. The diagnostic performance of the original model is adequately preserved after removing records, and the privacy of the patients to be forgotten is also effectively protected. More notably, our FU significantly mitigates the ethnicity-specific and site-specific algorithmic unfairness of unlearned models. Those desirable properties of FU open a new gate for clinical practice in the digital era that patient records can be removed for artificial intelligence systems in a clean, efficient, and unbiased manner. To the best of our knowledge, our FU accommodates the first practical solution for the RtbF-fairness dilemma in clinical AI, which potentially embeds worldwide impacts on every stakeholder in the healthcare ecosystem.

In the AI community, there are a few recent works on machine unlearning that use orthogonal constraints to address the potential conflicts between the forgetting and learning objectives, thereby improving predictive performance. ORTHO^31^ performs per-sample gradient orthogonalisation by projecting forgetting gradients onto the orthogonal subspace of gradients on the remaining set, which is by far the most related work to our FU. However, it emphasises only the predictive performance of unlearned models in automatic speech recognition (ASR) and ignores the crucial dimensionality of algorithmic equality and privacy protections. GDR-GMA^40^ adopts a multi-task learning perspective for machine unlearning, rectifying conflicting gradients by projecting them onto orthogonal planes while dynamically adjusting gradient magnitudes. FG-OrIU^41^ imposes orthogonal constraints at both feature and gradient levels, dynamically adjusting subspaces during incremental unlearning to prevent knowledge reintroduction and maintain stability between the forgetting and retaining samples. Neither of them, however, attempts to address the algorithmic unfairness that could lead to societal inequalities across patient subgroups, nor to address the AI ethical dilemma between RtbF and fairness. Our FU are the first to optimise algorithmic fairness during machine unlearning via gradient orthogonalisations, especially in the healthcare sector.

Several interesting observations can be made from our experimental results and deserve further discussion. The size of the forgetting sets, as reported in Section “Results”, could affect the performance of unlearning algorithms. A common trend is that the more data is removed, the more diagnostic performance will be lost. All unlearning methods obey this empirical rule: unlearned models of a 1% forgetting ratio commonly yield higher AUROC than those of 10%. A contrastive trend can be found in measurements of unlearning effectiveness, in which a larger forgetting size will introduce a more thorough data removal. Those observations reveal a trade-off relationship between clinical utility and unlearning effectiveness in intelligent healthcare models, and we intuitively explain this relationship to the inherent knowledge variations before and after forgetfulness. Removing a few records will not significantly transform the pre-learned knowledge of the clinical data, which could still guide DNNs to yield diagnostic performance close to the original. This preserved knowledge structure, however, also represents a higher possibility of membership inference on forgotten data. For a given sample, we may find several similar cases still preserved in the memory of unlearned models, and thus, it will be possible to infer its original membership with those cases as anchors. On the other hand, the forgetfulness of many more records will lead to a degraded, remarkably altered knowledge structure with deteriorated clinical utilities and better privacy safeguarding. Notably, an overly dropped diagnostic performance marks the occurrence of catastrophic forgetting, a common phenomenon among unlearning baselines. With the unique gradient orthogonalisation mechanism, unlearned models by our FU can maintain a proper, less-transformed understanding of the clinical knowledge in the original data to mitigate the catastrophic forgetting issue and retain sufficient diagnostic performance.

Algorithmic unfairness across the forget set sizes represents distinct distributions between baseline unlearning methods and our FU. While all baselines have exacerbated the inequalities of predictions of unlearned models, our FU could instead reduce the discriminatory degrees of AI models across sensitive subgroups. This observation unveils a unique and favourable trait of our FU, i.e., it is “debiasing towards forgetfulness”. This debiasing effect originates from the process of reshaping the model’s knowledge space with the fairness-guided objective, which provides additional opportunities for DNNs to reflect and adjust their predictive biases. Generally, the more data is forgotten, the more fair the decisions are, but with degraded diagnostic performance. This trend reveals the trade-off relationship between clinical utility and algorithmic fairness. Although recent AI works have verified the existence of the utility-fairness trade-off in machine learning scenarios^42,43^, we are the first to achieve and report a similar relationship in the context of machine unlearning, with a carefully designed fair unlearning system governed by gradient orthogonalisation. Clinically, it indicates that the forgetfulness of more medical records could yield AI models with reduced utility but more fair diagnostic decisions. The impaired utilities can be compensated in future studies to attain clinically satisfying performance, and, in the long run, applying the fair unlearning strategy could accumulatively and effectively mitigate AI-exacerbated healthcare disparities to facilitate equitable care and win patient trust.

In real-world clinical scenarios, the unique utility-fairness trade-off of our FU also underscores the importance of setting practical thresholds for practitioners to determine whether retraining is required after multiple rounds of unlearning operations. Following the existing results on the 1%, 5%, and 10% forgetting ratios and an extensive experiment on a broader range of forgetting ratios from 1% to 25% (see Supplementary Fig. 5), model utility can be a more critical metric for determining whether a full retraining is necessary, depending on the clinical scenarios encountered upon deployment. For example, a tiered monitoring system can be adopted on the predictive performance of the unlearning model with the following rules: a 3–5% AUROC drop will trigger a yellow warning flag indicating more intensive monitoring, a 5–10% AUROC drop will incur a red flag for re-investigating the necessity of retraining, and a drop exceeding 10% will launch a retraining to be conducted immediately. Additional flagging systems for fairness or privacy protection metrics can be developed with similar criteria for performance drop and combined to support more comprehensive, real-time monitoring. However, it is worth noting that those criteria are set following empirical rules, and the complexity and challenges of clinical deployments will require those thresholds to be adjusted accordingly.

Another interesting issue to discuss for clinical deployment is how to communicate the status of unlearning performance to clinicians. There can be several options depending on the budget, available hardware, specific requirements, etc. For instance, with limited budgets, the status can be displayed as simple alert information via terminals or logs each time an unlearning operation is completed. A more sophisticated, detailed dashboard can be developed, if necessary, to provide real-time monitoring of fairness metrics, unlearning effectiveness, diagnostic performance, and other relevant information, organised into tables, graphs, indicators, and related visualisations. This dashboard can be displayed on required devices, such as the central monitors of a care unit and wearable devices for doctors. Conversational interfaces can also be considered, in which clinicians can directly consult the overall situations and seek advice from chatbots, e.g., whether unlearning is effective, or whether retraining should be done. Other IT communication tools, such as emails and Messages, can be used to deliver summary and alerting information to clinicians when necessary.

While we have examined algorithmic unfairness across different sensitive attributes, real-world disparities may also arise from intersectional unfairness, i.e., societal inequalities across subpopulations stratified by joint attributes, which is mostly overlooked in prior works. To investigate this issue, we evaluate the performance of various unlearning methods against intersectional unfairness on the CURIAL dataset by jointly stratifying patients by ethnicity and age groups (results outlined in Supplementary Tables 12 and 13). Generally, we have shown that our FU can still generalise well to the ethnicity-age intersectional unfairness on the CURIAL dataset, achieving superior fairness, unlearning effectiveness, and model utility compared to unlearning baselines across the four NHS trust cohorts in CURIAL, thereby increasing our FU’s potential for mitigating real-life disparities. Investigating how unlearning methods could generalise to other clinical tasks and intersectional inequalities could be an interesting future work.

As the pioneering strategy for fair unlearning, our FU system still encloses several limitations at the current stage. FU is devised as an unlearning algorithm that, in theory, can be universally translated to various clinical routine scenarios. Three real-world tasks, COVID-19 screening, mortality, and shock predictions, have arguably verified the generality of the proposed framework on prospective and external patients. However, the complexity and extensiveness of clinical practices still call for broader future validations of its universality through more diverse medical scenarios, e.g., regression-based diagnosis and prognoses, studies of rare diseases, etc.

Although we have already demonstrated the unlearning effectiveness of our FU method using two typical privacy-attacking schemes, i.e., membership inference and model inversion, we would like to note that there are still multiple other ways of attempting to extract sensitive information from a DNN model, such as property inference^44,45^ and model extraction attacks^46,47^. Due to the complexity and vastness of existing attack methods, we are unable to perform an exhaustive evaluation of the privacy-preservation capabilities of unlearning methods across all plausible paradigms, which is a potential limitation of this work.

The real-world deployment of the unelarning framework will also raise additional, challenging privacy concerns and beyond. For example, the federated learning scenario^48,49^ will pose challenges in the management and implementation of unlearning requests. How to perform unlearning in a collaborative learning environment with adequate privacy preservation can be an interesting topic with practical clinical value for future research. In real-world applications, potential privacy leak risks can also accumulate as multiple batches of forgetting requests are processed in sequence. Multiple unlearning procedures will typically yield multiple opportunities for privacy attacks, representing a greater risk of sensitive information leak than a single one. An adversary may adopt the model output differences across the sequential unlearning operations to infer the removed records. As such, developing a practical guide on how to eliminate privacy leaks comprehensively and effectively can be a crucial future work in real-world deployments of unlearning methods.

Although we have examined FU’s performance on two common clinical DNNs, MLP and LSTM, its scalability to larger, more complex model architectures deserves deeper investigation. To this end, we have performed additional evaluations on a 2-layer Transformer model^50^ with 2.27M learnable parameters (detailed architecture outlined in Supplementary Table 5) and compared its performance with the adopted LSTM network (20K parameters) on the eICU dataset for both mortality and shock prediction tasks. The results (see Supplementary Tables 9 and 10) demonstrate that our FU method can also achieve superior overall performance on Transformer, the core architecture of modern large language models (LLMs) and foundation models. Applying our FU to medical foundation models remains an uncharted and interesting future work, as it could offer prospects to unveil novel insights into the fairness and forgetfulness behaviours of AI models residing on the other side of the scaling law. Last but not least, the datasets used in this study are collected from two countries, the UK and the US, and it will be interesting to discover how well our conclusions will generalise to countries of different regions, e.g., Asian, African, etc.

Overall, the algorithmic unfairness arising from forgetfulness is a complicated, challenging, and highly impactful issue associated with the ethical conflicts between RtbF and fairness. Although our FU strategy is still in its infancy and has several drawbacks, it represents the first attempt to solve this RtbF-fairness dilemma. Our work, although with future research, could foster and deliver novel AI-based innovations for intelligent medicine to benefit healthcare stakeholders with a more trustworthy and unbiased privacy protection practice.

## Methods

### Problem definition

Given a training dataset $D={\{({x}_{i},{y}_{i},{a}_{i})\}}_{i=1}^{N}$ consisting of *N* training records, where ${x}_{i}\in {{\mathbb{R}}}^{T\times L}$ represents the *i*-th input sample with temporal dimension *T* and feature dimension *L*, *y*_*i*_ ∈ {0, 1} denotes the corresponding binary class label, and ${a}_{i}\in {{{\mathcal{A}}}}$ represents the demographic attribute that identifies patient subgroups. Here, ${{{\mathcal{A}}}}=\{{a}_{1},{a}_{2},\ldots,{a}_{K}\}$ denotes the set of *K* demographic categories. A parametric model *f* with parameters *θ* is trained on *D* to learn the mapping from inputs to class predictions. This trained model *f*_*θ*_ serves as the starting point for unlearning and is referred to as the “original” model throughout this work.

For our specific clinical applications, the data characteristics vary across datasets. In the tabular EHR datasets (CURIAL and CURIAL-Combined), each sample *x*_*i*_ is a feature vector with *T* = 1 (i.e., ${x}_{i}\in {{\mathbb{R}}}^{L}$), representing static clinical measurements collected at a single time point. For the time-series EHR dataset (eICU and MIMIC-IV), *x*_*i*_ captures sequential clinical observations over multiple time steps. The demographic attributes *a*_*i*_ correspond to patient ethnicity for CURIAL, eICU, and MIMIC-IV datasets, and hospital site origin for CURIAL-Combined, enabling us to evaluate algorithmic fairness across these clinically relevant demographic and institutional dimensions.

#### Unlearning

To implement unlearning, we partition the original training dataset *D* into two disjoint subsets: the *forgetting setD*_*f*_ ⊂ *D* containing records that need to be removed due to privacy requirements (e.g., RtbF requests), and the *remaining setD*_*r*_ = *D*⧹*D*_*f*_ comprising all other training records. The objective of unlearning is to eliminate the influence of *D*_*f*_ from the original model *f*_*θ*_ such that it retains no information about the forgetting set *D*_*f*_, while preserving its predictive performance on *D*_*r*_.

#### Fairness

We adopt the widely used fairness criterion of equalised odds (EO)^51^. For a random sample (*X*, *Y*, *A*) from the data distribution, where *A* denotes the demographic attribute and *Y* denotes the class label, the model *f*_*θ*_ satisfies EO if its predictions $\widehat{Y}$ are independent of the demographic attribute *A* given the class label *Y*. Formally, this requires

1 $$P(\widehat{Y}=1| A=a,Y=y)=P(\widehat{Y}=1\,|\, A={a}^{{\prime} },Y=y),\forall y\in \{0,1\},\forall a,{a}^{{\prime} }\in {{{\mathcal{A}}}}.$$

This condition ensures equal true positive rates across demographic groups when *y* = 1, and equal false positive rates when *y* = 0.

### Proposed fair unlearning framework

To simultaneously achieve effective data removal and preserve algorithmic fairness across patient subgroups, we propose a fair unlearning framework that explicitly optimises for both unlearning effectiveness and EO preservation. Our fair unlearning (FU) framework operates by computing gradients from three distinct loss functions: the standard classification loss for model utility, the forgetting loss for data removal, and the fairness loss for algorithmic equity preservation. The core innovation lies in the gradient orthogonalisation mechanism, which projects the forgetting gradient onto a subspace orthogonal to the utility and fairness gradients. This orthogonal projection ensures that parameter updates for data forgetting do not interfere with the objectives of maintaining clinical performance and algorithmic fairness. The framework then combines these orthogonalised gradients to update model parameters, enabling simultaneous optimisation of all three objectives without mutual interference.

Our FU framework utilises three distinct loss functions that correspond to the performance preservation objective, the unlearning objective, and the equality objective. The first two objectives are formulated using binary cross-entropy as the base prediction loss:

2 $${{{\mathcal{L}}}}({f}_{\theta }(x),y)=\frac{1}{N}{\sum }_{i=1}^{N}-\left[{y}_{i}\log ({f}_{\theta }({x}_{i}))+(1-{y}_{i})\log (1-{f}_{\theta }({x}_{i}))\right]$$

where *f*_*θ*_(*x*_*i*_) represents the model’s predicted probability for input *x*_*i*_.

The performance preservation objective maintains the model’s predictive capability on the remaining data by minimising the expected prediction loss:

3 $${{{{\mathcal{L}}}}}_{r}={{\mathbb{E}}}_{(x,y) \sim {D}_{r}}[{{{\mathcal{L}}}}({f}_{\theta }(x),y)].$$

Conversely, the unlearning objective removes the influence of forgotten samples by maximising the prediction loss on the forgetting set, which is achieved through the negative expected loss:

4 $${{{{\mathcal{L}}}}}_{f}=-{{\mathbb{E}}}_{(x,y) \sim {D}_{f}}[{{{\mathcal{L}}}}({f}_{\theta }(x),y)].$$

The equality objective addresses algorithmic fairness by minimising prediction disparities across demographic subpopulations within the remaining set *D*_*r*_. To accommodate the multi-class nature of demographic attributes ${a}_{i}\in {{{\mathcal{A}}}}$, we formulate a pairwise equality loss that comprehensively evaluates fairness between all demographic group pairs. For this formulation, we define ${{{{\mathcal{G}}}}}_{a,y}^{(r)}=\{i:({x}_{i},{y}_{i},{a}_{i})\in {D}_{r},{a}_{i}=a,{y}_{i}=y\}$ as the index set of samples belonging to demographic group *a* with ground truth label *y*, and denote its cardinality as ${n}_{a,y}^{(r)}=| {{{{\mathcal{G}}}}}_{a,y}^{(r)}|$. Similarly for any demographic group ${a}^{{\prime} }\in {{{\mathcal{A}}}}$, we define ${{{{\mathcal{G}}}}}_{{a}^{{\prime} },y}^{(r)}$ and ${n}_{{a}^{{\prime} },y}^{(r)}$. The equality loss then systematically measures and penalises prediction discrepancies between all pairs of demographic groups conditioned on the same label:

5 $${{{{\mathcal{L}}}}}_{e}={\sum }_{a\ne {a}^{{\prime} }\in {{{\mathcal{A}}}}}{\sum }_{y\in \{0,1\}}\left(\frac{1}{{n}_{a,y}^{(r)}\cdot {n}_{{a}^{{\prime} },y}^{(r)}}{\sum }_{i\in {{{{\mathcal{G}}}}}_{a,y}^{(r)}}{\sum }_{j\in {{{{\mathcal{G}}}}}_{{a}^{{\prime} },y}^{(r)}}{\left({f}_{\theta }({x}_{i})-{f}_{\theta }({x}_{j})\right)}^{2}\right).$$

However, jointly optimising these three objectives is challenging due to conflicting gradient directions. To this end, we employ gradient orthogonalisation to project the forgetting gradient onto a subspace orthogonal to both utility and fairness preservation directions. We first compute the gradients for each objective:

6 $${g}_{r}={\nabla }_{\theta }{{{{\mathcal{L}}}}}_{r},\,{g}_{f}={\nabla }_{\theta }{{{{\mathcal{L}}}}}_{f},\,{g}_{e}={\nabla }_{\theta }{{{{\mathcal{L}}}}}_{e}.$$

We define the subspace ${{{\mathcal{S}}}}={{{\rm{span}}}}\{{g}_{r},{g}_{e}\}$, which encapsulates the parameter update directions essential for preserving both model utility and algorithmic fairness. To enable precise orthogonal projection, we apply Gram-Schmidt orthogonalisation to {*g*_*r*_, *g*_*e*_}, yielding an orthonormal basis ${{{\mathcal{V}}}}=\{{v}_{1},{v}_{2}\}$ for ${{{\mathcal{S}}}}$:

7 $${v}_{1}=\frac{{g}_{r}}{\parallel {g}_{r}\parallel },\,{v}_{2}=\frac{{g}_{e}-\langle {g}_{e},{v}_{1}\rangle {v}_{1}}{\parallel {g}_{e}-\langle {g}_{e},{v}_{1}\rangle {v}_{1}\parallel },$$

here, 〈 ⋅ , ⋅ 〉 denotes the inner product between vectors.

Subsequently, the original forgetting gradient *g*_*f*_ is projected onto the orthogonal complement of ${{{\mathcal{S}}}}$ to obtain the orthogonal component ${g}_{f}^{\perp }$:

8 $${g}_{f}^{\perp }={g}_{f}-{\sum }_{i=1}^{2}\langle {g}_{f},{v}_{i}\rangle {v}_{i}.$$

The orthogonalised forgetting gradient ${g}_{f}^{\perp }$ now operates exclusively in the null space of the utility-fairness subspace. The final parameter update combines all three gradients:

9 $${g}_{FU}=\alpha {g}_{f}^{\perp }+\beta {g}_{r}+\gamma {g}_{e},$$

where *α*, *β*, and *γ* are hyper-parameters that balance the relative importance of forgetting, utility preservation, and fairness enhancement, respectively. Box 1 presents the complete implementation.

Box 1 Fair unlearning framework (FU) **Input:** The original model *f*_*θ*_, the forgetting set *D*_*f*_, the remaining set *D*_*r*_

**Require:** The gradient weights *α*, *β*, *γ*, the learning rate *η*, the number of iterations *T*

1: **for** *t* = 1 to *T* **do**

2: Sample mini-batch *B*_*r*_ ⊂ *D*_*r*_

3: Sample mini-batch *B*_*f*_ ⊂ *D*_*f*_

4: Compute utility preservation loss ${{{{\mathcal{L}}}}}_{r}$ via Eq. (3)

5: Compute forgetting loss ${{{{\mathcal{L}}}}}_{f}$ via Eq. (4)

6: Compute fairness loss ${{{{\mathcal{L}}}}}_{e}$ via Eq. (5)

7: Compute gradients *g*_*r*_, *g*_*f*_, *g*_*e*_ via Eq. (6)

8: Perform gradient orthogonalisation:

9: 7D3Compute orthogonal basis {*v*_1_, *v*_2_} for ${{{\mathcal{S}}}}={{{\rm{span}}}}\{{g}_{r},{g}_{e}\}$ via Eq. (7)

10: 7D3Compute orthogonal component ${g}_{f}^{\perp }$ via Eq. (8)

11: Compute final gradient *g*_*FU*_ via Eq. (9)

12: Update parameters *θ* ← *θ* − *ηg*_*FU*_

13: **end for**

**Output:** The unlearned model *f*_*θ*_

### Incremental forgetting setup

In realistic clinical deployments, patient RtbF requests typically emerge progressively over time rather than arriving as large wholesale batches. This necessitates continuous small-scale unlearning operations rather than infrequent large-scale removals. To reflect this practical scenario, we adopt an incremental forgetting protocol. Specifically, starting from the original model, we remove patient records in consecutive 1% increments, where each subsequent unlearning step operates on the unlearned model resulting from the previous forgetting operation. In this work, we conduct comprehensive experiments spanning 1% to 10% cumulative forgetting ratios, presenting results at three representative forgetting sizes: 1%, 5%, and 10%.

### Hyper-parameters

For the proposed fair unlearning framework, the key hyperparameters include the gradient weighting coefficients *α*, *β*, and *γ* (see Eq. (9)), which are selected from the range {0.1, 1.0}. The learning rate *η* is set as {1*e* − 4, 5*e* − 3, 8*e* − 4, 5*e* − 4, 3*e* − 3} for CURIAL, CURIAL-Combined, eICU (mortality and shock prediction), and MIMIC-IV, respectively. For all experiments, we use the Adam optimiser with default momentum parameters (*β*_1_ = 0.9, *β*_2_ = 0.999). The batch size is set to 256 for all datasets.

The original models are trained using standard binary cross-entropy loss for 30 epochs. For the baseline unlearning methods, learning rates are selected from the same range, with the criterion that the unlearned models maintain AUROC performance above 0.7, while other method-specific parameters are optimised according to their respective literature recommendations. We thoroughly describe the hyper-parameter settings in Supplementary Table 6 and investigate the effects of fairness gradient weights *γ* in Supplementary Table 7.

To ensure statistical robustness, each experiment was repeated 5 times with different random seeds, and results are reported as “mean (SD)”.

### Evaluation metrics

We detail the specific implementation procedures for the key evaluation metrics used in our experiments.

To evaluate the unlearning effectiveness, we use two types of privacy attacks, membership inference attack (MIA) and model inversion (MI), on the forgetting records. MIA assesses whether the forgetting set records can be distinguished from unseen data by the unlearned model. Specifically, we concatenate the forgetting records with an equal number of randomly selected records from the test set to create a balanced evaluation dataset. We then train a logistic regression classifier to predict membership (whether a record belongs to the forgetting set or test set) based on the model’s prediction confidence scores and loss values on these records. MI reconstructs patient records corresponding to target labels and then quantifies how closely these reconstructed records resemble the forgetting records. Specifically, starting from random noise, we iteratively optimise the reconstructed records with gradient descent to make the model’s predictions match the target labels, using binary cross-entropy loss. After convergence, we select the best reconstructed records and compute the average L2 distance to their k-nearest neighbour forgetting records with the same label, denoted as MI-KnnDist, and we set K to 10 across all experiments. A smaller MI-KnnDist indicates that the reconstructed record is closer to the forgetting records, implying greater residual memorisation of the records that should have been forgotten. Note that for the CURIAL dataset, since the forgetting records are sampled from the training records (OUH trust cohort), we restrict MIA and MI evaluations to the OUH prospective evaluation set to avoid confounding factors from inter-hospital data distribution differences, ensuring that the membership inference attack and model inversion attack purely assess unlearning effectiveness.

To assess algorithmic fairness, we adopt two standard metrics, equalised odds (EO) and demographic parity (DP). EO computes the standard deviation of true positive rates and false positive rates across demographic groups^18^, denoted as EO-TP and EO-FP, respectively. DP measures the standard deviation of positive prediction rates across demographic groups, complementing EO by focusing on selection-rate disparities independent of ground-truth labels. Lower standard deviation values of EO-TP, EO-FP and DP indicate better fairness across subgroups. The calculations for EO-TP, EO-FP, and DP are formulated as follows:

10 $$\begin{array}{rcl}{\mathsf{EO}}\,-\,{\mathsf{TP}} &=& SD\left(\left\{P(\widehat{Y}=1| A={a}_{1},Y=1),P(\widehat{Y}=1| A={a}_{2},Y=1),\right.\right.\\ & & \left.\left.\ldots,P(\widehat{Y}=1| A={a}_{K},Y=1)\right\}\right)\\ &=& SD\left(\left\{\frac{{TP}_{1}}{{TP}_{1}+{FN}_{1}},\frac{{TP}_{2}}{{TP}_{2}+{FN}_{2}},\ldots,\frac{{TP}_{K}}{{TP}_{K}+{FN}_{K}}\right\}\right),\end{array}$$

11 $$\begin{array}{rcl}{\mathsf{EO}}\,-\,{\mathsf{FP}} &=& SD\left(\left\{P(\widehat{Y}=1| A={a}_{1},Y=0),P(\widehat{Y}=1| A={a}_{2},Y=0),\right.\right.\\ & & \left.\left.\ldots,P(\widehat{Y}=1| A={a}_{K},Y=0)\right\}\right)\\ &=& SD\left(\left\{\frac{{FP}_{1}}{{FP}_{1}+{TN}_{1}},\frac{{FP}_{2}}{{FP}_{2}+{TN}_{2}},\ldots,\frac{{FP}_{K}}{{FP}_{K}+{TN}_{K}}\right\}\right),\end{array}$$

12 $$\begin{array}{rcl}{\mathsf{DP}} &=& SD\left(\left\{P(\widehat{Y}=1| A={a}_{1}),P(\widehat{Y}=1| A={a}_{2}),\right.\right.\\ & & \left.\left.\ldots,P(\widehat{Y}=1| A={a}_{K})\right\}\right)\\ &=& SD\left(\left\{\frac{{TP}_{1}+{FP}_{1}}{{TP}_{1}+{FP}_{1}+{TN}_{1}+{FN}_{1}},\frac{{TP}_{2}+{FP}_{2}}{{TP}_{2}+{FP}_{2}+{TN}_{2}+{FN}_{2}},\right.\right.\\ & & \left.\left.\ldots,\frac{{TP}_{K}+{FP}_{K}}{{TP}_{K}+{FP}_{K}+{TN}_{K}+{FN}_{K}}\right\}\right),\end{array}$$

where *K* is the number of demographic subgroups in ${{{\mathcal{A}}}}$, and *TP*_*j*_, *FN*_*j*_, *FP*_*j*_, *TN*_*j*_ are the empirically computed true positives, false negatives, false positives, and true negatives for the demographic subgroup *A* = *a*_*j*_ (for *j* = 1, 2, …, *K*), respectively, and *SD*(⋅) denotes the standard deviation function.

### Radar plot scores

To evaluate the comprehensive performance, we construct radar plots with six radii representing different evaluation dimensions, as shown in Fig. 3b. We aggregate performance across the 1%, 5%, and 10% forgetting ratios, calculating the overall performance for each method to provide a holistic evaluation across different forgetting scenarios.

The six axes in the radar plot are computed as follows and converted into the percentage system for better visualisations:

- The scores of the four fairness radii, i.e., “CURIAL”, “CURIAL-Combined”, “eICU-Mortality”, “eICU-Shock”, and “MIMIC-IV”, describe the overall algorithmic fairness on a corresponding dataset and task. Each is computed by averaging the EO-TP, EO-FP, and DP values for the respective dataset and task, then applying exponential transformation $\exp (-\overline{EO}\times s)$, where $\overline{EO}$ is the averaged fairness score and *s* is the dataset-specific scale factor (3 for all datasets).
- The clinical utility score represents the average AUROC performance across all four datasets for each unlearning method.
- The unlearning effectiveness (Membership Inference) axis is computed by averaging MIA-AUROC values across all datasets for each method, then applying transformation $\exp (-| \overline{MIA}-0.5| )$ where $\overline{MIA}$ denotes the averaged MIA-AUROC. The value reflects that MIA-AUROC values closer to 0.5 indicate better forgetting capability.
- The unlearning effectiveness (Model Inversion) axis score is computed as the ratio between MI-KnnDist computed under the unlearned model and that under the retrained model, with this ratio clipped to lie between 0 and 1, such that higher scores indicate better forgetting performance.

All the converted scores are the higher, the better.

### Ethical approval

Approval to use de-identified, routinely collected clinical and microbiology data from electronic health records for the development and validation of artificial intelligence models to detect COVID-19 was granted by the NHS Health Research Authority (CURIAL; NHS HRA IRAS ID: 281832).

### Reporting summary

Further information on research design is available in the Nature Portfolio Reporting Summary linked to this article.

## Supplementary information

Supplementary Information

Reporting Summary

Transparent Peer Review file

## Source data

Source Data

## Data Availability

Data from OUH studied here are available from the Infections in Oxfordshire Research Database (https://oxfordbrc.nihr.ac.uk/research-themes/modernising-medical-microbiology-and-big-infection-diagnostics/iord-about/), subject to an application meeting the ethical and governance requirements of the database. Data from UHB, PUH, and BH are available at reasonable request from the respective trusts, subject to HRA requirements. The eICU Collaborative Research Database is available online at https://www.physionet.org/content/eicu-crd/2.0/. The MIMIC-IV dataset is available online at https://physionet.org/content/mimiciv/2.0/. Source data are provided in this paper.

## Code availability

The code repository can be found at: https://github.com/cyxanna/Mitigating-algorithmic-unfairness-from-forgetfulness-Nature-Communications. The code is also archived on Zenodo at 10.5281/zenodo.18830934.
