# Supplementary material for: Mitigating algorithmic unfairness arising from forgetfulness of medical records in clinical artificial intelligence
Source: Nat Commun. 2026 May 4;17:6009. doi: 10.1038/s41467-026-72601-7 (PMC13346932; doi:10.1038/s41467-026-72601-7)
Supplement: Supplementary file 1 — Supplementary Information [file 41467_2026_72601_MOESM1_ESM.pdf]

Supplementary Information - Mitigating algorithmic unfairness arising from forgetfulness of medical records in clinical artificial intelligence

Supplementary Note 1 - Datasets

CURIAL data and preprocessing

Supplementary Table 1: Clinical variables used on CURIAL and CURIAL-Combined for COVID-19 screening task.

**Vital Signs**<br>Heart Rate, Respiratory Rate, Systolic Blood Pressure, Diastolic Blood Pressure, Temperature Tympanic, Oxygen Saturation, Delivery device used
**Blood Tests**<br>Haemoglobin, Haematocrit, Mean Cell Volume, White Cell Count, Neutrophil Count, Lymphocyte Count, Monocyte Count, Eosinophil Count, Basophil Count, Platelets
**Liver Function Tests & C-reactive protein**<br>Albumin, Alkaline Phosphatase, Alanine Aminotransferase, Bilirubin, C-reactive Protein
**Urea & Electrolytes**<br>Sodium, Potassium, Creatinine, Urea, Estimated Glomerular Filtration Rate

The CURIAL database is an anonymised database with United Kingdom National Health Service (NHS) approval via the Health Research Authority (HRA) (CURIAL; NHS HRA IRAS ID: 281832). This dataset contains four NHS trusts:

- Oxford University Hospitals NHS Foundation Trust (OUH trust) includes all patients presenting to acute and emergency departments who receive routine blood tests on arrival. The dataset defines two cohorts based on temporal presentation: pre-pandemic cases (before December 1, 2019) constitute the COVID-19-negative control group, whilst cases from the first wave of the UK pandemic (December 1, 2019 to June 30, 2020) with PCR-confirmed SARS-CoV-2 infection form the COVID-19-positive group. Patients are excluded if they decline electronic health record research participation, lack laboratory blood tests, or are under 18 years of age. Due to limited testing availability and imperfect PCR sensitivity during early pandemic phases, we select the pre-pandemic cohort as controls to ensure reliable negative classification.

- Portsmouth Hospitals University NHS Foundation Trust (PUH trust) includes all patients admitted to Queen Alexandria Hospital between March 1, 2020 and February 28, 2021. COVID-19 positivity is confirmed through laboratory RT-PCR testing, with any positive result within 48 hours of admission classified as a true positive case.

- University Hospitals Birmingham NHS Foundation Trust (UHB trust) includes all patients admitted to The Queen Elizabeth Hospital, Birmingham between December 1, 2019 and October 29, 2020. COVID-19 positivity is confirmed through laboratory-based SARS-CoV-2 RT-PCR testing.

- Bedfordshire NHS Foundation Trust (BH trust) includes all patients admitted to Bedford Hospital between January 1, 2021 and March 31, 2021. COVID-19 positivity is confirmed through point-of-care PCR-based nucleic acid testing performed on admission [SAMBA-II & Panther Fusion System, Diagnostics in the Real World, UK, and Hologic, USA].

Supplementary Table 1 details the clinical variables extracted from patient presentations across all four NHS trusts for COVID-19 screening task. The feature vector for each patient is a 27-dimensional vector.

CURIAL-Combined data and preprocessing

Supplementary Table 2: Summary population characteristics for training and test set of CURIAL-Combined.

	Training	Test	Total
n, patients	175,013	40,639	215,652
n, positive	855 (0.5%)	4,369 (10.8%)	4,576 (2.1%)
Hospital:			
OUH(%)	86,462 (49.4%)	18,499 (45.5%)	104,961 (48.7%)
PUH(%)	10,873 (6.2%)	2,719 (6.7%)	13,592 (6.3%)
UHB(%)	76,188 (43.5%)	19,048 (46.9%)	95,236 (44.2%)
BH(%)	1,490 (0.9%)	373 (0.9%)	1,863 (0.9%)

The CURIAL-Combined dataset is a combination of the four NHS trusts. Supplementary Table 2 provides the summary population characteristics for training and test sets of CURIAL-Combined.

eICU data and preprocessing

Supplementary Table 3: Clinical variables for the mortality and shock prediction tasks in eICU.

**Category**	**Variables**	**Data Type**
Vital Signs	Heart rate, Mean arterial pressure, Diastolic blood pressure, Systolic blood pressure, O2, Respiratory rate, Temperature, Glucose, FiO2, pH	Numerical
Patient Demographics	Height, Weight, Age	Numerical
Patient Characteristics	Ethnicity, Gender, Admission diagnosis	Categorical
Clinical Assessment	Glasgow Coma Score Total, Glasgow Coma Score Eyes, Glasgow Coma Score Motor, Glasgow Coma Score Verbal	Categorical

On eICU dataset, the selected cohort consists of patients aged 18 years or older with intensive care unit admissions, each possessing a minimum of 15 records to ensure sufficient temporal information for analysis. We evaluate two clinical prediction tasks using the processed eICU data:

- In-hospital mortality prediction: This binary classification task aims to predict patient outcomes at hospital discharge. The cohort for this task includes patients with available hospital discharge status and a length of stay of at least 48 hours, with our analysis focusing on predictions during the first 48 hours of admission.
- Shock prediction: This task involves predicting the occurrence of shock at 4-hour intervals, resulting in time-series data with 4 time-steps for each patient.

For data preprocessing, we group patient records into 1-hour windows and impute missing values using the mean value within each window, selecting the last valid record for each specific window. The clinical variables used in our analysis are detailed in Supplementary Table 3, encompassing both categorical and numerical variables from vital signs, patient demographics, patient characteristics, and clinical assessment. For numerical variables, we standardize the values to have zero mean and unit variance. For categorical variables, we apply one-hot encoding, resulting in a 442-dimensional feature vector for each time step across both prediction tasks that are listed below:

1. Admission diagnosis: Rhythm disturbance (atrial, supraventricular)
2. Admission diagnosis: Sepsis, renal/UTI (including bladder)
3. Admission diagnosis: Sepsis, pulmonary
4. Admission diagnosis: Arrest, respiratory (without cardiac arrest)
5. Admission diagnosis: Overdose, sedatives, hypnotics, antipsychotics, benzodiazepines
6. Admission diagnosis: CHF, congestive heart failure
7. Admission diagnosis: Mitral valve replacement
8. Admission diagnosis: Graft, femoral-popliteal bypass
9. Admission diagnosis: Asthma
10. Admission diagnosis: Head only trauma
11. Admission diagnosis: CVA, cerebrovascular accident/stroke
12. Admission diagnosis: Anemia
13. Admission diagnosis: Pneumothorax
14. Admission diagnosis: Hypovolemia (including dehydration, Do not include shock states)
15. Admission diagnosis: Aneurysm, dissecting aortic
16. Admission diagnosis: Aneurysm, abdominal aortic; with rupture
17. Admission diagnosis: Infarction, acute myocardial (MI)
18. Admission diagnosis: Thoracotomy for other reasons
19. Admission diagnosis: Transphenoidal surgery
20. Admission diagnosis: Cholecystectomy or cholangitis, surgery for (gallbladder removal)
21. Admission diagnosis: Respiratory surgery, other
22. Admission diagnosis: Endarterectomy, carotid
23. Admission diagnosis: Thrombosis, vascular (deep vein)
24. Admission diagnosis: Coma/change in level of consciousness (for hepatic see GI, for diabetic see Endocrine, if related to cardiac arrest, see CV)
25. Admission diagnosis: Pulmonary valve surgery
26. Admission diagnosis: Biopsy, brain
27. Admission diagnosis: Obstruction/other, surgery for (with or without ileal conduit)
28. Admission diagnosis: Sepsis, unknown
29. Admission diagnosis: Bleeding, GI-location unknown
30. Admission diagnosis: Aneurysm, abdominal aortic
31. Admission diagnosis: Extremity only trauma, surgery for
32. Admission diagnosis: Emphysema/bronchitis
33. Admission diagnosis: Thoracotomy for lung cancer
34. Admission diagnosis: Overdose, alcohols (bethanol, methanol, ethylene glycol)
35. Admission diagnosis: Bleeding, upper GI
36. Admission diagnosis: Hypertension, uncontrolled (for cerebrovascular accident-see Neurological System)
37. Admission diagnosis: Shunts and revisions
38. Admission diagnosis: Pneumonia, bacterial
39. Admission diagnosis: Head/extremity trauma
40. Admission diagnosis: Seizures (primary-no structural brain disease)
41. Admission diagnosis: MI admitted > 24 hrs after onset of ischemia
42. Admission diagnosis: Laminectomy/spinal cord decompression (excluding malignancies)
43. Admission diagnosis: Hematoma subdural, surgery for
44. Admission diagnosis: Pneumonia, other
45. Admission diagnosis: Cardiac arrest (with or without respiratory arrest; for respiratory arrest see Respiratory System)
46. Admission diagnosis: Bleeding, lower GI
47. Admission diagnosis: CABG alone, coronary artery bypass grafting
48. Admission diagnosis: Efffusion, pericardial
49. Admission diagnosis: Aneurysm, thoracic aortic
50. Admission diagnosis: Vascular surgery, other
51. Admission diagnosis: Embolus, pulmonary
52. Admission diagnosis: Angina, unstable (angina interferes w/quality of life or meds are tolerated poorly)
53. Admission diagnosis: Sepsis, other
54. Admission diagnosis: Bleeding-other GI, surgery for
55. Admission diagnosis: Aortic valve replacement (isolated)
56. Admission diagnosis: Overdose, street drugs (opiates, cocaine, amphetamine)
57. Admission diagnosis: Fusion-spinal/Harrington rods
58. Admission diagnosis: Neoplasm, neurologic
59. Admission diagnosis: Leukemia, acute myelocytic
60. Admission diagnosis: Hematoma, subdural
61. Admission diagnosis: Hemorrhage (for gastrointestinal bleeding GI-see GI system) (for trauma see Trauma)
62. Admission diagnosis: Chest/abdomen trauma
63. Admission diagnosis: Obstruction-airway (i.e., acute epiglottitis, post-extubation edema, foreign body, etc)
64. Admission diagnosis: Subarachnoid hemorrhage or intracranial aneurysm
65. Admission diagnosis: Respiratory - medical, other
66. Admission diagnosis: Cellulitis and localized soft tissue infections, surgery for
67. Admission diagnosis: Knee replacement, total (non-traumatic)
68. Admission diagnosis: Bleeding-lower GI, surgery for
69. Admission diagnosis: GI obstruction
70. Admission diagnosis: Complications of prev. peripheral vasc. surgery,surgery for (i.e.ligation of bleeder, exploration and evacuation of hematoma, debridement, pseudoaneurysms, clots, fistula, etc.)
71. Admission diagnosis: Hematomas
72. Admission diagnosis: Rhythm disturbance (ventricular)
73. Admission diagnosis: Alcohol withdrawal
74. Admission diagnosis: Cardiomyopathy
75. Admission diagnosis: Neurologic surgery, other
76. Admission diagnosis: Effusions, pleural
77. Admission diagnosis: GI perforation/rupture, surgery for
78. Admission diagnosis: Diabetic ketoacidosis
79. Admission diagnosis: Neoplasm-cranial, surgery for (excluding transphenoidal)
80. Admission diagnosis: Angina, stable (asymp or stable pattern of symptoms w/meds)
81. Admission diagnosis: Hypoglycemia
82. Admission diagnosis: Drug withdrawal
83. Admission diagnosis: Thoracotomy for esophageal cancer
84. Admission diagnosis: Seizures-intractable, surgery for
85. Admission diagnosis: Abscess, neurologic
86. Admission diagnosis: Acid-base/electrolyte disturbance
87. Admission diagnosis: Neurologic medical, other
88. Admission diagnosis: Heart transplant

89. Admission diagnosis: Rhabdomyolysis
90. Admission diagnosis: Rhythm disturbance (conduction defect)
91. Admission diagnosis: Pancreatitis
92. Admission diagnosis: Ablation or mapping of cardiac conduction pathway
93. Admission diagnosis: Renal failure, acute
94. Admission diagnosis: CABG redo with other operation
95. Admission diagnosis: CABG, minimally invasive; mid-CABG
96. Admission diagnosis: Aortic and Mitral valve replacement
97. Admission diagnosis: Skin surgery, other
98. Admission diagnosis: Anaphylaxis
99. Admission diagnosis: Encephalopathy, hepatic
100. Admission diagnosis: Cerebrospinal fluid leak, surgery for
101. Admission diagnosis: Cancer-colon/rectal, surgery for (including abdominoperineal resections)
102. Admission diagnosis: Thoracotomy for other malignancy in chest
103. Admission diagnosis: Hip replacement, total (non-traumatic)
104. Admission diagnosis: GI vascular ischemia, surgery for (resection)
105. Admission diagnosis: Pneumonia, aspiration
106. Admission diagnosis: Thrombus, arterial
107. Admission diagnosis: Cranioplasty and complications from previous craniotomies
108. Admission diagnosis: Cancer-other GI tract, surgery for (i.e., hepatoma, gallbladder etc.)
109. Admission diagnosis: CABG alone, redo
110. Admission diagnosis: Overdose, antidepressants (cyclic, lithium)
111. Admission diagnosis: Hemorrhage/hematoma, intracranial
112. Admission diagnosis: Genitourinary surgery, other
113. Admission diagnosis: Sepsis, GI
114. Admission diagnosis: Overdose, analgesic (aspirin, acetaminophen)
115. Admission diagnosis: Cancer-laryngeal/tracheal, surgery for
116. Admission diagnosis: Hemorrhage/hemoptysis, pulmonary
117. Admission diagnosis: Abdomen/multiple trauma
118. Admission diagnosis: Pneumonia, viral
119. Admission diagnosis: Aneurysm/pseudoaneurysm, other
120. Admission diagnosis: CABG with aortic valve replacement
121. Admission diagnosis: Arteriovenous malformation, surgery for
122. Admission diagnosis: Overdose, other toxin, poison or drug
123. Admission diagnosis: Neoplasm-spinal cord, surgery or other related procedures
124. Admission diagnosis: GI perforation/rupture
125. Admission diagnosis: Extremity only trauma
126. Admission diagnosis: Hemorrhage, intra/retroperitoneal
127. Admission diagnosis: Subarachnoid hemorrhage/intracranial aneurysm, surgery for
128. Admission diagnosis: CABG with mitral valve repair
129. Admission diagnosis: Nephrectomy for neoplasm
130. Admission diagnosis: Bleeding, GI from esophageal varices/portal hypertension
131. Admission diagnosis: Abscess/infection-cranial, surgery for
132. Admission diagnosis: Sepsis, cutaneous/soft tissue
133. Admission diagnosis: Herniorrhaphy
134. Admission diagnosis: Restrictive lung disease (i.e., Sarcoidosis, pulmonary fibrosis)
135. Admission diagnosis: Coagulopathy
136. Admission diagnosis: Mitral valve repair
137. Admission diagnosis: Pre-eclampsia/eclampsia
138. Admission diagnosis: Hepatic failure, acute
139. Admission diagnosis: Monitoring, hemodynamic (pre-operative evaluation)
140. Admission diagnosis: Tricuspid valve surgery
141. Admission diagnosis: Amputation (non-traumatic)
142. Admission diagnosis: Complications of previous open-heart surgery, surgery for (i.e. bleeding, infection, mediastinal rewiring,leaking aortic graft etc.)
143. Admission diagnosis: Chest pain, unknown origin
144. Admission diagnosis: Head/face trauma
145. Admission diagnosis: Cancer, other GI
146. Admission diagnosis: Hemorrhage/hematoma-intracranial, surgery for
147. Admission diagnosis: Cancer, oral/sinus, surgery for
148. Admission diagnosis: Complications of previous GI surgery; surgery for (anastomotic leak, bleeding, abscess, infection, dehiscence, etc.)
149. Admission diagnosis: Renal infection/abscess
150. Admission diagnosis: Tracheostomy
151. Admission diagnosis: Congenital Defect Repair (Other)
152. Admission diagnosis: Spinal/face trauma
153. Admission diagnosis: Oophorectomy with or without salpingectomy with or without lymph node dissection
154. Admission diagnosis: Spinal cord surgery, other
155. Admission diagnosis: Tumor removal, intracardiac
156. Admission diagnosis: Diverticular disease, surgery for
157. Admission diagnosis: Atrial Septal Defect (ASD) Repair
158. Admission diagnosis: GI medical, other
159. Admission diagnosis: Hysterectomy for other benign neoplasm/fibroids
160. Admission diagnosis: Cardiovascular surgery, other
161. Admission diagnosis: Fracture-pathological, non-union, non-traumatic, for fractures due to trauma see Trauma
162. Admission diagnosis: Cardiovascular medical, other
163. Admission diagnosis: Face only trauma
164. Admission diagnosis: Pelvis/hip trauma
165. Admission diagnosis: Chest pain, atypical (noncardiac chest pain)
166. Admission diagnosis: Head/chest trauma
167. Admission diagnosis: Aneurysms, repair of other (except ventricular)
168. Admission diagnosis: Fistula/abscess, surgery for (not inflammatory bowel disease)
169. Admission diagnosis: Abdomen only trauma
170. Admission diagnosis: GI obstruction, surgery for (including lysis of adhesions)
171. Admission diagnosis: Whipple-surgery for pancreatic cancer
172. Admission diagnosis: Cellulitis and localized soft tissue infections
173. Admission diagnosis: Vascular medical, other
174. Admission diagnosis: Appendectomy
175. Admission diagnosis: Endarterectomy (other vessels)
176. Admission diagnosis: Embolectomy (with general anesthesia)
177. Admission diagnosis: Thoracotomy for pleural disease
178. Admission diagnosis: Shunt-portosystemic, surgery for
179. Admission diagnosis: Hernia-hiatal, esophageal surgery for
180. Admission diagnosis: Chest thorax only trauma
181. Admission diagnosis: Hysterectomy for cancer with or without lymph node dissection
182. Admission diagnosis: ARDS-adult respiratory distress syndrome, non-cardiogenic pulmonary edema
183. Admission diagnosis: Esophageal surgery, other
184. Admission diagnosis: Complications for previous spinal cord surgery, surgery for
185. Admission diagnosis: Facial surgery (if related to trauma, see Trauma)
186. Admission diagnosis: Diabetic hyperglycemic hyperosmolar nonketotic coma (HHNC)
187. Admission diagnosis: Obstruction due to neoplasm ,surgery for; (with or without ileal-conduit)
188. Admission diagnosis: Cystectomy for neoplasm
189. Admission diagnosis: CABG with pulmonic or tricuspid valve repair or replacement ONLY.
190. Admission diagnosis: Graft, all other bypass (except renal)
191. Admission diagnosis: Thoracotomy for thoracic/respiratory infection
192. Admission diagnosis: Cesarean section
193. Admission diagnosis: Stereotactic procedure
194. Admission diagnosis: Hepato-renal syndrome
195. Admission diagnosis: Aneurysm, thoracic aortic; with dissection
196. Admission diagnosis: Burr hole placement
197. Admission diagnosis: Atelectasis
198. Admission diagnosis: Pericardial effusion/tamponade
199. Admission diagnosis: Obstruction due to nephrolithiasis, surgery for (with or without ileal-conduit)
200. Admission diagnosis: Cancer-esophageal, surgery for (abdominal approach)
201. Admission diagnosis: Head/multiple trauma
202. Admission diagnosis: CABG redo with valve repair/replacement
203. Admission diagnosis: CABG with mitral valve replacement
204. Admission diagnosis: Defibrillator, automatic implantable cardiac; insertion of
205. Admission diagnosis: CABG with other operation
206. Admission diagnosis: Endocarditis
207. Admission diagnosis: Leukemia, other
208. Admission diagnosis: Obesity-morbid, surgery for
209. Admission diagnosis: Encephalopathies (excluding hepatic)
210. Admission diagnosis: GI surgery, other
211. Admission diagnosis: Cancer, oral
212. Admission diagnosis: Chest/multiple trauma
213. Admission diagnosis: Shock, cardiogenic
214. Admission diagnosis: Orthopedic surgery, other
215. Admission diagnosis: Cosmetic surgery (all)
216. Admission diagnosis: Aneurysm, abdominal aortic; with dissection
217. Admission diagnosis: Poisoning, carbon monoxide, arsenic, cyanide
218. Admission diagnosis: Spinal cord only trauma
219. Admission diagnosis: Graft, femoral-femoral bypass

220. Admission diagnosis: Cancer, tracheal
221. Admission diagnosis: Liver transplant
222. Admission diagnosis: Grafting, skin (all)
223. Admission diagnosis: Cancer-small intestinal, surgery for
224. Admission diagnosis: Encephalitis
225. Admission diagnosis: Thoracotomy for lung reduction
226. Admission diagnosis: Thrombectomy (without general anesthesia)
227. Admission diagnosis: Adrenalectomy
228. Admission diagnosis: Chest/extremity trauma
229. Admission diagnosis: Kidney transplant
230. Admission diagnosis: Nephrectomy (other reasons)
231. Admission diagnosis: Cranial nerve, decompression/ligation
232. Admission diagnosis: Inflammatory bowel disease
233. Admission diagnosis: Mastectomy (all)
234. Admission diagnosis: Leukemia, acute lymphocytic
235. Admission diagnosis: Abdomen/spinal trauma
236. Admission diagnosis: Bladder repair of perforation/rupture
237. Admission diagnosis: Pelvis/extremity trauma
238. Admission diagnosis: Musculoskeletal medical, other
239. Admission diagnosis: Cancer, lung
240. Admission diagnosis: Subarachnoid hemorrhage/arteriovenous malformation
241. Admission diagnosis: Ventricular Septal Defect (VSD) Repair
242. Admission diagnosis: Thrombectomy (with general anesthesia)
243. Admission diagnosis: Graft, all renal bypass
244. Admission diagnosis: Graft, aorto-femoral bypass
245. Admission diagnosis: CABG with double valve repair/replacement
246. Admission diagnosis: Biopsy, open lung
247. Admission diagnosis: GI vascular insufficiency
248. Admission diagnosis: Hypertension-pulmonary, primary/idiopathic
249. Admission diagnosis: Myasthenia gravis
250. Admission diagnosis: Guillain-Barre syndrome
251. Admission diagnosis: Cancer, colon/rectal
252. Admission diagnosis: Prostatectomy, suprapubic; for cancer
253. Admission diagnosis: Renal neoplasm, cancer
254. Admission diagnosis: Cystectomy, other reasons
255. Admission diagnosis: Dilatation (without general anesthesia)
256. Admission diagnosis: Face/multiple trauma
257. Admission diagnosis: Thrombocytopenia
258. Admission diagnosis: Spinal/multiple trauma
259. Admission diagnosis: Arthritis, septic
260. Admission diagnosis: Sickle cell crisis
261. Admission diagnosis: Tamponade, pericardial
262. Admission diagnosis: Meningitis
263. Admission diagnosis: Devices for spine fracture/dislocation
264. Admission diagnosis: Head/spinal trauma
265. Admission diagnosis: Extremity/face trauma
266. Admission diagnosis: Extremity/multiple trauma, surgery for
267. Admission diagnosis: Complications of previous open heart surgery (i.e. bleeding, infection etc.)
268. Admission diagnosis: Hematoma-epidural, surgery for
269. Admission diagnosis: Addisons disease
270. Admission diagnosis: Bleeding-upper GI, surgery for
271. Admission diagnosis: Neuromuscular medical, other
272. Admission diagnosis: Pelvis/face trauma
273. Admission diagnosis: Face/multiple trauma, surgery for
274. Admission diagnosis: Sepsis, gynecologic
275. Admission diagnosis: Chest pain, epigastric
276. Admission diagnosis: Pancreatitis, surgery for
277. Admission diagnosis: Hydrocephalus, obstructive
278. Admission diagnosis: Cancer, pancreatic
279. Admission diagnosis: Hemothorax
280. Admission diagnosis: Cancer, esophageal
281. Admission diagnosis: Weaning from mechanical ventilation (transfer from other unit or hospital only)
282. Admission diagnosis: Pericarditis
283. Admission diagnosis: Hypothermia
284. Admission diagnosis: Smoke inhalation
285. Admission diagnosis: Bleeding-variceal, surgery for (excluding vascular shunting-see surgery for portosystemic shunt)
286. Admission diagnosis: Graft, removal of infected vascular
287. Admission diagnosis: Chest/spinal trauma
288. Admission diagnosis: Cancer, laryngeal
289. Admission diagnosis: Graft for dialysis, insertion of
290. Admission diagnosis: Renal bleeding
291. Admission diagnosis: Head/pelvis trauma
292. Admission diagnosis: Pelvis/multiple trauma
293. Admission diagnosis: Thyroidectomy
294. Admission diagnosis: Apnea, sleep
295. Admission diagnosis: Genitourinary medical, other
296. Admission diagnosis: Renal obstruction
297. Admission diagnosis: Cholangitis
298. Admission diagnosis: Transplant, other
299. Admission diagnosis: Splenectomy
300. Admission diagnosis: Lymphoma, non-Hodgkins
301. Admission diagnosis: Cyst, rupture ovarian
302. Admission diagnosis: Chest pain, respiratory
303. Admission diagnosis: Exenteration, pelvic-female
304. Admission diagnosis: Ectopic pregnancy (all)
305. Admission diagnosis: Spinal/extremity trauma
306. Admission diagnosis: GI Abscess/cyst-primary, surgery for
307. Admission diagnosis: Metabolic/endocrine medical, other
308. Admission diagnosis: Thoracotomy for bronchopleural fistula
309. Admission diagnosis: Kidney-pancreas transplant
310. Admission diagnosis: Hyperthyroid storm/crisis
311. Admission diagnosis: Thoracotomy for benign tumor (i.e. mediastinal chest wall mass, thymectomy)
312. Admission diagnosis: Amyotrophic lateral sclerosis
313. Admission diagnosis: Pericardiectomy (total/subtotal)
314. Admission diagnosis: Graft, aorto-iliac bypass
315. Admission diagnosis: Hematologic medical, other
316. Admission diagnosis: Peritonitis
317. Admission diagnosis: Ventriculostomy
318. Admission diagnosis: Toxicity, drug (i.e., beta blockers, calcium channel blockers, etc.)
319. Admission diagnosis: Inflammatory bowel disease, surgery for
320. Admission diagnosis: Pancytopenia
321. Admission diagnosis: Gastrostomy
322. Admission diagnosis: Hypothyroid/myxedema
323. Admission diagnosis: Chest/face trauma
324. Admission diagnosis: Infection/abscess, other surgery for
325. Admission diagnosis: Contusion, myocardial (include r/o)
326. Admission diagnosis: Abdomen/pelvis trauma
327. Admission diagnosis: Prostatectomy, suprapubic; for benign prostatic hypertrophy
328. Admission diagnosis: Metabolic/endocrine surgery, other
329. Admission diagnosis: Abdomen/extremity trauma
330. Admission diagnosis: Extremity/multiple trauma
331. Admission diagnosis: Head/abdomen trauma
332. Admission diagnosis: Chest/pelvis trauma
333. Admission diagnosis: Chest/thorax only trauma
334. Admission diagnosis: Hematologic surgery, other
335. Admission diagnosis: Trauma surgery, other
336. Admission diagnosis: Blood transfusion reaction
337. Admission diagnosis: Cancer-stomach, surgery for
338. Admission diagnosis: Trauma medical, other
339. Admission diagnosis: Heat exhaustion/stroke
340. Admission diagnosis: GI abscess/cyst
341. Admission diagnosis: Neutropenia
342. Admission diagnosis: Pelvis/spinal trauma
343. Admission diagnosis: Face only trauma, surgery for
344. Admission diagnosis: Embolectomy (without general anesthesia)
345. Admission diagnosis: Vena cava filter insertion
346. Admission diagnosis: Chest pain, musculoskeletal
347. Admission diagnosis: Peritonitis, surgery for
348. Admission diagnosis: Diverticular disease
349. Admission diagnosis: Burn
350. Admission diagnosis: Thyroidectomy and Parathyroidectomy
351. Admission diagnosis: Hemorrhage, postpartum
352. Admission diagnosis: Leukemia, chronic myelocytic
353. Admission diagnosis: Near drowning accident
354. Admission diagnosis: TURP, transurethral prostate resection for benign prostatic hypertrophy
355. Admission diagnosis: Apnea-sleep; surgery for (i.e., UPPP - uvulopalatopharyngoplasty)
356. Admission diagnosis: Hematoma, epidural
357. Admission diagnosis: TURP, transurethral prostate resection for cancer
358. Admission diagnosis: Cancer, stomach
359. Admission diagnosis: Pneumonia, parasitic (i.e., Pneumocystic pneumonia)
360. Admission diagnosis: Pneumonia, fungal
361. Admission diagnosis: Lymphoma, Hodgkins
362. Admission diagnosis: Nontraumatic coma due to anoxia/ischemia
363. Admission diagnosis: Parathyroidectomy
364. Admission diagnosis: Pelvic relaxation (cystocele, rectocele, etc.)
365. Admission diagnosis: Extremity/face trauma, surgery for
366. Admission diagnosis: CAPD catheter insertion
367. Admission diagnosis: Adrenal neoplasm (including pheochromocytoma)

368. Admission diagnosis: Dilatation (with general anesthesia)
369. Admission diagnosis: Aneurysm, thoracic aortic; with rupture
370. Admission diagnosis: Lupus, systemic
371. Admission diagnosis: Hyperthermia
372. Admission diagnosis: Vena cava clipping
373. Admission diagnosis: Lymph node dissection, pelvic or retroperitoneal (male)
374. Admission diagnosis: Bullectomy
375. Admission diagnosis: Peritoneal lavage
376. Admission diagnosis: Thyroid neoplasm
377. Admission diagnosis: Connective tissue disease (mixed)
378. Admission diagnosis: Palsy, cranial nerve
379. Admission diagnosis: Anastomosis, vascular
380. Admission diagnosis: Lung transplant, bilateral
381. Admission diagnosis: Ulcer disease, peptic
382. Admission diagnosis: Papillary muscle rupture
383. Admission diagnosis: Aneurysm repair, ventricular
384. Admission diagnosis: Lung transplant, single
385. Admission diagnosis: Lymphoma, non-Hodgkins, surgery for (including staging)
386. Admission diagnosis: Lymph node dissection, pelvic or retroperitoneal (female)
387. Admission diagnosis: Abdomen/face trauma
388. Admission diagnosis: Leukemia, chronic lymphocytic
389. Admission diagnosis: Vasculitis
390. Admission diagnosis: Arthritis, rheumatoid
391. Admission diagnosis: Shunt, peritoneal-venous; surgery for
392. Admission diagnosis: Myositis, viral
393. Gender: Female
394. Gender: Male
395. Ethnicity: Caucasian
396. Ethnicity: African American
397. Ethnicity: Hispanic
398. Ethnicity: Asian
399. Ethnicity: Native American
400. Ethnicity: Other/Unknown
401. Glasgow Coma Score Total: 3
402. Glasgow Coma Score Total: 4
403. Glasgow Coma Score Total: 5
404. Glasgow Coma Score Total: 6
405. Glasgow Coma Score Total: 7
406. Glasgow Coma Score Total: 8
407. Glasgow Coma Score Total: 9
408. Glasgow Coma Score Total: 10
409. Glasgow Coma Score Total: 11
410. Glasgow Coma Score Total: 12
411. Glasgow Coma Score Total: 13
412. Glasgow Coma Score Total: 14
413. Glasgow Coma Score Total: 15
414. Glasgow Coma Score Total: 16
415. Glasgow Coma Score Eyes: 2
416. Glasgow Coma Score Eyes: 3
417. Glasgow Coma Score Eyes: 4
418. Glasgow Coma Score Eyes: 5
419. Glasgow Coma Score Motor: 1
420. Glasgow Coma Score Motor: 2
421. Glasgow Coma Score Motor: 3
422. Glasgow Coma Score Motor: 4
423. Glasgow Coma Score Motor: 5
424. Glasgow Coma Score Motor: 6
425. Glasgow Coma Score Verbal: 1
426. Glasgow Coma Score Verbal: 2
427. Glasgow Coma Score Verbal: 3
428. Glasgow Coma Score Verbal: 4
429. Glasgow Coma Score Verbal: 5
430. Admissionheight
431. Admissionweight
432. Age
433. Heart Rate
434. MAP (mmHg)
435. Invasive BP Diastolic
436. Invasive BP Systolic
437. O2 Saturation
438. Respiratory Rate
439. Temperature (C)
440. Glucose
441. FiO2
442. pH

We use a 80:20 split ratio for training and test set on both mortality prediction and shock prediction tasks. Supplementary Table 4 provides the summary population characteristics for training and test sets of eICU for mortality prediction and shock prediction tasks, respectively.

Supplementary Table 4: Summary population characteristics for training and test set of eICU.

Mortality prediction task

	Training	Test	Total
n, total	24,423	6,106	30,529
Ethnicity:			
Caucasian (%)	18,905 (77.4)	4,750 (77.8)	23,655 (82.9)
African American (%)	2,735 (11.2)	667 (10.9)	3,402 (12.0)
Hispanic (%)	881 (3.6)	230 (3.8)	1,111 (3.9)
Asian (%)	399 (1.6)	93 (1.5)	492 (1.7)
Native American (%)	-	-	-
Other/Unknown (%)	1,503 (6.2)	366 (6.0)	1,869 (6.6)

Shock prediction task

	Training	Test	Total
n, total	38,630	9,658	48,288
Ethnicity:			
Caucasian (%)	29,421 (76.2)	7,382 (76.4)	36,803 (76.2)
African American (%)	4,491 (11.6)	1,071 (11.1)	5,562 (11.5)
Hispanic (%)	1,776 (4.6)	478 (5.0)	2,254 (4.7)
Asian (%)	579 (1.5)	146 (1.5)	725 (1.5)
Native American (%)	210 (0.5)	52 (0.5)	262 (0.5)
Other/Unknown (%)	2,153 (5.6)	529 (5.5)	2,682 (5.6)

MIMIC-IV data and preprocessing

We construct the cohort using ICU admissions from the MIMIC-IV 2.0 database and extract structured information from the diagnoses and chart events tables as patient features. The diagnoses are encoded as ICD-10 codes assigned by trained coders at hospital discharge. The chart events capture time-series physiological measurements and clinical observations. For each variable, we remove outliers by discarding values above a chosen upper percentile and below a chosen lower percentile across all records. We then aggregate the time series into 4-hour windows over the first 24 hours of ICU stay (yielding 6 time steps) and perform imputation using forward filling and mean imputation, resulting in a final representation in which chart events and derived statistical features form a 1925-dimensional time series for each record.

Supplementary Note 2 - Deep Neural Networts

Network Architectures

We employ a multi-layer perceptron (MLP) network for COVID-19 screening on CURIAL and CURIAL-Combined, and a long short-term memory (LSTM) network for both eICU tasks for in-hospital mortality prediction on MIMIC-IV dataset. We additionally incorporate a Transformer for mortality and shock prediction on the eICU dataset.

The network architectures are detailed in Supplementary Table 5. For the LSTM networks on eICU, the temporal dimension $T = 48$ time steps for in-hospital mortality prediction (corresponding to the 48-hour analysis window) and $T = 4$ time steps for shock prediction (reflecting 4-hour interval predictions). For the Transformer network on eICU dataset, it contains 2 self-attention layers, each with 8 attention heads (head dimension 64) and a 256-dimensional feed-forward network, using GELU activations and a sigmoid output layer for binary prediction. For the LSTM model on MIMIC-IV, the temporal dimension is set to $T = 6$ time steps for in-hospital mortality prediction, with each time step represented by a 1925-dimensional feature vector.

Supplementary Table 5: The network architectures for COVID-19 screening on CURIAL and CURIAL-Combined, the LSTM and Transformer network architectures for both eICU tasks, the LSTM network for MIMIC-IV dataset

.

**MLP**
Input variables (27 variables) $\rightarrow$ Dense Layer with 256 nodes $\rightarrow$ ReLU Activation $\rightarrow$ Dropout with 0.2 rate $\rightarrow$ Dense Layer with 128 nodes $\rightarrow$ ReLU Activation $\rightarrow$ Dropout with 0.2 rate $\rightarrow$ Dense Layer with 1 node $\rightarrow$ Sigmoid Activation
**LSTM** (eICU)
Input variables ($T \times 442$) $\rightarrow$ LSTM cell with 256 nodes $\rightarrow$ ReLU Activation $\rightarrow$ Dropout with 0.25 rate $\rightarrow$ Dense Layer with 1 node $\rightarrow$ Sigmoid Activation
**Transformer** (eICU)
Input variables ($T \times 442$) $\rightarrow$ 2-layer Transformer encoder with 8 attention heads per layer (head dimension 64, FFN dimension 256, GELU activation) $\rightarrow$ pooling $\rightarrow$ Dense layer with 1 node $\rightarrow$ Sigmoid activation
**LSTM** (MIMIC-IV)
Input variables ($T \times 1925$) $\rightarrow$ LSTM cell with 256 nodes $\rightarrow$ ReLU Activation $\rightarrow$ Dropout with 0.25 rate $\rightarrow$ Dense Layer with 1 node $\rightarrow$ Sigmoid Activation

Implementation Details

Supplementary Table 6: Hyper-parameter values for our fair unlearning framework (FU), and baseline unlearning methods across CURIAL, CURIAL-Combined, eICU and MIMIC-IV datasets.

Method	CURIAL	CURIAL-Combined	eICU (Mortality)	eICU (Shock)	MIMIC-IV
**FU**					
α (forgetting weight)	0.5	0.5	0.1	0.1	0.1
β (utility weight)	0.1	0.2	0.3	0.2	0.1
γ (fairness weight)	0.8	1	0.6	0.8	0.3
Learning rate η	1e-4	5e-3	8e-4	5e-4	5e-4
Epochs T	10	5	5	5	1
**GA**					
Learning rate	8e-4	5e-4	1e-4	5e-4	1e-4
Epochs	5	5	5	5	5
**CR**					
Learning Rate	0.1	0.7	0.5	0.1	0.3
Hessian computation factor	1e-4	1e-4	1e-4	1e-4	1e-4
**CF**					
Training layers	Last layer	Last layer	Last layer	Last layer	Last layer
Learning rate	8e-3	8e-4	8e-4	8e-4	1e-3
Epochs	5	5	5	5	2
**SCRUB**					
Distillation temperature	2.0	2.0	2.0	2.0	2.0
Unlearning weight	1	0.015	1	0.1	0.01
Distillation weight	1	0.01	1	0.1	0.01
Learning rate	1e-3	1e-3	1e-3	1e-3	2e-3
Epochs	10	10	10	10	5
**ORTHO**					
Forget Weight	0.1	0.1	0.1	0.1	0.1
Learning Rate	0.5	0.8	0.01	0.5	0.01
Epochs	10	15	10	10	10

Supplementary Table 6 provides the hyper-parameter values for our fair unlearning framework (FU), and baseline unlearning methods across CURIAL, CURIAL-Combined, eICU and MIMIC-IV datasets.

Supplementary Note 3 - Additional Results

Effects of forgetting set size on diagnostic performance and algorithmic fairness

We conduct a comprehensive evaluation of the effects of varying forgetting set sizes on both diagnostic performance and algorithmic fairness across CURIAL-Combined (Supplementary Figure 1), in-hospital mortality prediction task (Supplementary Figure 2), and shock prediction task from eICU (Supplementary Figure 3). The analysis examines forgetting ratios ranging from 1% to 10% in a 1% increment. We employ bubble plots as our visualisation technique, where bubble size indicates diagnostic performance (AUROC) and colour intensity represents the degree of algorithmic unfairness (measured by EO-TP and EO-FP metrics). Our FU method consistently exhibits superior performance across all evaluated datasets and tasks, characterised by larger bubble sizes (denoting higher diagnostic AUROC) and deeper colour saturation (signifying improved fairness), as forgetting ratios increase. Conversely, baseline methods exhibit markedly smaller bubbles (signifying diminished diagnostic performance) accompanied by lighter colouration (indicating greater algorithmic unfairness). These results reveal that our FU method delivers generalised improvements in both fairness and utility compared to baseline approaches, maintaining consistent superiority across diverse forgetting set sizes, datasets, and clinical prediction tasks.

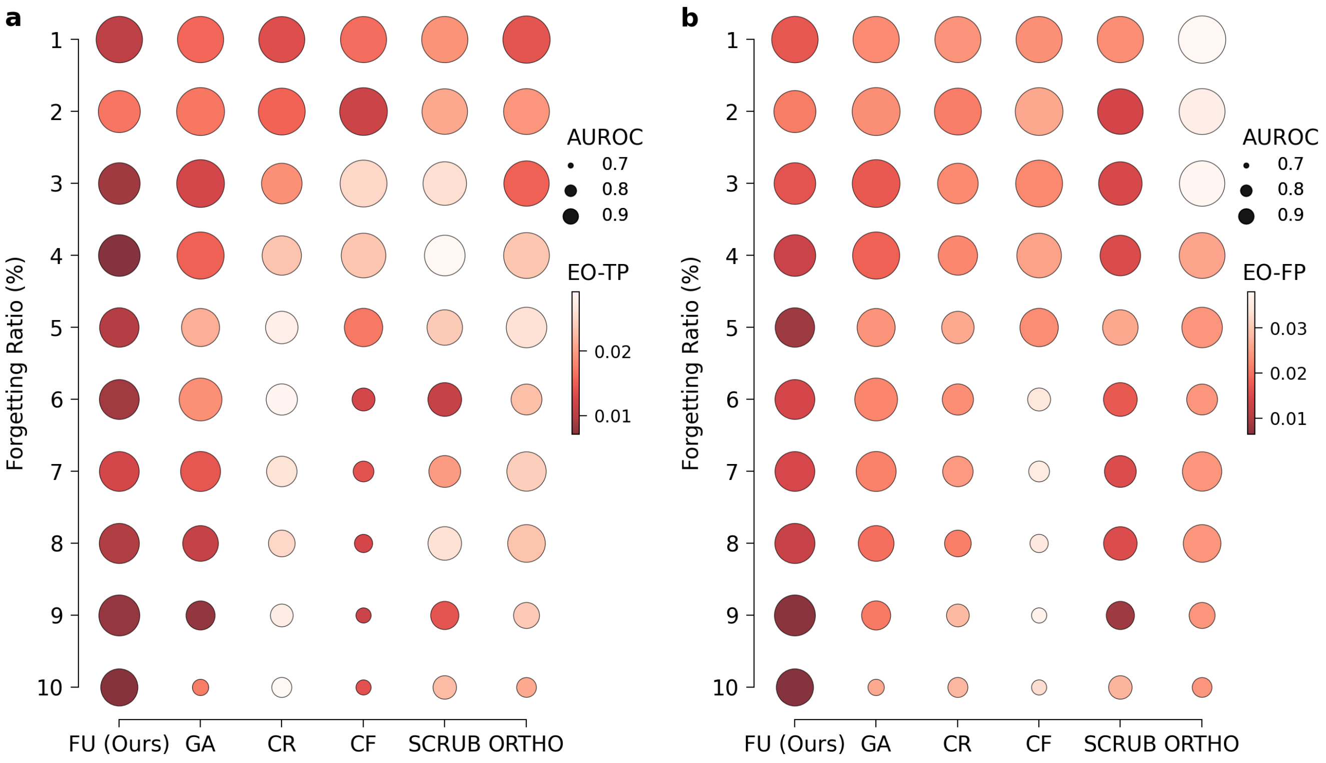

Supplementary Figure 1: Bubble plots to illustrate the effects of varying forgetting set sizes, from 1% forgetting ratio to 10%, on the algorithmic fairness (EO-TP and EO-FP) and diagnostic performance (AUROC) of different unlearning methods on CURIAL-Combined.

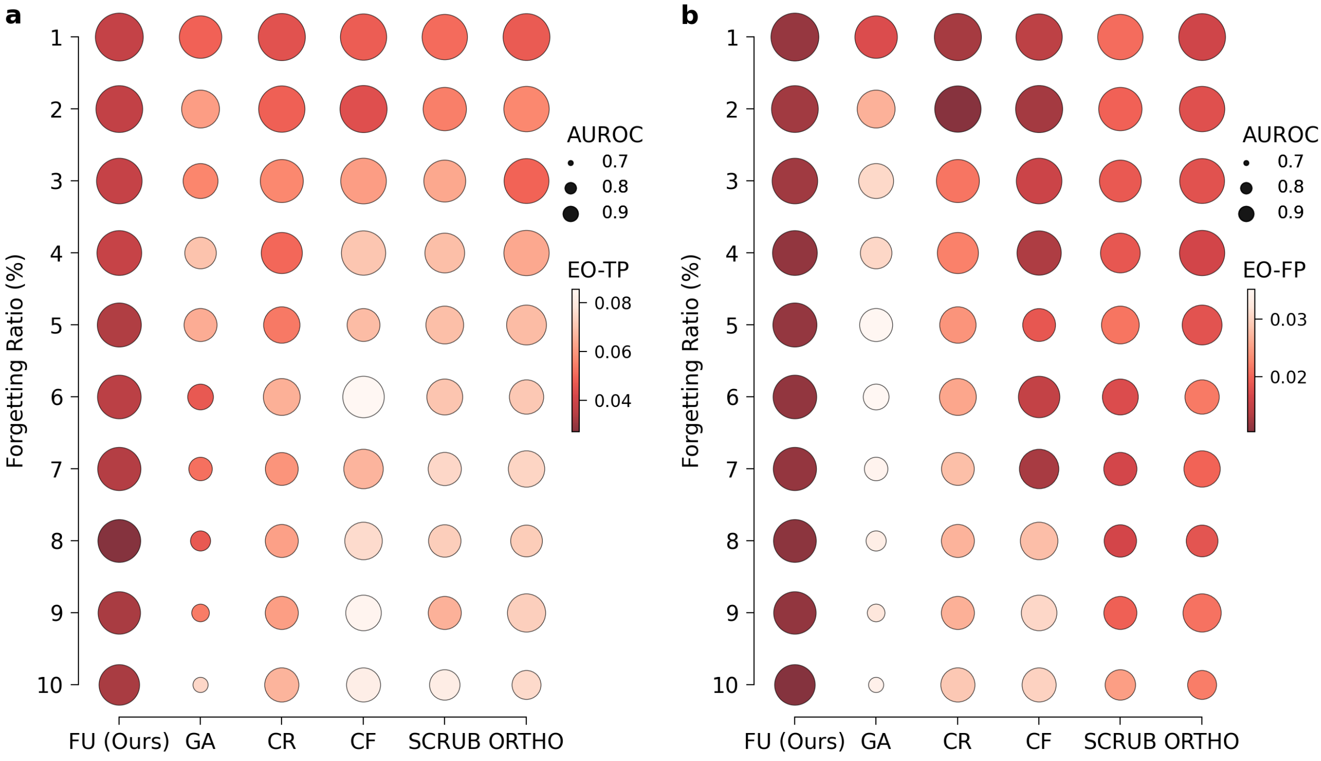

Supplementary Figure 2: Bubble plots to illustrate the effects of varying forgetting set sizes, from 1% forgetting ratio to 10%, on the algorithmic fairness (EO-TP and EO-FP) and diagnostic performance (AUROC) of different unlearning methods on mortality prediction of eICU.

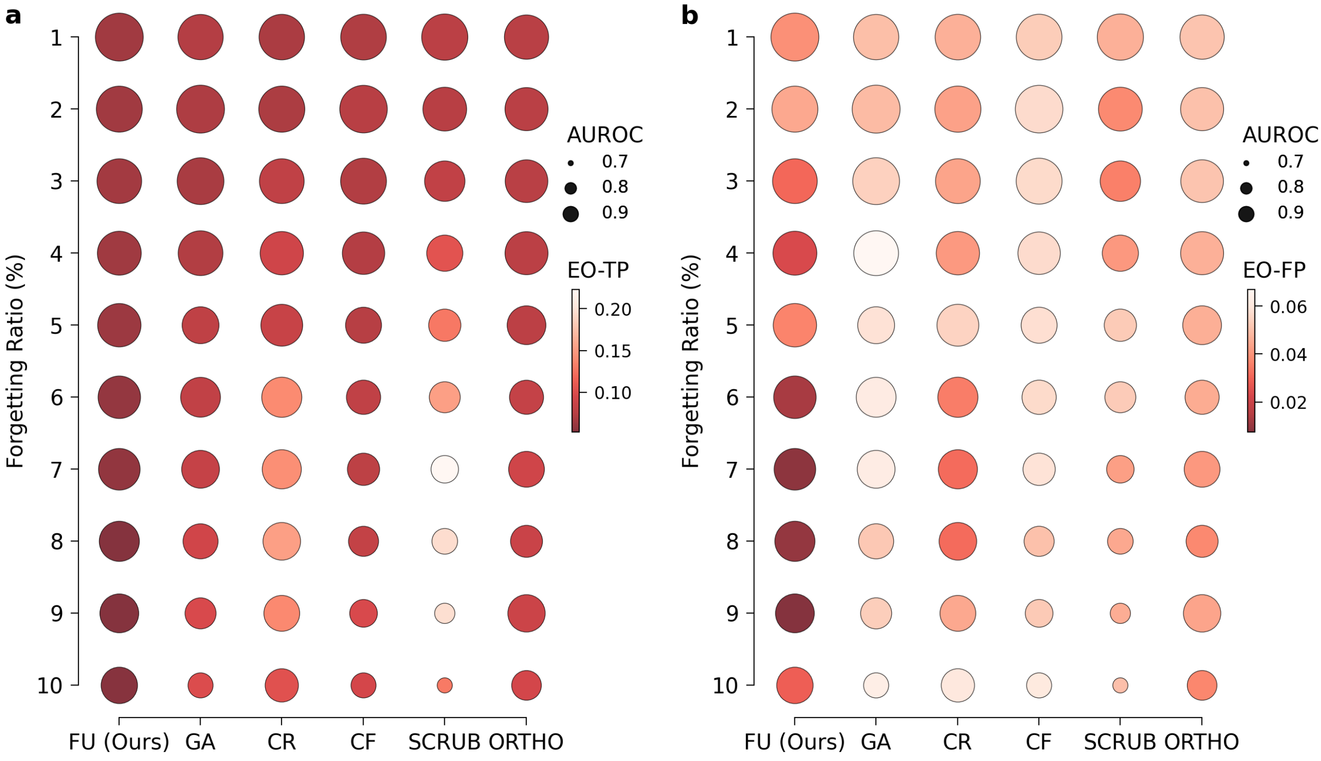

Supplementary Figure 3: Bubble plots to illustrate the effects of varying forgetting set sizes, from 1% forgetting ratio to 10%, on the algorithmic fairness (EO-TP and EO-FP) and diagnostic performance (AUROC) of different unlearning methods on shock prediction of eICU.

Unlearning effectiveness across different forgetting ratios

We employ the MIA-AUROC metric to assess unlearning effectiveness, with values approaching 0.5 indicating better privacy protection. Supplementary Figure 4 illustrates the MIA-AUROC performance across CURIAL, CURIAL-Combined, and eICU datasets under forgetting ratios of 1%, 5%, and 10%. A consistent trend emerges whereby increasing forgetting ratios yield MIA-AUROC values progressively closer to 0.5 across all methods, demonstrating more comprehensive forgetfulness. Notably, our FU method consistently outperforms baseline approaches at each forgetting ratio, achieving MIA-AUROC values closest to the optimal 0.5 threshold across all three datasets, thereby confirming its generalised effectiveness across diverse clinical data and prediction tasks.

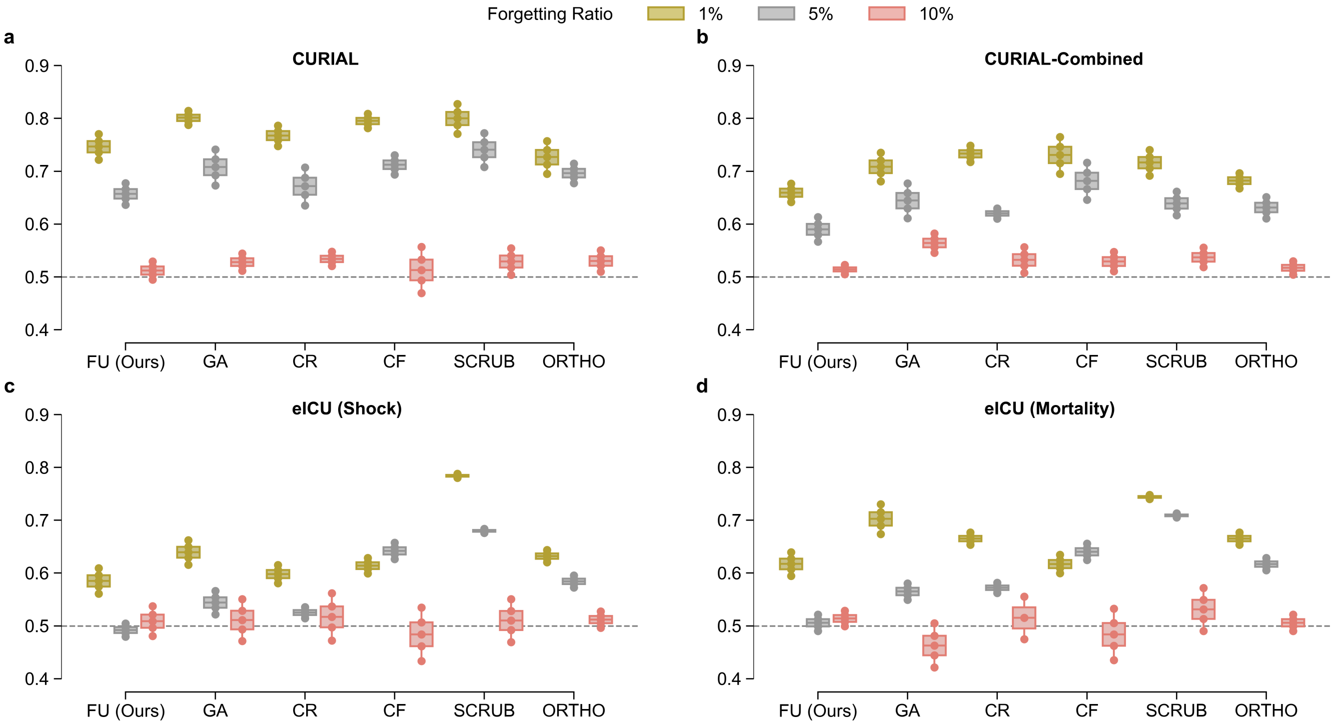

Supplementary Figure 4: Comparisons of unlearning effectiveness of various machine unlearning methods on CURIAL, CURIAL-Combined, and eICU datasets, across 1%, 5%, and 10% forgetting ratios. Each box plot displays the median (central line), IQR (box), and 1.5× IQR range (whiskers), from 5 independent runs.

Effects of fairness gradient weight in FU

Supplementary Table 7: Effects of fairness gradient weights (0.6 vs 1.0) on algorithmic fairness and diagnostic performance across CURIAL NHS trusts for COVID-19 screening.

	EO-TP ↓ (SD)				EO-FP ↓ (SD)				AUROC ↑ (SD)			
Forgetting Ratio	0%	1%	5%	10%	0%	1%	5%	10%	0%	1%	5%	10%
OUH (wave 2)												
Fairness Weight												
0.6	0.060	0.059	0.046	0.041	0.052	0.048	0.043	0.043	0.878	0.877	0.868	0.859
1	0.060	0.058	0.042	0.036	0.052	0.046	0.036	0.035	0.878	0.872	0.861	0.845
PUH (wave 2)												
Fairness Weight												
0.6	0.087	0.075	0.073	0.056	0.046	0.046	0.045	0.037	0.854	0.852	0.841	0.820
1	0.087	0.073	0.063	0.039	0.046	0.046	0.035	0.036	0.854	0.842	0.834	0.804
UHB (wave 2)												
Fairness Weight												
0.6	0.092	0.090	0.087	0.087	0.021	0.020	0.021	0.020	0.883	0.881	0.868	0.857
1	0.092	0.090	0.087	0.084	0.021	0.020	0.019	0.019	0.883	0.878	0.856	0.839
BH (wave 2)												
Fairness Weight												
0.6	0.148	0.140	0.106	0.103	0.151	0.149	0.140	0.134	0.890	0.884	0.877	0.868
1	0.148	0.137	0.096	0.90	0.151	0.145	0.138	0.134	0.890	0.868	0.857	0.848

We investigate the effects of fairness gradient weights on the balance between diagnostic performance and algorithmic fairness within our FU framework. Utilising the CURIAL dataset, we conduct sensitivity analyses by incrementally increasing the fairness gradient weight from 0.6 to 1.0, with results detailed in Supplementary Table 7.

We can observe that, whilst higher fairness gradient weights yield substantial improvements in algorithmic fairness, evidenced by reduced EO-TP and EO-FP values across all NHS trusts, the diagnostic performance presents a notably deterioration, as reflected by diminished AUROC scores. This phenomenon suggests that fairness enhancements inadvertently perturb the model's underlying knowledge architecture, resulting in collateral information degradation that extends beyond the intended unlearning objectives. Consequently, the fairness optimisation process introduces additional knowledge disruption, thereby intensifying the forgetting mechanism. These observations underscore the critical importance of judiciously calibrating the interdependencies amongst the three gradient components, i.e., unlearning, fairness, and utility, to achieve optimal knowledge structure modifications that concurrently enhance algorithmic fairness whilst preserving clinical efficacy.

Evaluations of computational and time complexity

Supplementary Table 8: Computational costs, measured by FLOPs and wall-clock time in seconds (s), between retraining and our FU on the LSTM network in eICU and the MLP network in CURIAL-Combined across 1%, 5%, and 10% forgetting ratios.

Forgetting Ratio	1%		5%		10%	
Methods	Retrained	FU	Retrained	FU	Retrained	FU
eICU (Shock) & LSTM						
FLOPs ($\times 10^8$)						
Total	1,193,500	98,500	1,143,800	94,400	188,300	16,000
Orthogonalisation (% of Total)	-	6.20 (0.01%)	-	5.94 (0.01%)	-	5.55 (0.03%)
Wall-clock Time (s)	47.25	4.44	45.94	4.10	43.65	3.87
CURIAL-Combined & MLP						
FLOPs ($\times 10^8$)						
Total	2,970	503	2,851	483	2,705	460
Orthogonalisation (% of Total)	-	0.65 (0.13%)	-	0.63 (0.13%)	-	0.60 (0.13%)
Wall-clock Time (s)	132.93	7.27	128.74	6.72	123.59	6.35

We have outlined the computational costs, evaluated in terms of FLOPs and wall-clock time (s), for retraining and our FU across different models and datasets in Supplementary Table 8. We can see that our FU greatly reduced the computational costs in both FLOPs and wall-clock time compared to retraining. This observation aligns with our anticipations, as our FU requires only a small number of unlearning steps (typically $<$ 100) to remove medical records, whereas retraining requires rerunning the entire training process with potentially thousands of training steps.

We also compute the computational overhead of gradient orthogonalisations in our FU, as shown in Supplementary Table 8. It can be seen that gradient orthogonalisations account for only a small proportion of FU's computations (typically $<$ 0.1%), as they are computed using the Gram-Schmidt algorithm, which adds a low computational overhead.

As such, we suggest that the computational cost is not a crucial factor when determining whether to perform retraining or not. Instead, we should comprehensively consider other essential metrics, such as the diagnostic performance and fairness, to decide whether retraining is required.

Scalability to more complex DNNs

Supplementary Table 9: Performance comparisons of unlearning methods between an LSTM of 20K parameters and a Transformer with 2.27M parameters on the eICU dataset (mortality prediction) across the 1%, 5%, and 10% forgetting ratios.

	EO-TP ↓ (SD)				EO-FP ↓ (SD)				DP ↓ (SD)			
Forg. Ratio	0%	1%	5%	10%	0%	1%	5%	10%	0%	1%	5%	10%
LSTM												
Original	0.041	-	-	-	0.014	-	-	-	0.021	-	-	-
FU (Ours)	-	**0.032** (0.001)	**0.039** (0.002)	**0.036** (0.001)	-	**0.013** (0.001)	0.018 (0.001)	**0.013** (0.001)	-	**0.019** (0.001)	**0.020** (0.000)	**0.019** (0.001)
GA	-	0.043 (0.006)	0.048 (0.010)	0.057 (0.003)	-	0.018 (0.002)	0.019 (0.002)	0.026 (0.003)	-	0.020 (0.004)	0.037 (0.005)	0.030 (0.003)
CR	-	0.044 (0.008)	0.077 (0.023)	0.068 (0.024)	-	0.013 (0.003)	0.024 (0.005)	0.028 (0.010)	-	0.022 (0.006)	0.036 (0.021)	0.034 (0.009)
CF	-	0.048 (0.004)	0.070 (0.005)	0.078 (0.014)	-	0.014 (0.003)	0.020 (0.010)	0.019 (0.003)	-	0.024 (0.006)	0.024 (0.004)	0.031 (0.006)
SCRUB	-	0.047 (0.009)	0.075 (0.014)	0.075 (0.019)	-	0.019 (0.008)	0.023 (0.004)	0.020 (0.005)	-	0.023 (0.008)	0.027 (0.007)	0.027 (0.004)
ORTHO	-	0.047 (0.014)	0.068 (0.006)	0.075 (0.023)	-	0.017 (0.002)	**0.017** (0.006)	0.022 (0.003)	-	0.022 (0.002)	0.021 (0.004)	0.026 (0.004)
Transformer												
Original	0.051	-	-	-	0.018	-	-	-	0.014	-	-	-
FU (Ours)	-	**0.048** (0.002)	**0.042** (0.001)	**0.039** (0.004)	-	**0.016** (0.002)	**0.014** (0.002)	**0.013** (0.001)	-	**0.012** (0.001)	**0.013** (0.002)	**0.012** (0.001)
GA	-	0.055 (0.009)	0.055 (0.004)	0.089 (0.009)	-	0.024 (0.005)	0.044 (0.004)	0.047 (0.002)	-	0.023 (0.004)	0.040 (0.004)	0.048 (0.003)
CR	-	0.048 (0.008)	0.060 (0.030)	0.072 (0.019)	-	0.024 (0.004)	0.024 (0.003)	0.023 (0.006)	-	0.023 (0.005)	0.024 (0.002)	0.022 (0.005)
CF	-	0.059 (0.020)	0.066 (0.020)	0.085 (0.016)	-	0.024 (0.006)	0.045 (0.002)	0.043 (0.003)	-	0.024 (0.003)	0.040 (0.002)	0.045 (0.002)
SCRUB	-	0.050 (0.014)	0.071 (0.010)	0.103 (0.009)	-	0.019 (0.005)	0.019 (0.008)	0.016 (0.006)	-	0.019 (0.006)	0.025 (0.006)	0.024 (0.008)
ORTHO	-	0.053 (0.015)	0.049 (0.028)	0.051 (0.016)	-	0.027 (0.004)	0.026 (0.008)	0.031 (0.010)	-	0.028 (0.007)	0.027 (0.007)	0.029 (0.009)

	AUROC ↑ (SD)				MIA-AUROC →0.5 (SD)			MI-KnnDist ↑ (SD)		
For. Ratio	0%	1%	5%	10%	1%	5%	10%	1%	5%	10%
LSTM										
Original	0.853	-	-	-	-	-	-	-	-	-
FU (Ours)	-	0.844 (0.001)	**0.829** (0.002)	**0.811** (0.001)	0.617 (0.017)	**0.506** (0.012)	0.514 (0.011)	**17.97** (0.605)	**19.02** (0.453)	**18.68** (0.710)
GA	-	0.814 (0.004)	0.773 (0.022)	0.747 (0.007)	0.702 (0.022)	0.565 (0.012)	0.463 (0.032)	12.62 (0.930)	15.40 (0.410)	16.77 (0.898)
CR	-	0.833 (0.004)	0.805 (0.009)	0.791 (0.021)	0.665 (0.009)	0.572 (0.007)	0.515 (0.040)	12.41 (0.765)	18.22 (0.461)	18.22 (0.416)
CF	-	**0.848** (0.003)	0.826 (0.003)	0.796 (0.019)	**0.617** (0.013)	0.640 (0.012)	0.484 (0.038)	13.13 (1.042)	14.37 (0.856)	17.21 (0.536)
SCRUB	-	0.840 (0.005)	0.808 (0.008)	0.784 (0.003)	0.744 (0.003)	0.709 (0.003)	0.531 (0.032)	12.42 (0.795)	16.06 (1.033)	18.62 (0.510)
ORTHO	-	0.845 (0.003)	0.816 (0.004)	0.780 (0.008)	0.665 (0.009)	0.617 (0.009)	**0.506** (0.012)	15.35 (0.866)	16.02 (0.604)	16.91 (0.395)
Transformer										
Original	0.874	-	-	-	-	-	-	-	-	-
FU (Ours)	-	0.852 (0.001)	0.835 (0.002)	**0.813** (0.003)	**0.768** (0.013)	0.696 (0.003)	**0.574** (0.004)	18.15 (0.301)	**20.93** (0.705)	**19.63** (0.452)
GA	-	0.851 (0.005)	0.813 (0.006)	0.785 (0.010)	0.807 (0.013)	0.702 (0.009)	0.599 (0.006)	**18.19** (0.241)	15.99 (0.463)	16.68 (0.559)
CR	-	0.847 (0.012)	**0.838** (0.011)	0.795 (0.025)	0.819 (0.011)	0.715 (0.029)	0.582 (0.020)	12.62 (0.910)	18.18 (0.565)	17.71 (0.646)
CF	-	0.847 (0.005)	0.813 (0.001)	0.790 (0.006)	0.786 (0.022)	0.712 (0.012)	0.604 (0.011)	12.74 (0.896)	13.77 (0.623)	15.26 (0.657)
SCRUB	-	**0.854** (0.004)	0.827 (0.007)	0.797 (0.006)	0.794 (0.027)	**0.691** (0.012)	0.617 (0.014)	14.33 (0.354)	18.42 (0.558)	18.19 (0.721)
ORTHO	-	0.840 (0.005)	0.829 (0.012)	0.796 (0.024)	0.797 (0.028)	0.704 (0.014)	0.601 (0.019)	12.40 (0.931)	18.94 (0.615)	14.67 (0.875)

To demonstrate the applicability of our approach to larger, more complex DNN architectures, we conduct additional evaluations on a 2-layer Transformer model[1] with 2.27M learnable parameters (detailed architecture outlined in Supplementary Table 5), in addition to the LSTM network with 20K learnable parameters on the eICU dataset for the mortality and shock prediction task. Supplementary Tables 9 and 10 compare the performance of those two networks for the mortality and shock prediction tasks on the eICU dataset, respectively. As shown in the tables, FU consistently outperforms baselines across algorithmic fairness (EO-TP, EO-FP, and DP), model utility (AUROC), and unlearning

Supplementary Table 10: Performance comparisons of unlearning methods between an LSTM of 20K parameters and a Transformer of 2.27M parameters on the eICU dataset (shock prediction) across the 1%, 5%, and 10% forgetting ratios.

	EO-TP ↓ (SD)				EO-FP ↓ (SD)				DP ↓ (SD)			
Forg. Ratio	0%	1%	5%	10%	0%	1%	5%	10%	0%	1%	5%	10%
**LSTM**												
Original	0.070	-	-	-	0.045	-	-	-	0.052	-	-	-
FU (Ours)	-	**0.058**(0.001)	**0.054**(0.008)	**0.048**(0.001)	-	**0.040**(0.001)	**0.040**(0.003)	**0.035**(0.001)	-	**0.044**(0.001)	**0.038**(0.001)	**0.027**(0.001)
GA	-	0.083(0.008)	0.083(0.003)	0.097(0.020)	-	0.044(0.003)	0.050(0.005)	0.076(0.009)	-	0.047(0.010)	0.047(0.011)	0.077(0.014)
CR	-	0.084(0.004)	0.087(0.010)	0.088(0.009)	-	0.050(0.008)	0.062(0.007)	0.056(0.005)	-	0.079(0.011)	0.062(0.012)	0.059(0.009)
CF	-	0.064(0.016)	0.091(0.012)	0.093(0.007)	-	0.045(0.010)	0.056(0.019)	0.056(0.006)	-	0.055(0.015)	0.048(0.007)	0.044(0.004)
SCRUB	-	0.083(0.016)	0.078(0.013)	0.089(0.017)	-	0.049(0.010)	0.056(0.008)	0.061(0.017)	-	0.045(0.007)	0.049(0.006)	0.044(0.012)
ORTHO	-	0.075(0.011)	0.077(0.009)	0.094(0.037)	-	0.051(0.004)	0.046(0.005)	0.037(0.008)	-	0.053(0.004)	0.049(0.004)	0.041(0.006)
**Transformer**												
Original	0.064	-	-	-	0.038	-	-	-	0.042	-	-	-
FU (Ours)	-	0.060(0.000)	**0.052**(0.001)	**0.049**(0.001)	-	0.036(0.007)	**0.034**(0.001)	**0.031**(0.001)	-	**0.031**(0.000)	**0.030**(0.001)	**0.030**(0.005)
GA	-	0.064(0.005)	0.062(0.003)	0.096(0.013)	-	0.046(0.014)	0.051(0.007)	0.072(0.022)	-	0.048(0.012)	0.046(0.008)	0.066(0.018)
CR	-	0.092(0.026)	0.107(0.045)	0.081(0.013)	-	**0.035**(0.009)	**0.034**(0.004)	0.037(0.009)	-	0.032(0.007)	0.037(0.005)	0.037(0.012)
CF	-	**0.052**(0.022)	0.067(0.008)	0.068(0.005)	-	0.036(0.007)	0.048(0.006)	0.049(0.012)	-	0.039(0.008)	0.046(0.005)	0.046(0.010)
SCRUB	-	0.085(0.020)	0.095(0.024)	0.081(0.016)	-	0.050(0.008)	0.050(0.010)	0.054(0.008)	-	0.050(0.008)	0.057(0.009)	0.061(0.007)

	AUROC ↑ (SD)				MIA-AUROC →0.5 (SD)			MI-KnnDist ↑ (SD)		
Forgetting Ratio	0%	1%	5%	10%	1%	5%	10%	1%	5%	10%
**LSTM**										
Original	0.862	-	-	-	-	-	-	-	-	-
FU (Ours)	-	**0.853**(0.000)	**0.841**(0.000)	**0.801**(0.000)	**0.585**(0.019)	**0.492**(0.010)	**0.509**(0.022)	**5.61**(0.176)	**5.62**(0.125)	**5.66**(0.109)
GA	-	0.845(0.002)	0.824(0.029)	0.749(0.038)	0.639(0.018)	0.544(0.017)	0.511(0.031)	4.31(0.350)	5.23(0.188)	5.37(0.085)
CR	-	0.852(0.002)	0.824(0.025)	0.788(0.036)	0.598(0.013)	0.525(0.008)	0.517(0.035)	4.58(0.301)	5.28(0.249)	5.19(0.149)
CF	-	0.846(0.006)	0.822(0.002)	0.790(0.008)	0.614(0.011)	0.642(0.012)	0.484(0.039)	5.50(0.270)	5.29(0.300)	5.38(0.175)
SCRUB	-	0.842(0.002)	0.806(0.004)	0.773(0.010)	0.784(0.003)	0.680(0.003)	0.510(0.032)	4.81(0.102)	5.17(0.213)	5.23(0.262)
ORTHO	-	0.845(0.002)	0.826(0.003)	0.784(0.018)	0.632(0.009)	0.584(0.009)	0.512(0.012)	4.69(0.312)	5.04(0.153)	5.23(0.214)
**Transformer**										
Original	0.874	-	-	-	-	-	-	-	-	-
FU (Ours)	-	**0.859**(0.000)	**0.844**(0.001)	**0.831**(0.001)	**0.687**(0.015)	**0.608**(0.025)	0.511(0.009)	**5.76**(0.223)	**5.69**(0.300)	**5.76**(0.127)
GA	-	0.853(0.012)	0.834(0.005)	0.735(0.006)	0.706(0.032)	0.627(0.020)	0.545(0.016)	5.41(0.131)	4.48(0.190)	4.68(0.180)
CR	-	0.853(0.009)	0.828(0.009)	0.800(0.034)	0.716(0.013)	0.664(0.014)	0.570(0.012)	4.51(0.075)	5.05(0.105)	5.46(0.088)
CF	-	0.848(0.007)	0.811(0.001)	0.803(0.015)	0.737(0.026)	0.646(0.020)	**0.498**(0.017)	4.84(0.063)	5.50(0.120)	5.36(0.150)
SCRUB	-	0.849(0.011)	0.826(0.008)	0.806(0.006)	0.747(0.019)	0.674(0.022)	0.538(0.010)	5.00(0.155)	5.04(0.164)	5.59(0.265)
ORTHO	-	0.845(0.009)	0.823(0.008)	0.801(0.010)	0.725(0.014)	0.664(0.036)	0.524(0.012)	3.99(0.157)	5.49(0.131)	5.12(0.174)

effectiveness (MIA-AUROC and MI-KnnDist), for both the LSTM (20K parameters) and Transformer (2.27M) architectures across two clinical tasks. Those results demonstrate that, although we mainly adopt DNN architectures typical of today's clinical AI, such as LSTMs and MLPs, our FU method could also generalise to a more complex and larger DNN architecture, Transformer, which is the core architecture of modern large language models (LLMs) or foundation models.

Additionally, we could observe some interesting findings by comparing the two DNNs' performance. As shown in Supplementary Tables 9 and 10, unlearning methods generally achieve higher MIA-AUROC with less desirable unlearning effectiveness for Transformer models than LSTM ones. The model's utility, as measured by AUROCs, is generally better for Transformers than for LSTMs.

Those observations indicate that larger and more complex DNNs tend to fit the training data better, i.e., memorise medical records more deeply and thoroughly, which could lead to higher diagnostic performance after unlearning but also makes them more vulnerable to privacy attacks. As such, how to more effectively remove patient information from complex architectures, such as Large Language Models (LLMs), can be an interesting area for future work.

Effects of additional privacy-preservation technique

Supplementary Table 11: Effects of differential privacy ("DiffPri") on unlearning performance for eICU shock prediction. A transformer network is first trained with and without differential privacy, and FU is applied to the two trained models with 1%, 5%, and 10% unlearning ratios. "FU + DiffPri" indicates that FU is applied to a transformer network trained with differential privacy.

	EO-TP ↓ (SD)			EO-FP ↓ (SD)			DP ↓ (SD)		
Forgetting Ratio	1%	5%	10%	1%	5%	10%	1%	5%	10%
Methods									
FU (Ours)	**0.060**(0.000)	**0.052**(0.001)	**0.049**(0.001)	**0.036**(0.007)	0.034(0.001)	**0.031**(0.001)	**0.031**(0.000)	**0.030**(0.001)	**0.030**(0.005)
FU + DiffPri	0.071(0.004)	0.069(0.000)	0.070(0.000)	0.038(0.003)	**0.032**(0.000)	0.031(0.002)	0.034(0.003)	0.030(0.000)	0.030(0.001)

	AUROC ↑ (SD)			MIA-AUROC →0.5 (SD)			MI-KnnDist ↑ (SD)		
Forgetting Ratio	1%	5%	10%	1%	5%	10%	1%	5%	10%
Methods									
FU (Ours)	**0.859**(0.000)	**0.844**(0.001)	**0.831**(0.001)	0.775(0.019)	0.687(0.010)	0.535(0.022)	5.76(0.223)	5.69(0.300)	5.76(0.127)
FU + DiffPri	0.838(0.000)	0.832(0.000)	0.829(0.000)	**0.702**(0.019)	**0.646**(0.010)	**0.504**(0.022)	**6.48**(0.124)	**6.61**(0.050)	**6.72**(0.143)

Data anonymisation[2], as the most commonly used method for protecting patient privacy in clinical practice, is the only privacy preservation technique applied to the datasets. To investigate the effect of additional privacy-preserving regimes, we incorporate differential privacy[3], one of the most prevalent techniques for privacy preservation in deep neural networks (DNNs), and evaluate its effects on our FU method. Specifically, we train a Transformer[1] (detailed architecture outlined in Supplementary Table 5) on the eICU dataset for the shock prediction task under two settings: with and without the differential privacy strategy. To implement differential privacy during training, we adopt stochastic gradient descent with gradient clipping, with a target privacy budget $(\varepsilon, \delta) = (4.0, 10^{-5})$ and a maximum gradient norm of 1.0. After that, we apply our FU to the two trained models to compare their performance after data removal.

The results are summarised in Supplementary Table 11, and we can observe that, in general, adding differential privacy provides stronger privacy protection for forgotten data, achieving better MIA-AUROCs (from membership inference attacks) and MI-KnnDists (from model inversion attacks) than without it. This phenomenon aligns with our expectations, as differential privacy, an effective privacy preservation technique, could provide an additional layer of privacy protection for patients to be forgotten when combined with our FU. This observation also demonstrates the compatibility of our FU with other privacy protection techniques. However, we can also observe trade-offs when employing differential privacy: the predictive (AUROC) and fairness (EO-TP, EO-FP, and DP) performance extensively degrade on unlearned models with differential privacy. The compromised performance can be reasonably attributed to the perturbations and noise introduced by differential privacy, which impair the model's discriminative power and disproportionately affect minority groups.

We can conclude that although adding another privacy-preserving technique, such as differential privacy, could improve the unlearning effectiveness, it will also undermine the model's utility and fairness. Additional computational overhead is also needed for those techniques. Therefore, there is a trade-off to consider. In general, however, the disadvantages of applying additional privacy-preserving techniques seem to outweigh their benefits, as our FU already provides sufficient privacy protection in most cases, and the reduced utility and fairness will be a commonly unacceptable cost.

Mitigation of intersectional unfairness

Supplementary Table 12: Diagnostic performance (AUROC) and unlearning effectiveness (MIA-AUROC and MI-KnnDist) of various methods on the CURIAL for ethnicity-age intersectional unfairness evaluations. Note that MIA-AUROC and MI-KnnDist are evaluated by privacy attacks on the forgetting sets from the training data.

Prospective evaluation on OUH

	AUROC ↑ (SD) (OUH wave 2)				MIA-AUROC →0.5 (SD)			MI-KnnDist ↑ (SD)		
Forg. Ratio	0%	1%	5%	10%	1%	5%	10%	1%	5%	10%
Original	0.878	-	-	-	-	-	-	-	-	-
Retrained	-	0.875(0.001)	0.875(0.001)	0.874(0.002)	0.510(0.006)	0.473(0.005)	0.501(0.007)	4.05(0.313)	4.76(1.278)	4.22(0.428)
Unlearning Methods										
FU (Ours)	-	**0.876**(0.000)	**0.857**(0.001)	**0.851**(0.001)	0.746(0.019)	**0.629**(0.010)	**0.492**(0.022)	**3.36**(0.437)	**3.90**(0.617)	**3.93**(0.408)
GA	-	0.870(0.004)	0.833(0.015)	0.787(0.027)	0.799(0.018)	0.706(0.017)	0.524(0.031)	3.19(0.331)	3.45(0.281)	3.68(0.200)
CR	-	0.872(0.002)	0.822(0.054)	0.814(0.012)	0.767(0.013)	0.682(0.008)	0.457(0.035)	3.10(0.258)	3.51(0.331)	3.57(0.190)
CF	-	0.872(0.004)	0.794(0.014)	0.781(0.003)	0.744(0.011)	0.701(0.012)	0.489(0.039)	3.35(0.275)	3.24(0.332)	3.28(0.132)
SCRUB	-	0.874(0.002)	0.851(0.002)	0.831(0.005)	0.778(0.003)	0.748(0.003)	0.461(0.032)	3.16(0.279)	3.31(0.289)	3.86(0.429)
ORTHO	-	0.867(0.007)	0.849(0.006)	0.822(0.013)	**0.731**(0.009)	0.657(0.009)	0.539(0.012)	3.31(0.369)	3.40(0.123)	3.53(0.160)

External evaluations

	AUROC ↑ (SD) (PUH wave 2)				AUROC ↑ (SD) (UHB wave 1)				AUROC ↑ (SD) (BH wave 2)			
Forg. Ratio	0%	1%	5%	10%	0%	1%	5%	10%	0%	1%	5%	10%
Original	0.858	-	-	-	0.886	-	-	-	0.891	-	-	-
Retrained	-	0.859 (0.003)	0.856 (0.003)	0.851 (0.004)	-	0.884 (0.002)	0.884 (0.002)	0.883 (0.002)	-	0.893 (0.002)	0.890 (0.003)	0.890 (0.003)
Unlearning Methods												
FU (Ours)	-	0.853 (0.000)	**0.832** (0.001)	**0.828** (0.001)	-	**0.883** (0.000)	**0.866** (0.002)	**0.868** (0.001)	-	**0.890** (0.000)	**0.870** (0.001)	0.861 (0.001)
GA	-	0.852 (0.009)	0.816 (0.020)	0.772 (0.028)	-	0.876 (0.010)	0.852 (0.025)	0.804 (0.042)	-	0.885 (0.005)	0.856 (0.018)	0.816 (0.013)
CR	-	0.851 (0.004)	0.789 (0.076)	0.779 (0.015)	-	0.882 (0.003)	0.824 (0.064)	0.818 (0.024)	-	0.886 (0.003)	0.839 (0.046)	0.829 (0.020)
CF	-	0.854 (0.003)	0.774 (0.013)	0.768 (0.000)	-	0.881 (0.007)	0.794 (0.020)	0.803 (0.017)	-	0.880 (0.010)	0.835 (0.023)	0.827 (0.001)
SCRUB	-	**0.857** (0.002)	0.832 (0.005)	0.827 (0.010)	-	0.881 (0.002)	0.854 (0.009)	0.848 (0.011)	-	0.887 (0.002)	0.867 (0.011)	0.842 (0.013)
ORTHO	-	0.847 (0.016)	0.823 (0.023)	0.791 (0.042)	-	0.877 (0.009)	0.860 (0.012)	0.846 (0.029)	-	0.886 (0.004)	0.868 (0.010)	**0.864** (0.010)

Real-world disparities may also arise from intersectional unfairness, i.e., performance inequalities across subpopulations stratified by joint attributes. As such, we evaluate the performance of various unlearning methods against intersectional unfairness on the CURIAL dataset by jointly stratifying patients by ethnicity and age groups (see Table 1 of the manuscript for cohort statistical information). This stratification yields 21 ethnicity-age subgroups (e.g., whites aged 40-64, Chinese aged 65+), across which the intersectional unfairness is evaluated. Similar to our prior experiments on the CURIAL dataset, we first train an MLP on OUH wave 1 data and OUH pre-pandemic controls, and we evaluate the unlearned model on the wave 2 data of OUH, PUH, PH, and wave 1 of UHB.

Supplementary Table 13: Ethnicity-age intersectional algorithmic unfairness of unlearned MLP models of various machine unlearning methods on the CURIAL dataset.

	EO-TP ↓ (SD)				EO-FP ↓ (SD)				DP ↓ (SD)			
Forg. Ratio	0%	1%	5%	10%	0%	1%	5%	10%	0%	1%	5%	10%
OUH (wave 2)												
Original	0.131	-	-	-	0.062	-	-	-	0.121	-	-	-
Retrained	-	0.144(0.002)	0.146(0.005)	0.142(0.006)	-	0.060(0.006)	0.060(0.004)	0.065(0.004)	-	0.119(0.006)	0.120(0.003)	0.122(0.003)
Unlearning Methods												
FU (Ours)	-	**0.131**(0.001)	**0.127**(0.001)	**0.113**(0.002)	-	**0.062**(0.000)	**0.060**(0.000)	**0.055**(0.003)	-	**0.119**(0.000)	0.116(0.001)	**0.107**(0.001)
GA	-	0.143(0.010)	0.157(0.005)	0.152(0.012)	-	0.068(0.009)	0.136(0.014)	0.152(0.052)	-	0.126(0.006)	0.171(0.053)	0.165(0.038)
CR	-	0.140(0.007)	0.145(0.028)	0.158(0.021)	-	0.067(0.005)	0.082(0.024)	0.063(0.007)	-	0.123(0.004)	0.123(0.013)	0.109(0.017)
CF	-	0.144(0.008)	0.159(0.052)	0.147(0.005)	-	0.074(0.017)	0.115(0.065)	0.080(0.035)	-	0.129(0.011)	0.175(0.059)	0.165(0.006)
SCRUB	-	0.131(0.003)	0.146(0.008)	0.162(0.013)	-	0.063(0.003)	0.063(0.010)	0.069(0.010)	-	0.122(0.003)	**0.115**(0.003)	0.109(0.006)
ORTHO	-	0.149(0.019)	0.153(0.024)	0.153(0.020)	-	0.074(0.010)	0.096(0.028)	0.077(0.015)	-	0.124(0.010)	0.135(0.026)	0.137(0.022)
PUH (wave 2)												
Original	0.235	-	-	-	0.171	-	-	-	0.185	-	-	-
Retrained	-	0.201(0.046)	0.203(0.045)	0.187(0.043)	-	0.145(0.012)	0.142(0.018)	0.138(0.006)	-	0.167(0.010)	0.166(0.012)	0.157(0.010)
Unlearning Methods												
FU (Ours)	-	**0.168**(0.037)	**0.179**(0.017)	**0.165**(0.011)	-	**0.168**(0.001)	**0.156**(0.001)	**0.142**(0.000)	-	0.180(0.002)	**0.176**(0.003)	0.170(0.002)
GA	-	0.214(0.047)	0.226(0.043)	0.230(0.031)	-	0.174(0.013)	0.156(0.019)	0.157(0.037)	-	0.181(0.008)	0.176(0.014)	0.176(0.030)
CR	-	0.220(0.042)	0.215(0.038)	0.246(0.022)	-	0.169(0.010)	0.161(0.013)	0.167(0.013)	-	**0.179**(0.005)	0.179(0.011)	0.170(0.011)
CF	-	0.240(0.021)	0.209(0.010)	0.213(0.005)	-	0.171(0.011)	0.156(0.006)	0.171(0.025)	-	0.182(0.004)	0.179(0.011)	0.173(0.029)
SCRUB	-	0.219(0.033)	0.208(0.035)	0.230(0.042)	-	0.170(0.011)	0.162(0.012)	0.143(0.008)	-	0.180(0.006)	0.178(0.007)	**0.166**(0.011)
ORTHO	-	0.186(0.046)	0.218(0.036)	0.227(0.050)	-	0.168(0.004)	0.165(0.014)	0.168(0.043)	-	0.182(0.018)	0.176(0.006)	0.182(0.030)
UHB (wave 1)												
Original	0.174	-	-	-	0.044	-	-	-	0.046	-	-	-
Retrained	-	0.178(0.032)	0.188(0.034)	0.186(0.033)	-	0.049(0.006)	0.048(0.005)	0.046(0.005)	-	0.050(0.006)	0.049(0.005)	0.048(0.005)
Unlearning Methods												
FU (Ours)	-	0.230(0.000)	**0.239**(0.001)	**0.229**(0.002)	-	**0.045**(0.000)	0.064(0.002)	**0.049**(0.002)	-	**0.045**(0.000)	0.065(0.001)	**0.050**(0.001)
GA	-	0.230(0.013)	0.241(0.003)	0.236(0.010)	-	0.054(0.007)	0.095(0.024)	0.091(0.017)	-	0.055(0.008)	0.096(0.024)	0.091(0.016)
CR	-	0.236(0.042)	0.241(0.031)	0.238(0.023)	-	0.051(0.006)	**0.060**(0.015)	0.049(0.002)	-	0.052(0.006)	**0.060**(0.014)	0.051(0.003)
CF	-	0.233(0.019)	0.240(0.022)	0.237(0.031)	-	0.049(0.011)	0.099(0.013)	0.120(0.001)	-	0.051(0.011)	0.100(0.014)	0.121(0.001)
SCRUB	-	**0.224**(0.022)	0.242(0.018)	0.231(0.014)	-	0.046(0.004)	0.065(0.003)	0.050(0.006)	-	0.046(0.002)	0.065(0.003)	0.053(0.005)
ORTHO	-	0.236(0.025)	0.249(0.022)	0.231(0.021)	-	0.050(0.016)	0.068(0.025)	0.072(0.029)	-	0.051(0.016)	0.068(0.025)	0.073(0.029)
BH (wave 2)												
Original	0.269	-	-	-	0.154	-	-	-	0.238	-	-	-
Retrained	-	0.272(0.005)	0.292(0.028)	0.271(0.006)	-	**0.153**(0.004)	0.142(0.029)	0.124(0.026)	-	0.236(0.002)	0.233(0.007)	0.226(0.010)
Unlearning Methods												
FU (Ours)	-	**0.269**(0.000)	**0.241**(0.049)	**0.231**(0.013)	-	**0.153**(0.000)	0.168(0.000)	**0.159**(0.007)	-	**0.237**(0.000)	**0.231**(0.006)	**0.227**(0.012)
GA	-	0.277(0.032)	0.260(0.006)	0.279(0.028)	-	0.155(0.015)	0.167(0.015)	0.178(0.017)	-	0.250(0.039)	0.231(0.003)	0.239(0.014)
CR	-	0.288(0.022)	0.269(0.038)	0.273(0.039)	-	0.155(0.004)	**0.157**(0.041)	0.166(0.016)	-	0.242(0.005)	0.234(0.020)	0.228(0.013)
CF	-	0.269(0.004)	0.276(0.030)	0.256(0.000)	-	0.159(0.008)	0.170(0.020)	0.165(0.010)	-	0.240(0.007)	0.237(0.016)	0.234(0.017)
SCRUB	-	0.271(0.003)	0.278(0.026)	0.274(0.068)	-	0.155(0.005)	0.166(0.008)	0.161(0.027)	-	0.239(0.007)	0.235(0.012)	0.228(0.013)
ORTHO	-	0.269(0.006)	0.275(0.031)	0.260(0.072)	-	0.156(0.004)	0.168(0.013)	0.159(0.013)	-	0.237(0.010)	0.235(0.007)	0.229(0.015)

Supplementary Table 13 reports the intersectional fairness results (EO-TP, EO-FP, and DP) across the four NHS trust cohorts. Compared to baseline methods, our FU consistently and effectively mitigate intersectional disparities across the ethnicity-age subgroups on the four NHS trust cohorts. More favourably, as shown in Supplementary Table 12, our FU still achieves superior predictive performance (higher AUROCs) than all unlearning baselines across the four cohorts, and it also more thoroughly removes patient information from the forgetting set, as evidenced by both higher MIA-AUROC and MI-KnnDist values. As such, we have shown that our FU can also generalise to the scenarios of intersectional unfairness, thereby increasing its potential for real-world applications.

Determination of clinical thresholds

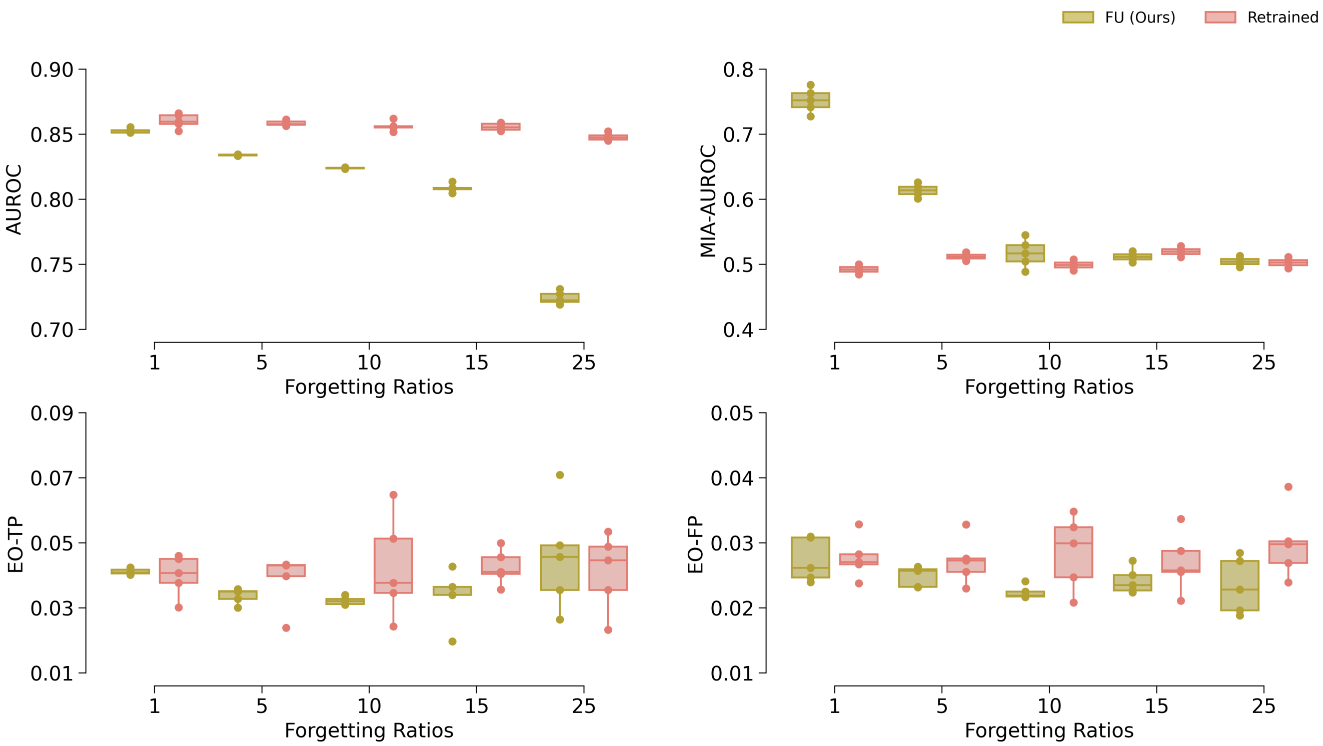

Supplementary Figure 5: Performance comparisons of unlearned models by our FU and the retrained models across forgetting ratios from 1% to 25%, under the incremental forgetting protocol of consecutive 1% increments, on the mortality prediction task of MIMIC-IV. Each box plot displays the median (central line), IQR (box), and 1.5× IQR range (whiskers), from 5 independent runs.

In clinical deployment, a practical guide on the threshold for acceptable performance drops can be necessary. To address this issue, we first thoroughly investigate the performance of a retrained model and an unlearned model by our FU across a broader range of forgetting ratios. Specifically, on the MIMIC-IV dataset, we remove patient records from 1% to 25% forgetting ratios with a 1% increment, and plot the performance of FU and retraining at five forgetting ratios: 1%, 5%, 10%, 15%, 20% and 25% in Supplementary Figure 5. As shown in the figure, FU exhibits a progressive decline in model utility, with AUROC dropping from 0.85 to 0.73, thereby widening the performance gap relative to the retrained baseline, which shows slightly lower AUROCs. FU's fairness metrics, such as EO-TP and EO-FP, generally exhibit superior performance over retraining across various forgetting ratios. Meanwhile, MIA-AUROC gradually approaches the retraining level (close to 0.5) as the forgetting ratio increases, illustrating more thorough forgetting and better privacy protections.

Those observations imply that model utility can be a more crucial metric for determining whether a full retraining is necessary for our FU algorithm, depending on the clinical scenarios encountered upon deployment. For example, a tiered monitoring system can be adopted on the predictive performance of the unlearning model with the following rules: a 3-5% AUROC drop will trigger a yellow warning flag indicating more intensive monitoring, a 5-10% AUROC drop will incur a red flag for re-investigating the necessity of retraining, and a drop exceeding 10% will launch a retraining to be conducted immediately. Additional flagging systems for fairness or privacy protection metrics can be developed with similar criteria for performance drop and combined to support more comprehensive, real-time monitoring. While those criteria are set following empirical rules, the complexity and challenges of

clinical deployments will require those thresholds to be adjusted accordingly.

Supplementary References

1. Vaswani, A., Shazeer, N., Parmar, N., Uszkoreit, J., Jones, L., Gomez, A.N., Kaiser, Ł., and Polosukhin, I. (2017). Attention is all you need. Advances in neural information processing systems *30*.

2. El Emam, K., and Arbuckle, L. (2013). Anonymizing Health Data: Case Studies and Methods to Get You Started. 1st ed.. Sebastopol: O'Reilly Media, Inc. ISBN 1449363075.

3. Abadi, M., Chu, A., Goodfellow, I., McMahan, H.B., Mironov, I., Talwar, K., and Zhang, L. (2016). Deep learning with differential privacy. In Proceedings of the 2016 ACM SIGSAC conference on computer and communications security. pp. 308–318.
