# Supplementary material for: Mitigating algorithmic unfairness arising from forgetfulness of medical records in clinical artificial intelligence
Source: Nat Commun. 2026 May 4;17:6009. doi: 10.1038/s41467-026-72601-7 (PMC13346932; doi:10.1038/s41467-026-72601-7)
Supplement: Supplementary file 3 — Transparent Peer Review file [file 41467_2026_72601_MOESM3_ESM.pdf]

nature portfolio

Peer Review File

Mitigating algorithmic unfairness arising from forgetfulness of medical records in clinical artificial intelligence

Corresponding Author: Dr Yujiang Wang

This file contains all reviewer reports in order by version, followed by all author rebuttals in order by version.

Version 0:

Reviewer comments:

Reviewer #1

(Remarks to the Author)
Summary:

Motivated by the patient's right to be forgotten (RtbF), the manuscript proposes a strategy for unlearning which effectively forgets the records, preserves predictive performance for the remaining records (i.e., retain set) and ensures fairness by mitigating potential impacts of unlearning on demographic unfairness across subpopulations in the health domain. The paper provides an extensive evaluation using real-world multi-hospital datasets of COVID-19 screening, in-hospital mortality and shock predictions. Unlearning negatively affects fairness and there is a close link between fairness and orthogonalization. The paper combines these and proposes an unlearning strategy based on gradient orthogonalization. Existing methods use orthogonalization to mitigate interference between gradients of forget and remaining sets. The manuscript computes gradients using three loss functions (utility, forgetting and fairness) by projecting the forgetting gradient onto a subspace orthogonal to the utility and fairness.

Comments:

The abstract and introduction could have provided more information on the unlearning strategy and novelty.

Understandably, the manuscript highlights patients' right to be forgotten (RtbF), enabling trust in AI in the health domain to better align with Nature Communications. However, the paper could be organised to explain the methodology before delving too deeply into the experiments on the datasets.

There is a bit of repetition and redundancy in the text. The motivation of the work and experimental details are repeated excessively, leading to inefficient use of space.

The manuscript uses real-world, multiple-site datasets with demographic features. While this is quite helpful to evaluate the fairness of the proposed approach, is it sufficient to validate the generic fairness claims of the paper? Additional datasets with distinct features to cover other fairness metrics would strengthen the paper. I would like to note that the fairness aspect is the one that highlights the benefit of the gradient orthogonalisation approach to unlearning.

The datasets were anonymised. Were there further privacy preservation techniques applied to the datasets? The behaviour of the proposed scheme under different privacy-preserving regimes could be of great interest.

I did not check all the tables providing a summary for datasets. However, the numbers presented in Table 1 do not fully add up, e.g., ethnicity totals do not match to the cohort totals or percentages add up to >100%.

Do any of the baseline algorithms attempt to optimise the fairness as well? Given that the latest baseline dates back to 2023 and the publication of unlearning schemes based on gradient orthogonalisation in 2024 and 2025, are there potentially better baselines? Related work should cover the most recent works due to the recent increasing popularity of the topic.

Authors may like to put supporting evaluations to highlight the computation and time complexity gap between retraining and unlearning for different models and dataset sizes. Considering the additional overhead introduced by the gradient

orthogonalisation, at what forgetting rate can retraining be considered as an option?

Overall evaluations focus on one round of forgetting, which is not realistic. Forgetting can be handled in batches of 1%, 5%, or 10%, but these will be repeated over specific time frames. How do the presented performance metrics for utility, forgetting and fairness change throughout the forgetting rounds?. Would it be possible to talk about a bound or budget at which retraining becomes essential?

How do the model choice and model size impact the results and practicality of the solution?

The contribution of the paper and its novelty should be better articulated. Specifically, missing related work published between 2024 and 2025 does not help. There are comparable works using gradient orthogonalization as a fair unlearning approach, e.g., mitigating interference between gradients of forget and remaining set (https://arxiv.org/abs/2503.02312).

(Remarks on code availability)

Reviewer #2

(Remarks to the Author)
The manuscript tackles a very timely and critical challenge in AI for health applications on how to implement RtbF for patient data in deployed machine learning models, while maintaining fairness, biases detection and utility. The authors focus specifically on the risks that standard machine unlearning methods can exacerbate algorithmic unfairness, introducing “fair unlearning” approach that enforces orthogonality between unlearning, utility, and fairness objectives. From the privacy perspective, the paper adresses consistentily this angle. The use of membership inference attacks to quantitatively assess whether patient data has truly been “forgotten” is reasonable and well executed. The results demonstrate that their FU framework achieves MIA-AUROC scores close to .5, suggesting effective removal of sensitive records and therefore strong patient data privacy. However, it’s worth noting that privacy evaluation is limited to this recognized standard, but not exhaustive. Other plausible attack vectors, such as model inversion or attribute inference, are not addressed. The authors could strengthen their privacy claims by at least acknowledging these limitations and discussing the complexities of real-world deployments (for example, tracking and managing unlearning requests in federated settings, or the cumulative risk of sequential unlearning).

On the transparency and explainability angle, the manuscript offers strong contribution. The use of equalized odds as a fairness metric is transparent and interpretable for non-technical stakeholders, and the data visualization set presented are informative. The “Algorithmic insights” section gives a rare, welcome look into why their approach works, which can foster trust among health professionals. However, the clinical explainability of model decisions, particularly post-unlearning, is not explored as it deserves. The paper doesn't show, for instance, whether feature importances or decision logic change after unlearning. Health professionals may want to know how the model’s “thinking” shifts, especially if the system is being actively modified in production. Making this point clearer would bring a more consistent explainability aspect for the proposed approach.

On another aspect, fairness is assessed only along single dimensions (ethnicity, hospital), whereas intersectional unfairness —often where real-world disparities emerge—remains unaddressed. Similarly, the performance-fairness trade-off is recognized, but the paper could suggest practical thresholds for clinical deployment, which could be very useful for practitioners.

For improvements, I’d suggest the following:
1. broaden the privacy discussion to include more attack types and practical deployment challenges;
2. consider how fairness metrics and unlearning effectiveness could be communicated in real time to clinicians (for example, via dashboards or alerts or even some sort of system's ethics-panel; 3. provide some analysis of post-unlearning model explainability for individual predictions. Furthermore, intersectional fairness and explicit clinical thresholds for “acceptable” drops in model performance would help health systems make better informed decisions about deploying and maintaining these models.

Finally, while the approach is tested on neural networks typical of today’s clinical AI, some discussion of scaling the method to larger/complex architectures (such as transformers or multimodal models) would be relevant as a helpful perspective. That could help readers to understand what's is coming or even wide derivations of this research topic.

(Remarks on code availability)
Overall, the code as-is is hard to run “out of the box”. Imports break because there’s no clear package hierarchy, magic numbers (e.g. hard-coded input dims) and a missing forward() in the LSTM mean nothing actually executes, hyperparameters and seeding live in three different scripts, and there’s no requirements file to tell you how to install dependencies or fetch the CURIAL/eICU data. You’d spend more time fixing broken imports, wiring up data loaders, and guessing which flags to pass than actually experimenting with unlearning methods. A simple reorganization into a proper models/, training/, and utils/ package, a unified config (or shared flags), correct docstrings, global seeding, plus a short README and pinned requirements.txt would make it usable and reproducible.

Version 1:

Reviewer comments:

Reviewer #1

(Remarks to the Author)
The revised version and the rebuttal address the concerns I previously reported.

I previously raised concerns on:
Clarity of paper, typos in results, and redundancies
Evaluation on additional datasets with distinct features
Behaviour of the proposed scheme under different privacy-preserving regimes
More recent baselines
Computation and complexity
Impact of multiple rounds of forgetting and the decision point for retraining
Impact of model and model size

I appreciate the answers and revisions in response to those comments. I have no further comments.

(Remarks on code availability)
I had a look at the repository. I did not attempt to run and cross-check the results. The instructions for running the code are clear and straightforward. The code has sufficient comments to follow.

Reviewer #2

(Remarks to the Author)
Authors have provided further clarifications and reflected these changes in the main text, proper addressing all my previous comments.
The work will contribute towards responsible AI frameworks, bringing a strong contribution to the state-of-art. Methods are solid and consistent as well as the way authors reported into the rebuttal letter in a comprehensive manner, detailing the rationals to support both reviewer comments and arguments.
Rest assured, I recommend the paper for publication.

(Remarks on code availability)

Open Access This Peer Review File is licensed under a Creative Commons Attribution 4.0 International License, which permits use, sharing, adaptation, distribution and reproduction in any medium or format, as long as you give appropriate credit to the original author(s) and the source, provide a link to the Creative Commons license, and indicate if changes were made.
In cases where reviewers are anonymous, credit should be given to 'Anonymous Referee' and the source.
The images or other third party material in this Peer Review File are included in the article’s Creative Commons license, unless indicated otherwise in a credit line to the material. If material is not included in the article’s Creative Commons

license and your intended use is not permitted by statutory regulation or exceeds the permitted use, you will need to obtain permission directly from the copyright holder.
To view a copy of this license, visit https://creativecommons.org/licenses/by/4.0/

Response to Reviewer #1

Comment 1. *Summary: Motivated by the patient's right to be forgotten (RtbF), the manuscript proposes a strategy for unlearning which effectively forgets the records, preserves predictive performance for the remaining records (i.e., retain set) and ensures fairness by mitigating potential impacts of unlearning on demographic unfairness across subpopulations in the health domain. The paper provides an extensive evaluation using real-world multi-hospital datasets of COVID-19 screening, in-hospital mortality and shock predictions. Unlearning negatively affects fairness and there is a close link between fairness and orthogonalization. The paper combines these and proposes an unlearning strategy based on gradient orthogonalization. Existing methods use orthogonalization to mitigate interference between gradients of forget and remaining sets. The manuscript computes gradients using three loss functions (utility, forgetting and fairness) by projecting the forgetting gradient onto a subspace orthogonal to the utility and fairness.*
Comments: The abstract and introduction could have provided more information on the unlearning strategy and novelty.
Understandably, the manuscript highlights patients' right to be forgotten (RtbF), enabling trust in AI in the health domain to better align with Nature Communications. However, the paper could be organised to explain the methodology before delving too deeply into the experiments on the datasets.
There is a bit of repetition and redundancy in the text. The motivation of the work and experimental details are repeated excessively, leading to inefficient use of space.

Reply 1. Thank you for the comment. We have revised Sections "Abstract" and "Introduction" in the manuscript to better explain the unlearning strategy and provide more methodology information. We have also simplified or reduced the repetition of motivation descriptions in the manuscript to utilise space more efficiently.

Comment 2. *The manuscript uses real-world, multiple-site datasets with demographic features. While this is quite helpful to evaluate the fairness of the proposed approach, is it sufficient to validate the generic fairness claims of the paper? Additional datasets with distinct features to cover other fairness metrics would strengthen the paper. I would like to note that the fairness aspect is the one that highlights the benefit of the gradient orthogonalisation approach to unlearning.*

Reply 2. Thank you for the comment. We have additionally adopted a large-scale, real-world medical dataset, a new fairness metric, a new privacy protection metric, and a new unlearning baseline to better validate the claims of the proposed FU method.

Specifically, we perform additional evaluations on Medical Information Mart for Intensive Care IV (MIMIC-IV)[1]. The MIMIC-IV dataset contains de-identified electronic health records (EHRs) from patients admitted to intensive care units (ICUs) at Beth Israel Deaconess Medical Centre in Boston, Massachusetts, between 2008 and 2019. We generally follow the existing protocol[2] to curate and pre-process 54,831 EHRs (see Supplementary Note 1 for details) collected during the first 24 hours after ICU admission into time-series data. The clinical task is to predict in-hospital mortality. We split all the 54,831 records into an 80:20 ratio for training and testing, and we train a long short-term memory (LSTM) network to evaluate the performance of various unlearning methods.

Moreover, we employ Demographic Parity (DP)[3] as an additional fairness metric. DP measures differences in the positive prediction rates across demographic groups, concentrating on selection-rate disparities independent of ground-truth labels and thus effectively complementing the adopted

Equalised Odds (EO-TPs and EO-FPs). We compute DP as the standard deviation of positive prediction rates across sensitive subpopulations; lower DP indicates less disparities in selection rates and thus better fairness.

To better verify the privacy protections of unlearning methods, i.e., unlearning effectiveness, we implement an additional privacy attack paradigm, model inversion (MI), and report its results as a new privacy protection metric. Specifically, MI aims to reconstruct the training data by querying or analysing the model outputs, and we follow the MI attack implementation in the prior work[4] to assess the privacy protections of patients' data to be forgotten. The unlearning effectiveness under the MI attack is evaluated by the K-Nearest Neighbour Distance[5], denoted as MI-KnnDist. MI-KnnDist computes the average distance between each reconstructed patient record and its K nearest neighbours in the forgetting set (we set K to 10 across all experiments). A lower MI-KnnDist indicates that the reconstructed records better resemble the original records to be forgotten, suggesting higher privacy-leak risks. In contrast, a higher MI-KnnDist value indicates more thorough unlearning and stronger privacy protections.

We have also added OrthoGrad[6](denoted as ORTHO in this work) as a baseline approach. Specifically, ORTHO is a recent unlearning approach that performs per-sample gradient orthogonalisation by projecting forget gradients onto the orthogonal subspace of retain gradients to achieve effective data removal. Although ORTHO adopted gradient orthogonalisation to improve the predictive performance of unlearned models, it does not address algorithmic unfairness arising from unlearning and can, like other baselines, exacerbate healthcare disparities.

Table R1: Ethical-specific algorithmic unfairness of unlearned LSTM models of various machine unlearning methods on the MIMIC-IV dataset. We evaluate three forgetting ratios ("Forg. Ratio" in the table): 1%, 5%, and 10% ("forgetting ratios") of the training set. "0%" denotes the full training set on which the original model is learned. "Retrained" denotes the same model entirely retrained on the remaining set.

	EO-TP ↓ (SD)				EO-FP ↓ (SD)				DP ↓ (SD)			
Forg. Ratio	0%	1%	5%	10%	0%	1%	5%	10%	0%	1%	5%	10%
Original	0.043	-	-	-	0.031	-	-	-	0.045	-	-	-
Retrained	-	0.040(0.006)	0.039(0.008)	0.043(0.016)	-	0.028(0.003)	0.027(0.004)	0.029(0.006)	-	0.038(0.006)	0.044(0.003)	0.042(0.004)
Unlearning Methods												
FU (Ours)	-	**0.041**(0.001)	**0.034**(0.002)	**0.032**(0.001)	-	**0.027**(0.003)	**0.025**(0.002)	**0.022**(0.001)	-	**0.038**(0.002)	**0.036**(0.001)	**0.032**(0.000)
GA	-	0.047(0.004)	0.045(0.006)	0.048(0.004)	-	0.032(0.005)	0.045(0.007)	0.044(0.006)	-	0.046(0.005)	0.056(0.007)	0.050(0.009)
CR	-	0.046(0.008)	0.048(0.007)	0.052(0.020)	-	0.034(0.008)	0.033(0.007)	0.038(0.014)	-	0.046(0.010)	0.041(0.008)	0.049(0.011)
CF	-	0.042(0.007)	0.040(0.003)	0.051(0.007)	-	0.041(0.004)	0.040(0.010)	0.032(0.012)	-	0.054(0.003)	0.052(0.011)	0.042(0.013)
SCRUB	-	0.067(0.024)	0.052(0.002)	0.058(0.017)	-	0.031(0.005)	0.030(0.004)	0.027(0.004)	-	0.043(0.004)	0.042(0.003)	0.040(0.003)
ORTHO	-	0.043(0.008)	0.039(0.005)	0.044(0.010)	-	0.043(0.008)	0.053(0.007)	0.055(0.001)	-	0.055(0.007)	0.064(0.006)	0.065(0.001)

The performance of various methods on the MIMIC-IV dataset can be found in Table R1 and Table R2. As shown in Table R1, the proposed FU consistently achieves better fairness performance than all unlearning baselines across the three metrics, EO-TP, EO-FP, and DP, at forgetting ratios of 1%, 5%, and 10% on the MIMIC-IV dataset. The diagnostic performance (AUROC) and unlearning effectiveness (MIA-AUROC and MI-KnnDist) of FU, as demonstrated in Table R2, generally outperform all baselines on MIMIC-IV. We summarise in Table R3 the DP performance of different unlearning methods across ethnic subgroups, and FU generally achieves more balanced selection rates and thus better unlearning fairness. The new unlearning effectiveness metric MI-KnnDist is summarised

in Table R4. We compare the performance of ORTHO with the proposed FU in Table R7 and Table R8 (“Reply 5”, “Response to Reviewer #1”). We also provide an updated radar plot in Figure R1 to summarise the overall performance, including MIMIC-IV as a new dataset, ORTHO as a new baseline, and MI-KnnDist as a new unlearning metric, in which our FU still achieves the highest overall scores across all evaluation dimensions. Overall, we believe that the additional evaluation results have sufficiently demonstrated the generality of our claims about FU’s potential to improve unlearning fairness. We have correspondingly revised the manuscript to include those results.

Table R2: Diagnostic performance (AUROC) and unlearning effectiveness (MIA-AUROC and MI-KnnDist) of various methods on the MIMIC-IV dataset.

	AUROC ↑ (SD)				MIA-AUROC →0.5 (SD)			MI-KnnDist ↑ (SD)		
Forgetting Ratio	0%	1%	5%	10%	1%	5%	10%	1%	5%	10%
Original	0.870	-	-	-	-	-	-	-	-	-
Retrained	-	0.860 (0.006)	0.859 (0.002)	0.856 (0.004)	0.492 (0.010)	0.512 (0.007)	0.499 (0.002)	12.88 (1.287)	12.71 (0.858)	12.95 (1.910)
Unlearning Methods										
FU (Ours)	-	**0.853** (0.002)	**0.834** (0.000)	**0.824** (0.000)	**0.753** (0.026)	**0.614** (0.017)	**0.517** (0.009)	12.54 (0.525)	**12.61** (0.312)	**12.88** (1.691)
GA	-	0.849 (0.008)	0.815 (0.009)	0.791 (0.007)	0.775 (0.031)	0.686 (0.019)	0.522 (0.023)	12.38 (1.000)	12.20 (0.783)	12.86 (0.929)
CR	-	0.842 (0.015)	0.826 (0.010)	0.791 (0.046)	0.763 (0.021)	0.638 (0.014)	0.522 (0.019)	**12.66** (1.162)	11.97 (0.657)	12.42 (1.473)
CF	-	0.848 (0.007)	0.826 (0.009)	0.764 (0.020)	0.791 (0.019)	0.687 (0.010)	0.454 (0.032)	12.17 (0.625)	12.37 (1.078)	12.51 (0.998)
SCRUB	-	0.847 (0.004)	0.832 (0.003)	0.805 (0.006)	0.789 (0.010)	0.749 (0.033)	0.468 (0.025)	12.05 (1.237)	12.15 (1.060)	12.22 (0.448)
ORTHO	-	0.847 (0.017)	0.826 (0.013)	0.808 (0.019)	0.808 (0.010)	0.701 (0.013)	0.541 (0.019)	12.08 (0.503)	11.94 (0.857)	12.84 (0.491)

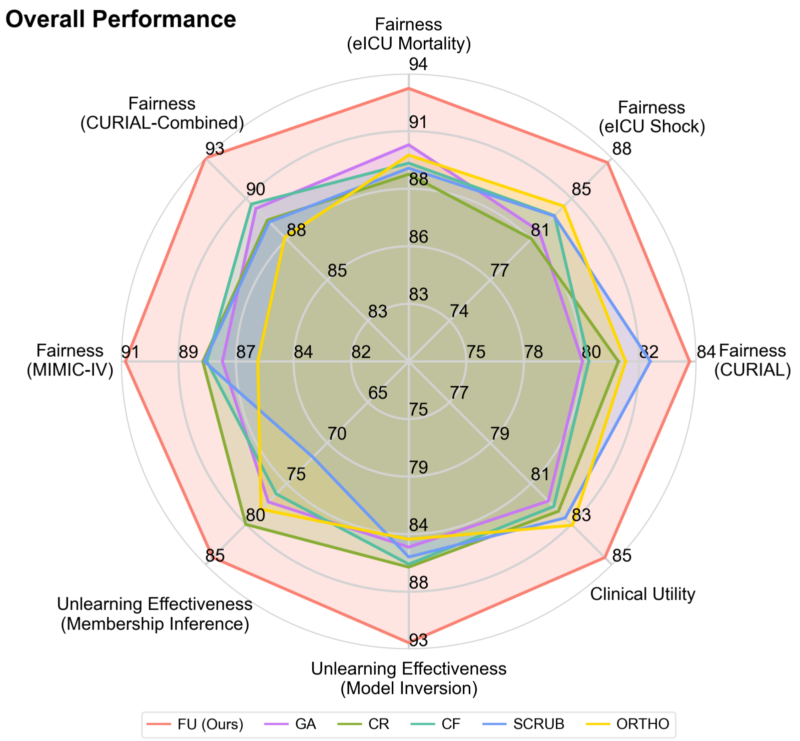

Figure R1: The proposed FU achieves superior performance than prevalent unlearning baselines on clinical utility, unlearning effectiveness, and, more desirably, algorithmic fairness across various real-world medical datasets and clinical tasks. Each radii in the radar plot represents a normalised, overall score of a specific unlearning criterion averaged across the 1%, 5%, and 10% forgetting ratios, which is the higher, the better.

Table R3: Demographic Parity (DP) across CURIAL, CURIAL-Combined, eICU Mortality, and eICU Shock datasets for different unlearning methods. Lower DP values indicate better fairness.

Forgetting Ratio / Unlearning Methods	CURIAL (OUH wave 2) 0%	1%	5%	10%	CURIAL (PUH wave 2) 0%	1%	5%	10%
Original	0.020	-	-	-	0.190	-	-	-
FU (Ours)	-	0.082(0.002)	0.079(0.002)	**0.072**(0.001)	-	**0.036**(0.002)	**0.036**(0.006)	**0.040**(0.006)
GA	-	0.132(0.007)	0.090(0.006)	0.085(0.006)	-	0.048(0.011)	0.049(0.012)	0.050(0.003)
CR	-	0.084(0.005)	0.087(0.009)	0.092(0.007)	-	0.044(0.009)	0.047(0.006)	0.054(0.014)
CF	-	0.092(0.006)	0.087(0.007)	0.080(0.013)	-	0.051(0.017)	0.042(0.005)	0.054(0.005)
SCRUB	-	**0.079**(0.004)	**0.073**(0.004)	0.073(0.002)	-	0.046(0.015)	0.046(0.005)	0.051(0.013)
ORTHO	-	0.098(0.026)	0.078(0.020)	0.076(0.009)	-	0.048(0.014)	0.045(0.005)	0.054(0.010)

Forgetting Ratio / Unlearning Methods	CURIAL (UHB wave 1) 0%	1%	5%	10%	CURIAL (BH wave 2) 0%	1%	5%	10%
Original	0.089	-	-	-	0.058	-	-	-
FU (Ours)	-	**0.024**(0.001)	**0.024**(0.005)	**0.025**(0.009)	-	**0.186**(0.001)	0.180(0.004)	**0.182**(0.000)
GA	-	0.024(0.003)	0.027(0.006)	0.037(0.011)	-	0.194(0.011)	0.166(0.037)	0.189(0.020)
CR	-	0.025(0.002)	0.027(0.001)	0.029(0.002)	-	0.195(0.021)	0.188(0.012)	0.186(0.069)
CF	-	0.028(0.005)	0.036(0.006)	0.035(0.004)	-	0.186(0.028)	0.188(0.012)	0.189(0.008)
SCRUB	-	0.026(0.002)	0.026(0.002)	0.035(0.004)	-	0.189(0.015)	**0.155**(0.052)	0.183(0.007)
ORTHO	-	0.025(0.005)	0.024(0.003)	0.026(0.002)	-	0.188(0.012)	0.186(0.036)	0.183(0.007)

Forgetting Ratio / Unlearning Methods	eICU (Mortality) 0%	1%	5%	10%	eICU (Shock) 0%	1%	5%	10%
Original	0.021	-	-	-	0.052	-	-	-
FU (Ours)	-	**0.019**(0.001)	**0.020**(0.000)	**0.019**(0.001)	-	**0.044**(0.001)	**0.038**(0.001)	**0.027**(0.001)
GA	-	0.020(0.004)	0.037(0.005)	0.030(0.003)	-	0.047(0.010)	0.047(0.011)	0.077(0.014)
CR	-	0.022(0.006)	0.036(0.021)	0.034(0.009)	-	0.079(0.011)	0.062(0.012)	0.059(0.009)
CF	-	0.024(0.006)	0.024(0.004)	0.031(0.006)	-	0.055(0.015)	0.048(0.007)	0.044(0.004)
SCRUB	-	0.023(0.008)	0.027(0.007)	0.027(0.004)	-	0.045(0.007)	0.049(0.006)	0.044(0.012)
ORTHO	-	0.022(0.002)	0.021(0.004)	0.026(0.004)	-	0.053(0.004)	0.049(0.004)	0.041(0.006)

Forgetting Ratio / Unlearning Methods	CURIAL-Combined 0%	1%	5%	10%
Original	0.059	-	-	-
FU (Ours)	-	**0.053**(0.003)	**0.049**(0.003)	**0.046**(0.001)
GA	-	0.061(0.008)	0.083(0.010)	0.052(0.003)
CR	-	0.066(0.011)	0.066(0.016)	0.061(0.015)
CF	-	0.061(0.016)	0.064(0.025)	0.062(0.025)
SCRUB	-	0.073(0.007)	0.071(0.004)	0.069(0.015)
ORTHO	-	0.071(0.007)	0.083(0.010)	0.086(0.012)

Table R4: MI-KnnDist across CURIAL, CURIAL-Combined, eICU Mortality, and eICU Shock datasets for different unlearning methods. Higher MI-KnnDist indicates more thorough forgetting.

	CURIAL (OUH wave 2)			CURIAL-Combined		
Forgetting Ratio	1%	5%	10%	1%	5%	10%
Unlearning Methods						
FU (Ours)	3.67(0.499)	**4.19**(0.169)	**4.19**(0.242)	**2.50**(0.178)	**2.98**(0.139)	**3.01**(0.266)
GA	3.45(0.457)	4.00(0.982)	4.06(0.652)	2.21(0.133)	2.62(0.157)	2.74(0.116)
CR	3.53(0.426)	4.11(1.011)	4.17(0.415)	2.32(0.137)	2.64(0.098)	2.67(0.155)
CF	3.61(0.246)	4.18(0.669)	3.75(0.683)	2.34(0.132)	2.71(0.162)	2.71(0.156)
SCRUB	**3.86**(0.588)	3.63(0.667)	4.00(0.171)	2.21(0.135)	2.64(0.142)	2.88(0.125)
ORTHO	3.52(0.354)	3.41(0.472)	3.60(0.500)	2.28(0.156)	2.62(0.101)	2.62(0.204)

	eICU (Mortality)			eICU (Shock)		
Forgetting Ratio	1%	5%	10%	1%	5%	10%
Unlearning Methods						
FU (Ours)	**17.97**(0.605)	**19.02**(0.453)	**18.68**(0.710)	**5.61**(0.176)	**5.62**(0.125)	**5.66**(0.109)
GA	12.62(0.930)	15.40(0.410)	16.77(0.898)	4.31(0.350)	5.23(0.188)	5.37(0.085)
CR	12.41(0.765)	18.22(0.461)	18.22(0.416)	4.58(0.301)	5.28(0.249)	5.19(0.149)
CF	13.13(1.042)	14.37(0.856)	17.21(0.536)	5.50(0.270)	5.29(0.300)	5.38(0.175)
SCRUB	12.42(0.795)	16.06(1.033)	18.62(0.510)	4.81(0.102)	5.17(0.213)	5.23(0.262)
ORTHO	15.35(0.866)	16.02(0.604)	16.91(0.395)	4.69(0.312)	5.04(0.153)	5.23(0.214)

Comment 3. *The datasets were anonymised. Were there further privacy preservation techniques applied to the datasets? The behaviour of the proposed scheme under different privacy-preserving regimes could be of great interest.*

Reply 3. Data anonymisation[7], as the most commonly used method for protecting patient privacy in clinical practice, is the only privacy preservation technique applied to the datasets. To investigate the effects of different privacy-preserving regimes, we also incorporate differential privacy[8], one of the most prevalent techniques for privacy preservation in deep neural networks (DNNs), and evaluate its effects on our FU method. Specifically, we train a Transformer network[9] (2 self-attention layers, each with 8 self-attention heads; see Supplementary Table 5 for the detailed architecture) on the eICU dataset for the shock prediction task under two settings: with and without the differential privacy strategy. To implement differential privacy during training, we adopt stochastic gradient descent with gradient clipping, with a target privacy budget $(\varepsilon, \delta) = (4.0, 10^{-5})$ and a maximum gradient norm of 1.0. After that, we apply our FU to the two trained models to compare their performance after data removal.

The results are summarised in Table R5, and we can observe that, in general, adding differential privacy provides stronger privacy protection for forgotten data, achieving better MIA-AUROCs (from membership inference attacks) and MI-KnnDists (from model inversion attacks) than without it. This phenomenon aligns with our expectations, as differential privacy, an effective privacy preservation technique, could provide an additional layer of privacy protection for patients to be forgotten when combined with our FU. This observation also demonstrates the compatibility of our FU with other privacy protection techniques. However, we can also observe trade-offs when employing differential privacy: the predictive (AUROC) and fairness (EO-TP, EO-FP, and DP) performance extensively degrade on unlearned models with differential privacy. The compromised performance can be reasonably attributed to the perturbations and noise introduced by differential privacy, which impair the

model's discriminative power and disproportionately affect minority groups.

We can conclude that although adding another privacy-preserving technique, such as differential privacy, could improve the unlearning effectiveness, it will also undermine the model's utility and fairness. Additional computational overhead is also needed for those techniques. Therefore, there is a trade-off to consider. In general, however, the disadvantages of applying additional privacy-preserving techniques seem to outweigh their benefits, as our FU already provides sufficient privacy protection in most cases, and the reduced utility and fairness will be a commonly unacceptable cost. We have added Section "Effects of additional privacy-preservation technique" in Supplementary Note 3 (p. 18) to reflect this discussion.

Table R5: Effects of differential privacy ("DiffPri") on unlearning performance for eICU shock prediction. A transformer network is first trained with and without differential privacy, and FU is applied to the two trained models with 1%, 5%, and 10% unlearning ratios. "FU + DiffPri" indicates that FU is applied to a transformer network trained with differential privacy.

	EO-TP ↓ (SD)			EO-FP ↓ (SD)			DP ↓ (SD)		
Forgetting Ratio	1%	5%	10%	1%	5%	10%	1%	5%	10%
Methods									
FU (Ours)	**0.060**(0.000)	**0.052**(0.001)	**0.049**(0.001)	**0.036**(0.007)	0.034(0.001)	**0.031**(0.001)	**0.031**(0.000)	**0.030**(0.001)	**0.030**(0.005)
FU + DiffPri	0.071(0.004)	0.069(0.000)	0.070(0.000)	0.038(0.003)	**0.032**(0.000)	0.031(0.002)	0.034(0.003)	0.030(0.000)	0.030(0.001)

	AUROC ↑ (SD)			MIA-AUROC →0.5 (SD)			MI-KnnDist ↑ (SD)		
Forgetting Ratio	1%	5%	10%	1%	5%	10%	1%	5%	10%
Methods									
FU (Ours)	**0.859**(0.000)	**0.844**(0.001)	**0.831**(0.001)	0.775(0.019)	0.687(0.010)	0.535(0.022)	5.76(0.223)	5.69(0.300)	5.76(0.127)
FU + DiffPri	0.838(0.000)	0.832(0.000)	0.829(0.000)	**0.702**(0.019)	**0.646**(0.010)	**0.504**(0.022)	**6.48**(0.124)	**6.61**(0.050)	**6.72**(0.143)

Comment 4. *I did not check all the tables providing a summary for datasets. However, the numbers presented in Table 1 do not fully add up, e.g., ethnicity totals do not match to the cohort totals or percentages add up to >100%.*

Reply 4. Thank you for the careful attention. After re-examination, we have spotted three typos in the dataset summary table (Table 1 in the manuscript) that occurred when transferring statistical cohort data to the manuscript: 1). "14,079" (CURIAL, OUH wave 2, White Ethnicity) should be "14,035"; 2). "59,583" (CURIAL, UHB wave 2, White Ethnicity) should be "59,683"; 3). "28,529 " (eICU-mortality, *n* total) should be "30,529". We have also thoroughly verified the accuracy of all statistical data. The revised table is shown in Table R6, and we have revised the manuscript accordingly.

Table R6: Summary population characteristics of CURIAL, CURIAL-Combined, eICU and MIMIC-IV datasets.

CURIAL

Cohort characteristics of ethnicities and ages across four UK NHS trusts. Data is divided into two COVID-19 pandemic waves. Models are trained on positive cases of OUH wave 1 data and OUH pre-pandemic controls. Prospective and external evaluations are performed on the wave 2 data of OUH, PUH, PH, and wave 1 of UHB.

	OUH (pre-pandemic)	OUH (wave 1)	OUH (wave 2)	PUH (wave 2)	UHB (wave 1)	BH (wave 2)
Cohort	1/12/2018-30/11/2019	13/3/2020-29/10/2020	01/11/2020-06/03/2021	01/11/2020-01/03/2021	18/05/2019-08/05/2020	01/01/2021-31/03/2021
n, total	85,607	855	18,499	13,592	95,236	1,863
n, positive	0	855	1,908 (10.3)	1,488 (10.9)	790 (0.8)	210 (11.3)
Ethnicity (%)						
White	69,234 (80.9)	575 (67.3)	14,035 (75.9)	10,205 (75.1)	59,683 (62.7)	1,585 (85.1)
Unknown	10,923 (12.8)	161 (18.9)	3,340 (18.0)	3,086 (22.7)	11,573 (12.2)	$*$
South Asian	2,007 (2.3)	38 (4.4)	369 (2.0)	65 (0.5)	13,883 (14.6)	123 (6.6)
Other	1,432 (1.7)	37 (4.3)	347 (1.9)	96 (0.7)	3,011 (3.2)	46 (2.5)
Black	1,016 (1.2)	29 (3.4)	238 (1.3)	72 (0.5)	4,669 (4.9)	76 (4.1)
Mixed	792 (0.9)	13 (1.5)	126 (0.7)	53 (0.4)	1,944 (2.0)	27 (1.4)
Chinese	203 (0.2)	$*$	44 (0.2)	15 (0.1)	473 (0.5)	$*$
Age (%)						
18-39	21,027 (24.6)	90 (10.5)	3,030 (16.4)	2,558 (18.8)	29,652 (31.1)	389 (21.3)
40-64	25,724 (30.0)	295 (34.5)	5,545 (30)	3,354 (24.7)	32,031 (33.6)	619 (33.8)
$>$65	38,856 (45.4)	470 (55.0)	9,924 (53.6)	7,680 (56.5)	33,553 (35.2)	822 (44.9)

$*$ We merged cases from these subgroups into the "Other" cohort for statistical disclosure controls. Age groups (18-39, 40-64, $>$65) are reported for adult patients with available age information. Patients younger than 18 years in the BH cohort (n = 33) were excluded from all analyses.

CURIAL-Combined

Cohort characteristics across four NHS trusts.

	Hospital (%)				Combined
	OUH	PUH	UHB	BH	
n, total	104,961 (48.7)	13,592 (6.3)	95,236 (44.2)	1,863 (0.9)	215,652
n, positive	2763 (2.6)	1488 (10.9)	790 (0.8)	210 (11.3)	5,251

eICU

Cohort characteristics of ethnicities for the mortality and shock prediction task in eICU.

Task	n, total	Ethnicity(%)					
		Caucasian	Afr. Am.	Hispanic	Asian	Nat. Am.	Other/Unk.
Mortality	30,529	23,655 (77.5)	3,402 (11.1)	1,111 (3.6)	492 (1.6)	-	1,869 (6.1)
Shock	48,288	36,803 (76.2)	5,562 (11.5)	2,254 (4.7)	725 (1.5)	262 (0.5)	2,682 (5.6)

Abbreviations: Afr. Am., African American; Nat. Am., Native American; Other/Unk., Other/Unknown.

MIMIC-IV

Cohort characteristics of ethnicities for the mortality prediction task in MIMIC-IV.

Task	n, total	Ethnicity(%)					
		White	Unknown	Black	Other	Hispanic	Asian
Mortality	54,831	37,118 (67.7)	6,077 (11.1)	5,722 (10.4)	2,320 (4.2)	2,014 (3.7)	1,580 (2.9)

Comment 5a. *Do any of the baseline algorithms attempt to optimise the fairness as well? Given that the latest baseline dates back to 2023 and the publication of unlearning schemes based on gradient orthogonalisation in 2024 and 2025, are there potentially better baselines? Related work should cover the most recent works due to the recent increasing popularity of the topic.*
Comment 5b. *The contribution of the paper and its novelty should be better articulated. Specifically, missing related work published between 2024 and 2025 does not help. There are comparable works using gradient orthogonalization as a fair unlearning approach, e.g., mitigating interference between gradients of forget and remaining set (https://arxiv.org/abs/2503.02312).*

Reply 5. To the best of our knowledge, we are the first to optimise algorithmic fairness during machine unlearning via gradient orthogonalisations, especially in the clinical sector. There are a few recent works on machine unlearning that use orthogonal constraints to address the potential conflicts between the forgetting and learning objectives, thereby improving predictive performance. ORTHO[6] performs per-sample gradient orthogonalisation by projecting forgetting gradients onto the orthogonal subspace of gradients on the remaining set, which is by far the most related work to our FU. However, it emphasises only the predictive performance of unlearned models in automatic speech recognition (ASR) and ignores the crucial dimensionality of algorithmic equality and privacy protections. GDR-GMA[10] adopts a multi-task learning perspective for machine unlearning, rectifying conflicting gradients by projecting them onto orthogonal planes while dynamically adjusting gradient magnitudes. FG-OrIU[11] imposes orthogonal constraints at both feature and gradient levels, dynamically adjusting subspaces during incremental unlearning to prevent knowledge reintroduction and maintain stability between the forgetting and retaining samples. Neither of them, however, attempts to address the algorithmic unfairness that could lead to societal inequalities across patient subgroups, nor to address the AI ethical dilemma between RtbF and fairness.

To better validate the generality of the proposed FU method, we include ORTHO[6], which utilises orthogonalisation to mitigate interference between the gradients of the forget and remaining sets, as a new unlearning baseline. We compare the performance of ORTHO with the proposed FU in Table R7 and Table R8. It can be seen that our FU consistently outperforms ORTHO across all datasets on fairness (EO-TP, EO-FP, and DP), diagnostic performance (AUROC), and unlearning effectiveness (MIA-AUROC and MI-KnnDist). Those results demonstrate the superiority and generality of the proposed FU in real-world clinical tasks, distinguishing it from other orthogonalisation-based methods that consider only independence between the forgetting and retaining sets.

Those results demonstrate the superiority and generality of the proposed FU in real-world clinical tasks, distinguishing it from other orthogonalisation-based methods that consider only independence between the forgetting and retaining sets.

We have revised Section "Results" in the manuscript to include ORHTO as a new baseline and Section "Discussion" (lines 465-479, p. 22) to provide an up-to-date literature review.

Table R7: Ethical-specific algorithmic unfairness between our FU and ORTHO across the 1%, 5% and 10% forgetting ratios on CURIAL, CURIAL-Combined, and eICU (mortality and shock predictions) datasets.

	EO-TP ↓ (SD)			EO-FP ↓ (SD)			DP ↓ (SD)		
Forgetting Ratio	1%	5%	10%	1%	5%	10%	1%	5%	10%
OUH (wave 2)									
FU (Ours)	**0.057**(0.001)	**0.049**(0.001)	**0.046**(0.001)	**0.049**(0.000)	**0.045**(0.001)	**0.044**(0.000)	**0.082**(0.002)	**0.079**(0.002)	**0.072**(0.001)
ORTHO	0.064(0.008)	0.068(0.006)	0.063(0.008)	0.056(0.017)	0.053(0.013)	0.058(0.010)	0.098(0.026)	0.078(0.020)	0.076(0.009)
PUH (wave 2)									
FU (Ours)	**0.077**(0.008)	**0.075**(0.008)	**0.055**(0.013)	**0.046**(0.002)	**0.039**(0.003)	**0.040**(0.004)	**0.036**(0.002)	**0.036**(0.006)	**0.040**(0.006)
ORTHO	0.086(0.010)	0.085(0.005)	0.075(0.011)	0.046(0.002)	0.049(0.010)	0.058(0.015)	0.048(0.014)	0.045(0.005)	0.054(0.010)
UHB (wave 1)									
FU (Ours)	**0.090**(0.001)	0.093(0.005)	**0.087**(0.016)	**0.020**(0.006)	**0.021**(0.002)	**0.021**(0.002)	**0.024**(0.001)	**0.024**(0.005)	**0.025**(0.009)
ORTHO	0.092(0.009)	**0.092**(0.006)	0.087(0.007)	0.024(0.004)	0.023(0.003)	0.025(0.002)	0.025(0.005)	0.024(0.003)	0.026(0.002)
BH (wave 2)									
FU (Ours)	**0.140**(0.009)	**0.098**(0.017)	**0.102**(0.020)	0.151(0.001)	**0.140**(0.005)	**0.124**(0.011)	**0.186**(0.001)	**0.180**(0.004)	**0.182**(0.000)
ORTHO	0.151(0.003)	0.146(0.006)	0.152(0.004)	0.150(0.002)	0.152(0.003)	0.141(0.009)	0.188(0.012)	0.186(0.036)	0.183(0.007)
CURIAL-Combined									
FU (Ours)	**0.010**(0.002)	**0.012**(0.002)	**0.007**(0.002)	**0.017**(0.003)	**0.014**(0.001)	**0.010**(0.001)	**0.053**(0.003)	**0.049**(0.003)	**0.046**(0.001)
ORTHO	0.014(0.002)	0.026(0.007)	0.021(0.003)	0.038(0.012)	0.024(0.007)	0.024(0.006)	0.071(0.007)	0.083(0.010)	0.086(0.012)
eICU (Mortality)									
FU (Ours)	**0.032**(0.001)	**0.039**(0.002)	**0.036**(0.001)	**0.013**(0.001)	0.018(0.001)	**0.013**(0.001)	**0.019**(0.001)	**0.020**(0.000)	**0.019**(0.001)
ORTHO	0.047(0.014)	0.068(0.006)	0.075(0.023)	0.017(0.002)	**0.017**(0.006)	0.022(0.003)	0.022(0.002)	0.021(0.004)	0.026(0.004)
eICU (Shock)									
FU (Ours)	**0.058**(0.001)	**0.054**(0.008)	**0.048**(0.001)	**0.040**(0.001)	**0.040**(0.003)	**0.035**(0.001)	**0.044**(0.001)	**0.038**(0.001)	**0.027**(0.001)
ORTHO	0.075(0.011)	0.077(0.009)	0.094(0.037)	0.051(0.004)	0.046(0.005)	0.037(0.008)	0.053(0.004)	0.049(0.004)	0.041(0.006)

Table R8: Diagnostic performance (AUROC) and unlearning effectiveness (MIA-AUROC and MI-KnnDist) between our FU and ORTHO across the 1%, 5% and 10% forgetting ratios on CURIAL, CURIAL-Combined, and eICU (mortality and shock predictions) datasets.

	AUROC ↑ (SD) (OUH wave 2)			MIA-AUROC →0.5 (SD)			MI-KnnDist ↑ (SD)		
Forgetting Ratio	1%	5%	10%	1%	5%	10%	1%	5%	10%
OUH (wave 2)									
FU (Ours)	**0.877**(0.000)	**0.872**(0.000)	**0.862**(0.000)	0.746(0.019)	**0.657**(0.016)	**0.512**(0.013)	**3.67**(0.499)	**4.19**(0.169)	**4.19**(0.242)
ORTHO	0.874(0.003)	0.844(0.021)	0.831(0.006)	**0.726**(0.024)	0.696(0.014)	0.530(0.016)	3.52(0.354)	3.41(0.472)	3.60(0.500)
PUH (wave 2)									
FU (Ours)	**0.851** (0.000)	**0.842** (0.001)	**0.830** (0.001)	-	-	-	-	-	-
ORTHO	0.851 (0.004)	0.834 (0.003)	0.829 (0.007)	-	-	-	-	-	-
UHB (wave 1)									
FU (Ours)	**0.884** (0.000)	**0.875** (0.000)	**0.865** (0.001)	-	-	-	-	-	-
ORTHO	0.880 (0.005)	0.863 (0.010)	0.846 (0.007)	-	-	-	-	-	-
BH (wave 2)									
FU (Ours)	**0.887** (0.000)	**0.878** (0.000)	**0.863** (0.000)	-	-	-	-	-	-
ORTHO	0.886 (0.002)	0.870 (0.015)	0.857 (0.009)	-	-	-	-	-	-
CURIAL-Combined									
FU (Ours)	**0.890**(0.002)	**0.876**(0.016)	**0.855**(0.001)	**0.659**(0.013)	**0.590**(0.018)	**0.514**(0.007)	**2.50**(0.178)	**2.98**(0.139)	**3.01**(0.266)
ORTHO	0.883(0.010)	0.860(0.013)	0.812(0.009)	0.682(0.011)	0.631(0.016)	0.517(0.010)	2.28(0.156)	2.62(0.101)	2.62(0.204)
eICU (Mortality)									
FU (Ours)	0.844(0.001)	**0.829**(0.002)	**0.811**(0.001)	**0.617**(0.017)	**0.506**(0.012)	0.514(0.011)	**17.97**(0.605)	**19.02**(0.453)	**18.68**(0.710)
ORTHO	**0.845**(0.003)	0.816(0.004)	0.780(0.008)	0.665(0.009)	0.617(0.009)	**0.506**(0.012)	15.35(0.866)	16.02(0.604)	16.91(0.395)
eICU (Shock)									
FU (Ours)	**0.853**(0.000)	**0.841**(0.000)	**0.801**(0.000)	**0.585**(0.019)	**0.492**(0.010)	**0.509**(0.022)	**5.61**(0.176)	**5.62**(0.125)	**5.66**(0.109)
ORTHO	0.845(0.002)	0.826(0.003)	0.784(0.018)	0.632(0.009)	0.584(0.009)	0.512(0.012)	4.69(0.312)	5.04(0.153)	5.23(0.214)

Comment 6. *Authors may like to put supporting evaluations to highlight the computation and time complexity gap between retraining and unlearning for different models and dataset sizes. Considering the additional overhead introduced by the gradient orthogonalisation, at what forgetting rate can retraining be considered as an option?*

Reply 6. We have outlined the computational costs, evaluated in terms of FLOPs and wall-clock time (s), for retraining and our FU across different models and datasets in Table R9. We can see that our FU greatly reduced the computational costs in both FLOPs and wall-clock time compared to retraining. This observation aligns with our anticipations, as our FU requires only a small number of unlearning steps (typically < 100) to remove medical records, whereas retraining requires rerunning the entire training process with potentially thousands of training steps. We also compute the computational overhead of gradient orthogonalisations in our FU, as shown in Table R9. It can be seen that gradient orthogonalisations account for only a small proportion of FU's computations (typically $< 0.1\%$), as they are computed using the Gram-Schmidt algorithm, which adds a low computational overhead. As such, we suggest that the computational cost is not a crucial factor when determining whether to perform retraining or not. Instead, we should comprehensively consider other essential metrics, such as the diagnostic performance and fairness, to decide whether retraining is required.

We have added Section "Evaluations of computational and time complexity" in Supplementary Note 3 (p. 14) to reflect this discussion.

Table R9: Computational costs, measured by FLOPs and wall-clock time in seconds (s), between retraining and our FU on the LSTM network in eICU and the MLP network in CURIAL-Combined across 1%, 5%, and 10% forgetting ratios.

Forgetting Ratio	1%		5%		10%	
Methods	Retrained	FU	Retrained	FU	Retrained	FU
eICU (Shock) & LSTM						
FLOPs ($\times 10^8$)						
Total	1,193,500	98,500	1,143,800	94,400	188,300	16,000
Orthogonalisation (% of Total)	-	6.20 (0.01%)	-	5.94 (0.01%)	-	5.55 (0.03%)
Wall-clock Time (s)	47.25	4.44	45.94	4.10	43.65	3.87
CURIAL-Combined & MLP						
FLOPs ($\times 10^8$)						
Total	2,970	503	2,851	483	2,705	460
Orthogonalisation (% of Total)	-	0.65 (0.13%)	-	0.63 (0.13%)	-	0.60 (0.13%)
Wall-clock Time (s)	132.93	7.27	128.74	6.72	123.59	6.35

Comment 7. *Overall evaluations focus on one round of forgetting, which is not realistic. Forgetting can be handled in batches of 1%, 5%, or 10%, but these will be repeated over specific time frames. How do the presented performance metrics for utility, forgetting and fairness change throughout the forgetting rounds? Would it be possible to talk about a bound or budget at which retraining becomes essential?*

Reply 7. We would first like to clarify that we have already considered the realistic requirements of performing multiple forgetting rounds, and experiments with 5% and 10% forgetting ratios were conducted under an incremental forgetting setting with a 1% gap. For example, for the 5% forgetting ratio, we performed five rounds of unlearning, each round removing 1% data. Similarly, ten rounds

of unlearning, with 1% data per round, were implemented for the 10% forgetting ratio. This setting has been described in Subsection "Incremental forgetting setup" of Section "Methods" in the original manuscript and is also attached below:

"In realistic clinical deployments, patient RtbF requests typically emerge progressively over time rather than arriving as large wholesale batches. This necessitates continuous small-scale unlearning operations rather than infrequent large-scale removals. To reflect this practical scenario, we adopt an incremental forgetting protocol. Specifically, starting from the original model, we remove patient records in consecutive 1% increments, where each subsequent unlearning step operates on the unlearned model resulting from the previous forgetting operation. In this work, we conduct comprehensive experiments spanning 1% to 10% cumulative forgetting ratios, presenting results at three representative forgetting sizes: 1%, 5%, and 10%."

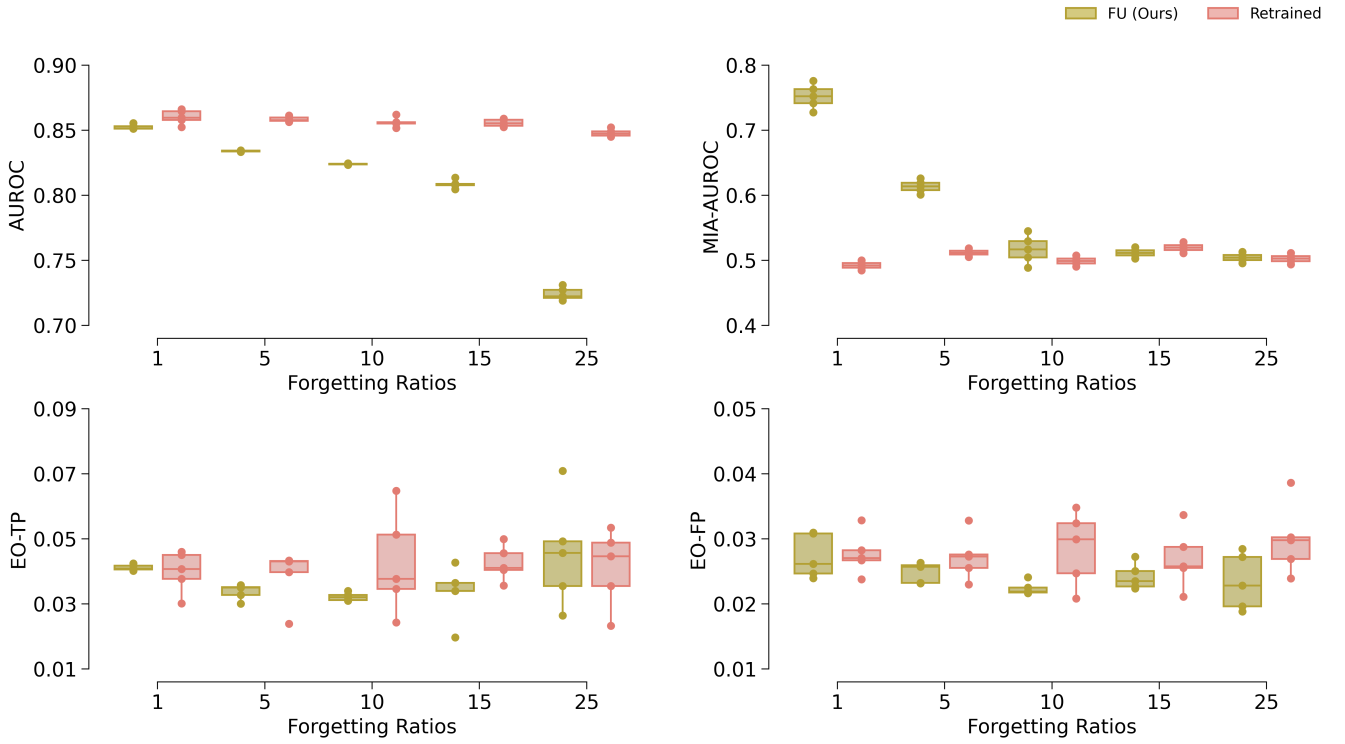

Figure R2: Performance comparisons of unlearned models by our FU and the retrained models across forgetting ratios from 1% to 25%, under the incremental forgetting protocol of consecutive 1% increments, on the mortality prediction task of MIMIC-IV.

To better address the comment, we have thoroughly investigated the performance of FU and retraining across a broader range of forgetting ratios, adopting the same incremental forgetting protocol. Specifically, on the MIMIC-IV dataset, we remove patient records from 1% to 25% forgetting ratios with a 1% increment, and plot the performance of FU and retraining at five forgetting ratios: 1%, 5%, 10%, 15%, 20% and 25% in Figure R2. As shown in the figure, FU exhibits a progressive decline in model utility, with AUROC dropping from 0.85 to 0.73, thereby widening the performance gap relative to the retrained baseline, which shows slightly lower AUROCs. FU's fairness metrics, such as EO-TP and EO-FP, generally exhibit superior performance over retraining across various forgetting ratios. Meanwhile, MIA-AUROC gradually approaches the retraining level (close to 0.5) as the forgetting ratio increases, illustrating more thorough forgetting and better privacy protections.

Those observations imply that model utility can be a more crucial metric for determining whether

a full retraining is necessary, depending on the clinical scenarios encountered upon deployment. For instance, a tiered monitoring system can be adopted on the predictive performance of the unlearning model with the following rules: a 3-5% AUROC drop will trigger a yellow warning flag indicating more intensive monitoring, a 5-10% AUROC drop will incur a red flag for re-investigating the necessity of retraining, and a drop exceeding 10% will launch a retraining to be conducted immediately. Additional flagging systems for fairness or privacy protection metrics can be developed with similar criteria for performance drop and combined to support more comprehensive, real-time monitoring. However, it is worth noting that those criteria are set following empirical rules, and the complexity and challenges of clinical deployments will require those thresholds to be adjusted accordingly and dynamically.

We have correspondingly revised Section "Discussion" in the manuscript (lines 516-529, p. 23) and added Section "Determination of clinical thresholds" in Supplementary Note 3 (p. 22).

Comment 8. *How do the model choice and model size impact the results and practicality of the solution?*

Reply 8. To demonstrate the applicability of our approach to larger or more complex architectures, we first conduct additional evaluations on a 2-layer Transformer model[9] with 2.27M learnable parameters (the detailed architecture is outlined in Supplementary Table 5), in addition to the LSTM network with 20K learnable parameters on the eICU dataset for the mortality and shock prediction task. Table R10 and Table R11 summarise the algorithmic fairness, model utility and unlearning effectiveness for the mortality and shock prediction tasks on the eICU dataset, respectively. As shown in the tables, FU consistently outperforms baselines across algorithmic fairness (EO-TP, EO-FP, and DP), model utility (AUROC), and unlearning effectiveness (MIA-AUROC and MI-KnnDist), for both the LSTM (20K parameters) and Transformer (2.27M) architectures across two clinical tasks. Those results demonstrate that, although we mainly adopt DNN architectures typical of today's clinical AI, such as LSTMs and MLPs, our FU method could also generalise to a more complex and larger DNN architecture, Transformer, which is the core structure of modern deep neural networks.

Additionally, we could observe some interesting findings by comparing the two DNNs' performance. As shown in Table R10 and Table R11, unlearning methods generally achieve higher MIA-AUROC with less desirable unlearning effectiveness for Transformer models than LSTM ones. The model's utility, as measured by AUROCs, is generally better for Transformers than for LSTMs. Those observations indicate that larger and more complex DNNs tend to fit the training data better, i.e., memorise medical records more deeply and thoroughly, which could lead to higher diagnostic performance after unlearning but also makes them more vulnerable to privacy attacks. As such, how to more effectively remove patient information from complex architectures, such as Large Language Models (LLMs), can be an interesting area for future work.

We have accordingly revised Section "Discussion" in the manuscript (lines 578-588, pp. 24-25) and added Section "Scalability to more complex DNNs" in Supplementary Note 3 (p. 15).

Table R10: Performance comparisons of unlearning methods between an LSTM of 20K parameters and a Transformer with 2.27M parameters on the eICU dataset (mortality prediction) across the 1%, 5%, and 10% forgetting ratios.

	EO-TP ↓ (SD)				EO-FP ↓ (SD)				DP ↓ (SD)			
Forg. Ratio	0%	1%	5%	10%	0%	1%	5%	10%	0%	1%	5%	10%
LSTM												
Original	0.041	-	-	-	0.014	-	-	-	0.021	-	-	-
FU (Ours)	-	**0.032** (0.001)	**0.039** (0.002)	**0.036** (0.001)	-	**0.013** (0.001)	0.018 (0.001)	**0.013** (0.001)	-	**0.019** (0.001)	**0.020** (0.000)	**0.019** (0.001)
GA	-	0.043 (0.006)	0.048 (0.010)	0.057 (0.003)	-	0.018 (0.002)	0.019 (0.002)	0.026 (0.003)	-	0.020 (0.004)	0.037 (0.005)	0.030 (0.003)
CR	-	0.044 (0.008)	0.077 (0.023)	0.068 (0.024)	-	0.013 (0.003)	0.024 (0.005)	0.028 (0.010)	-	0.022 (0.006)	0.036 (0.021)	0.034 (0.009)
CF	-	0.048 (0.004)	0.070 (0.005)	0.078 (0.014)	-	0.014 (0.003)	0.020 (0.010)	0.019 (0.003)	-	0.024 (0.006)	0.024 (0.004)	0.031 (0.006)
SCRUB	-	0.047 (0.009)	0.075 (0.014)	0.075 (0.019)	-	0.019 (0.008)	0.023 (0.004)	0.020 (0.005)	-	0.023 (0.008)	0.027 (0.007)	0.027 (0.004)
ORTHO	-	0.047 (0.014)	0.068 (0.006)	0.075 (0.023)	-	0.017 (0.002)	**0.017** (0.006)	0.022 (0.003)	-	0.022 (0.002)	0.021 (0.004)	0.026 (0.004)
Transformer												
Original	0.051	-	-	-	0.018	-	-	-	0.014	-	-	-
FU (Ours)	-	**0.048** (0.002)	**0.042** (0.001)	**0.039** (0.004)	-	**0.016** (0.002)	**0.014** (0.002)	**0.013** (0.001)	-	**0.012** (0.001)	**0.013** (0.002)	**0.012** (0.001)
GA	-	0.055 (0.009)	0.055 (0.004)	0.089 (0.009)	-	0.024 (0.005)	0.044 (0.004)	0.047 (0.002)	-	0.023 (0.004)	0.040 (0.004)	0.048 (0.003)
CR	-	0.048 (0.008)	0.060 (0.030)	0.072 (0.019)	-	0.024 (0.004)	0.024 (0.003)	0.023 (0.006)	-	0.023 (0.005)	0.024 (0.002)	0.022 (0.005)
CF	-	0.059 (0.020)	0.066 (0.020)	0.085 (0.016)	-	0.024 (0.006)	0.045 (0.002)	0.043 (0.003)	-	0.024 (0.003)	0.040 (0.002)	0.045 (0.002)
SCRUB	-	0.050 (0.014)	0.071 (0.010)	0.103 (0.009)	-	0.019 (0.005)	0.019 (0.008)	0.016 (0.006)	-	0.019 (0.006)	0.025 (0.006)	0.024 (0.008)
ORTHO	-	0.053 (0.015)	0.049 (0.028)	0.051 (0.016)	-	0.027 (0.004)	0.026 (0.008)	0.031 (0.010)	-	0.028 (0.007)	0.027 (0.007)	0.029 (0.009)

	AUROC ↑ (SD)				MIA-AUROC →0.5 (SD)			MI-KnnDist ↑ (SD)		
Forg. Ratio	0%	1%	5%	10%	1%	5%	10%	1%	5%	10%
LSTM										
Original	0.853	-	-	-	-	-	-	-	-	-
FU (Ours)	-	0.844 (0.001)	**0.829** (0.002)	**0.811** (0.001)	0.617 (0.017)	**0.506** (0.012)	0.514 (0.011)	**17.97** (0.605)	**19.02** (0.453)	**18.68** (0.710)
GA	-	0.814 (0.004)	0.773 (0.022)	0.747 (0.007)	0.702 (0.022)	0.565 (0.012)	0.463 (0.032)	12.62 (0.930)	15.40 (0.410)	16.77 (0.898)
CR	-	0.833 (0.004)	0.805 (0.009)	0.791 (0.021)	0.665 (0.009)	0.572 (0.007)	0.515 (0.040)	12.41 (0.765)	18.22 (0.461)	18.22 (0.416)
CF	-	**0.848** (0.003)	0.826 (0.003)	0.796 (0.019)	**0.617** (0.013)	0.640 (0.012)	0.484 (0.038)	13.13 (1.042)	14.37 (0.856)	17.21 (0.536)
SCRUB	-	0.840 (0.005)	0.808 (0.008)	0.784 (0.003)	0.744 (0.003)	0.709 (0.003)	0.531 (0.032)	12.42 (0.795)	16.06 (1.033)	18.62 (0.510)
ORTHO	-	0.845 (0.003)	0.816 (0.004)	0.780 (0.008)	0.665 (0.009)	0.617 (0.009)	**0.506** (0.012)	15.35 (0.866)	16.02 (0.604)	16.91 (0.395)
Transformer										
Original	0.874	-	-	-	-	-	-	-	-	-
FU (Ours)	-	0.852 (0.001)	0.835 (0.002)	**0.813** (0.003)	**0.768** (0.013)	0.696 (0.003)	**0.574** (0.004)	18.15 (0.301)	**20.93** (0.705)	**19.63** (0.452)
GA	-	0.851 (0.005)	0.813 (0.006)	0.785 (0.010)	0.807 (0.013)	0.702 (0.009)	0.599 (0.006)	**18.19** (0.241)	15.99 (0.463)	16.68 (0.559)
CR	-	0.847 (0.012)	**0.838** (0.011)	0.795 (0.025)	0.819 (0.011)	0.715 (0.029)	0.582 (0.020)	12.62 (0.910)	18.18 (0.565)	17.71 (0.646)
CF	-	0.847 (0.005)	0.813 (0.001)	0.790 (0.006)	0.786 (0.022)	0.712 (0.012)	0.604 (0.011)	12.74 (0.896)	13.77 (0.623)	15.26 (0.657)
SCRUB	-	**0.854** (0.004)	0.827 (0.007)	0.797 (0.006)	0.794 (0.027)	**0.691** (0.012)	0.617 (0.014)	14.33 (0.354)	18.42 (0.558)	18.19 (0.721)
ORTHO	-	0.840 (0.005)	0.829 (0.012)	0.796 (0.024)	0.797 (0.028)	0.704 (0.014)	0.601 (0.019)	12.40 (0.931)	18.94 (0.615)	14.67 (0.875)

Table R11: Performance comparisons of unlearning methods between an LSTM of 20K parameters and a Transformer of 2.27M parameters on the eICU dataset (shock prediction) across the 1%, 5%, and 10% forgetting ratios.

	EO-TP ↓ (SD)				EO-FP ↓ (SD)				DP ↓ (SD)			
Forg. Ratio	0%	1%	5%	10%	0%	1%	5%	10%	0%	1%	5%	10%
LSTM												
Original	0.070	-	-	-	0.045	-	-	-	0.052	-	-	-
FU (Ours)	-	**0.058** (0.001)	**0.054** (0.008)	**0.048** (0.001)	-	**0.040** (0.001)	**0.040** (0.003)	**0.035** (0.001)	-	**0.044** (0.001)	**0.038** (0.001)	**0.027** (0.001)
GA	-	0.083 (0.008)	0.083 (0.003)	0.097 (0.020)	-	0.044 (0.003)	0.050 (0.005)	0.076 (0.009)	-	0.047 (0.010)	0.047 (0.011)	0.077 (0.014)
CR	-	0.084 (0.004)	0.087 (0.010)	0.088 (0.009)	-	0.050 (0.008)	0.062 (0.007)	0.056 (0.005)	-	0.079 (0.011)	0.062 (0.012)	0.059 (0.009)
CF	-	0.064 (0.016)	0.091 (0.012)	0.093 (0.007)	-	0.045 (0.010)	0.056 (0.019)	0.056 (0.006)	-	0.055 (0.015)	0.048 (0.007)	0.044 (0.004)
SCRUB	-	0.083 (0.016)	0.078 (0.013)	0.089 (0.017)	-	0.049 (0.010)	0.056 (0.008)	0.061 (0.017)	-	0.045 (0.007)	0.049 (0.006)	0.044 (0.012)
ORTHO	-	0.075 (0.011)	0.077 (0.009)	0.094 (0.037)	-	0.051 (0.004)	0.046 (0.005)	0.037 (0.008)	-	0.053 (0.004)	0.049 (0.004)	0.041 (0.006)
Transformer												
Original	0.064	-	-	-	0.038	-	-	-	0.042	-	-	-
FU (Ours)	-	0.060 (0.000)	**0.052** (0.001)	**0.049** (0.001)	-	0.036 (0.007)	**0.034** (0.001)	**0.031** (0.001)	-	**0.031** (0.000)	**0.030** (0.001)	**0.030** (0.005)
GA	-	0.064 (0.005)	0.062 (0.003)	0.096 (0.013)	-	0.046 (0.014)	0.051 (0.007)	0.072 (0.022)	-	0.048 (0.012)	0.046 (0.008)	0.066 (0.018)
CR	-	0.092 (0.026)	0.107 (0.045)	0.081 (0.013)	-	**0.035** (0.009)	0.034 (0.004)	0.037 (0.009)	-	0.032 (0.007)	0.037 (0.005)	0.037 (0.012)
CF	-	**0.052** (0.022)	0.067 (0.008)	0.068 (0.005)	-	0.036 (0.007)	0.048 (0.006)	0.049 (0.012)	-	0.039 (0.008)	0.046 (0.005)	0.046 (0.010)
SCRUB	-	0.085 (0.020)	0.095 (0.024)	0.081 (0.016)	-	0.050 (0.008)	0.050 (0.010)	0.054 (0.008)	-	0.050 (0.008)	0.057 (0.009)	0.061 (0.007)

	AUROC ↑ (SD)				MIA-AUROC →0.5 (SD)			MI-KnnDist ↑ (SD)		
Forg. Ratio	0%	1%	5%	10%	1%	5%	10%	1%	5%	10%
LSTM										
Original	0.862	-	-	-	-	-	-	-	-	-
FU (Ours)	-	**0.853** (0.000)	**0.841** (0.000)	**0.801** (0.000)	**0.585** (0.019)	**0.492** (0.010)	**0.509** (0.022)	**5.61** (0.176)	**5.62** (0.125)	**5.66** (0.109)
GA	-	0.845 (0.002)	0.824 (0.029)	0.749 (0.038)	0.639 (0.018)	0.544 (0.017)	0.511 (0.031)	4.31 (0.350)	5.23 (0.188)	5.37 (0.085)
CR	-	0.852 (0.002)	0.824 (0.025)	0.788 (0.036)	0.598 (0.013)	0.525 (0.008)	0.517 (0.035)	4.58 (0.301)	5.28 (0.249)	5.19 (0.149)
CF	-	0.846 (0.006)	0.822 (0.002)	0.790 (0.008)	0.614 (0.011)	0.642 (0.012)	0.484 (0.039)	5.50 (0.270)	5.29 (0.300)	5.38 (0.175)
SCRUB	-	0.842 (0.002)	0.806 (0.004)	0.773 (0.010)	0.784 (0.003)	0.680 (0.003)	0.510 (0.032)	4.81 (0.102)	5.17 (0.213)	5.23 (0.262)
ORTHO	-	0.845 (0.002)	0.826 (0.003)	0.784 (0.018)	0.632 (0.009)	0.584 (0.009)	0.512 (0.012)	4.69 (0.312)	5.04 (0.153)	5.23 (0.214)
Transformer										
Original	0.874	-	-	-	-	-	-	-	-	-
FU (Ours)	-	**0.859** (0.000)	**0.844** (0.001)	**0.831** (0.001)	**0.687** (0.015)	**0.608** (0.025)	0.511 (0.009)	**5.76** (0.223)	**5.69** (0.300)	**5.76** (0.127)
GA	-	0.853 (0.012)	0.834 (0.005)	0.735 (0.006)	0.706 (0.032)	0.627 (0.020)	0.545 (0.016)	5.41 (0.131)	4.48 (0.190)	4.68 (0.180)
CR	-	0.853 (0.009)	0.828 (0.009)	0.800 (0.034)	0.716 (0.013)	0.664 (0.014)	0.570 (0.012)	4.51 (0.075)	5.05 (0.105)	5.46 (0.088)
CF	-	0.848 (0.007)	0.811 (0.001)	0.803 (0.015)	0.737 (0.026)	0.646 (0.020)	**0.498** (0.017)	4.84 (0.063)	5.50 (0.120)	5.36 (0.150)
SCRUB	-	0.849 (0.011)	0.826 (0.008)	0.806 (0.006)	0.747 (0.019)	0.674 (0.022)	0.538 (0.010)	5.00 (0.155)	5.04 (0.164)	5.59 (0.265)
ORTHO	-	0.845 (0.009)	0.823 (0.008)	0.801 (0.010)	0.725 (0.014)	0.664 (0.036)	0.524 (0.012)	3.99 (0.157)	5.49 (0.131)	5.12 (0.174)

Response to Reviewer #2

Comment 1a. *The manuscript tackles a very timely and critical challenge in AI for health applications on how to implement RtbF for patient data in deployed machine learning models, while maintaining fairness, biases detection and utility. The authors focus specifically on the risks that standard machine unlearning methods can exacerbate algorithmic unfairness, introducing "fair unlearning" approach that enforces orthogonality between unlearning, utility, and fairness objectives. From the privacy perspective, the paper adresses consistentily this angle. The use of membership inference attacks to quantitatively assess whether patient data has truly been "forgotten" is reasonable and well executed. The results demonstrate that their FU framework achieves MIA-AUROC scores close to .5, suggesting effective removal of sensitive records and therefore strong patient data privacy.*
However, it's worth noting that privacy evaluation is limited to this recognized standard, but not exhaustive. Other plausible attack vectors, such as model inversion or attribute inference, are not addressed. The authors could strengthen their privacy claims by at least acknowledging these limitations and discussing the complexities of real-world deployments (for example, tracking and managing unlearning requests in federated settings, or the cumulative risk of sequential unlearning).
Comment 1b. *For improvements, I'd suggest the following: 1. broaden the privacy discussion to include more attack types and practical deployment challenges;*

Reply 1. Thank you for the comment. We have added an additional privacy-attacking paradigm, the model inversion attack (MI), to evaluate the performance of privacy protections across various unlearning methods. Specifically, MI aims to reconstruct the training data by querying or analysing the model outputs, and we follow the MI attack implementation in the prior work[4] to assess the privacy protections of patients' data to be forgotten. The unlearning effectiveness under the MI attack is evaluated by the K-Nearest Neighbour Distance[5], denoted as MI-KnnDist. MI-KnnDist computes the average distance between each reconstructed patient record and its K nearest neighbours in the forgetting set (we set K to 10 across all experiments). A lower MI-KnnDist indicates that the reconstructed records better resemble the original records to be forgotten, suggesting higher privacy-leak risks. In contrast, a higher MI-KnnDist value indicates more thorough unlearning and stronger privacy protections.

Moreover, we additionally perform evaluations on a new real-world healthcare dataset, Medical Information Mart for Intensive Care IV (MIMIC-IV)[1], to assess the privacy-attacking results and other metrics. The MIMIC-IV dataset contains de-identified electronic health records (EHRs) from patients admitted to intensive care units (ICUs) at Beth Israel Deaconess Medical Centre in Boston, Massachusetts, between 2008 and 2019. We generally follow the existing protocol[2] to curate and preprocess 54,831 EHRs (see Supplementary Note 1 for details) collected during the first 24 hours after ICU admission into time-series data. The clinical task is to predict in-hospital mortality. We split all the 54,831 records into an 80:20 ratio for training and testing, and we train a long short-term memory (LSTM) network to evaluate the performance of various unlearning methods. We have also added ORTHO[6], a recent unlearning approach, as a baseline approach.

We report the privacy protection (MIA-AUROC and MI-KnnDist) and predictive performance (AUROC) of various unlearning methods on the CURIAL, CURIAL-Combined, eICU, and MIMIC-IV datasets in Tables R12, R13, R14, and Table R15, respectively. As shown in these tables, the proposed FU provides the best overall privacy protection for patients' forgotten data across all datasets under both privacy attack paradigms, MIA and MI. This observation indicates that our unlearning algorithm has achieved the most thorough record removals, which could provide satisfying privacy protection against

other plausible attack vectors. More favourably, our FU can still guide the unlearned model to achieve the generally highest diagnostic AUROC scores, illustrating the superiority of our fair unlearning mechanism for clinical utilities. We have also updated the radar plot of overall performance evaluations in Figure R3 to include MI-KnnDist as a new unlearning metric, MIMIC-IV as a new dataset, and ORTHO as a new baseline, in which our FU still achieves the highest overall scores across all evaluation dimensions.

Although we have already demonstrated the unlearning effectiveness of our FU method using two typical privacy-attacking schemes, i.e., membership inference and model inversion, we would like to note that there are still multiple other ways of attempting to extract sensitive information from a DNN model, such as property inference[12,13] and model extraction attacks[14,15]. Due to the complexity and vastness of existing attack methods, as well as the emergence of new variants, we are unable to perform an exhaustive evaluation of the privacy preservation capabilities of unlearning methods across all plausible privacy-attacking paradigms, which is a potential limitation of this work. Moreover, the real-world deployment of the unelarning framework will also raise additional, challenging privacy concerns and beyond. For example, the federated learning scenario[16,17] will pose issues in the management and implementation of unlearning requests. With medical records stored in multiple remote devices, the unlearning requests will need to be propagated from local machines to the central server for a complete and effective data removal. Adversaries may attempt to extract sensitive information from remote devices with local AI models, the central server with the global model, as well as the network communication process, resulting in various privacy attack patterns with associated challenges. How to perform unlearning in a collaborative learning environment with adequate privacy preservation can be an interesting topic with practical clinical value for future research. In real-world applications, potential privacy leak risks can also accumulate as multiple batches of forgetting requests are processed in sequence. Multiple unlearning procedures will typically yield multiple opportunities for privacy attacks, representing a greater risk of sensitive information leak than a single one. An adversary may adopt the model output differences across the sequential unlearning operations to infer the removed records. As such, developing a practical guide on how to eliminate privacy leaks comprehensively and effectively can be a crucial future work in real-world deployments of unlearning methods.

We have thoroughly revised Section “Results” in the manuscript to include MI as a new privacy attack paradigm, MIMIC-IV as a new dataset, and ORTHO as a new unlearning baseline. We have also revised Section “Discussion” in the manuscript (lines 560-577, p.24) to discuss real-world deployment challenges.

Table R12: Diagnostic performance (AUROC) and unlearning effectiveness (MIA-AUROC and MI-KnnDist) of various methods on the four UK NHS trusts of CURIAL, organised by prospective and external evaluations. Note that MIA-AUROC and MI-KnnDist are evaluated by privacy attacks on the forgetting sets from the training data.

Prospective evaluation on OUH

	AUROC ↑ (SD) (OUH wave 2)				MIA-AUROC →0.5 (SD)			MI-KnnDist ↑ (SD)		
Forgetting Ratio	0%	1%	5%	10%	1%	5%	10%	1%	5%	10%
Original	0.880	-	-	-	-	-	-	-	-	-
Retrained	-	0.876 (0.001)	0.876 (0.001)	0.875 (0.002)	0.521 (0.002)	0.503 (0.002)	0.511 (0.002)	3.73 (0.331)	4.28 (0.443)	4.33 (0.750)
Unlearning Methods										
FU (Ours)	-	0.877 (0.000)	**0.872** (0.000)	**0.862** (0.000)	0.746 (0.019)	**0.657** (0.016)	**0.512** (0.013)	3.67 (0.499)	**4.19** (0.169)	**4.19** (0.242)
GA	-	**0.879** (0.001)	0.858 (0.006)	0.839 (0.003)	0.801 (0.010)	0.707 (0.026)	0.528 (0.013)	3.45 (0.457)	4.00 (0.982)	4.06 (0.652)
CR	-	0.876 (0.003)	0.851 (0.006)	0.840 (0.021)	0.767 (0.015)	0.671 (0.028)	0.534 (0.010)	3.53 (0.426)	4.11 (1.011)	4.17 (0.415)
CF	-	0.874 (0.004)	0.816 (0.012)	0.802 (0.011)	0.795 (0.010)	0.712 (0.014)	0.513 (0.034)	3.61 (0.246)	4.18 (0.669)	3.75 (0.683)
SCRUB	-	0.869 (0.001)	0.834 (0.005)	0.799 (0.005)	0.800 (0.022)	0.741 (0.025)	0.529 (0.019)	**3.86** (0.588)	3.63 (0.667)	4.00 (0.171)
ORTHO	-	0.874 (0.003)	0.844 (0.021)	0.831 (0.006)	**0.726** (0.024)	0.696 (0.014)	0.530 (0.016)	3.52 (0.354)	3.41 (0.472)	3.60 (0.500)

External evaluations

	AUROC ↑ (SD) (PUH wave 2)				AUROC ↑ (SD) (UHB wave 1)				AUROC ↑ (SD) (BH wave 2)			
Forgetting Ratio	0%	1%	5%	10%	0%	1%	5%	10%	0%	1%	5%	10%
Original	0.856	-	-	-	0.885	-	-	-	0.890	-	-	-
Retrained	-	0.857 (0.002)	0.856 (0.001)	0.855 (0.002)	-	0.884 (0.002)	0.884 (0.004)	0.883 (0.004)	-	0.895 (0.002)	0.894 (0.003)	0.894 (0.004)
Unlearning Methods												
FU (Ours)	-	**0.851** (0.000)	**0.842** (0.001)	**0.830** (0.001)	-	**0.884** (0.000)	**0.875** (0.000)	**0.865** (0.001)	-	**0.887** (0.000)	**0.878** (0.000)	**0.863** (0.000)
GA	-	0.851 (0.001)	0.827 (0.005)	0.808 (0.002)	-	0.881 (0.001)	0.866 (0.004)	0.847 (0.003)	-	0.886 (0.003)	0.864 (0.007)	0.834 (0.003)
CR	-	0.851 (0.004)	0.824 (0.008)	0.810 (0.027)	-	0.879 (0.006)	0.855 (0.011)	0.846 (0.025)	-	0.888 (0.002)	0.871 (0.004)	0.858 (0.018)
CF	-	0.849 (0.002)	0.785 (0.013)	0.771 (0.012)	-	0.878 (0.004)	0.815 (0.022)	0.803 (0.033)	-	0.878 (0.007)	0.809 (0.015)	0.796 (0.011)
SCRUB	-	0.847 (0.001)	0.816 (0.007)	0.781 (0.010)	-	0.869 (0.003)	0.826 (0.008)	0.785 (0.012)	-	0.875 (0.004)	0.840 (0.007)	0.790 (0.012)
ORTHO	-	0.851 (0.004)	0.834 (0.003)	0.829 (0.007)	-	0.880 (0.005)	0.863 (0.010)	0.846 (0.007)	-	0.886 (0.002)	0.870 (0.015)	0.857 (0.009)

Table R13: Diagnostic performance (AUROC) and unlearning effectiveness (MIA-AUROC and MI-KnnDist) of various methods on the CURIAL-Combined dataset.

	AUROC ↑ (SD)				MIA-AUROC →0.5 (SD)			MI-KnnDist ↑ (SD)		
Forgetting Ratio	0%	1%	5%	10%	1%	5%	10%	1%	5%	10%
Original	0.907	-	-	-	-	-	-	-	-	-
Retrained	-	0.904 (0.001)	0.904 (0.001)	0.905 (0.001)	0.527 (0.021)	0.521 (0.026)	0.518 (0.014)	3.22 (0.109)	3.31 (0.089)	3.43 (0.079)
Unlearning Methods										
FU (Ours)	-	0.890 (0.002)	**0.876** (0.016)	**0.855** (0.001)	**0.659** (0.013)	**0.590** (0.018)	**0.514** (0.007)	**2.50** (0.178)	**2.98** (0.139)	**3.01** (0.266)
GA	-	**0.892** (0.003)	0.873 (0.008)	0.751 (0.031)	0.708 (0.021)	0.644 (0.026)	0.564 (0.014)	2.21 (0.133)	2.62 (0.157)	2.74 (0.116)
CR	-	0.876 (0.013)	0.819 (0.075)	0.761 (0.055)	0.733 (0.012)	0.620 (0.007)	0.532 (0.019)	2.32 (0.137)	2.64 (0.098)	2.67 (0.155)
CF	-	0.889 (0.008)	0.802 (0.012)	0.792 (0.015)	0.731 (0.027)	0.681 (0.027)	0.529 (0.014)	2.34 (0.132)	2.71 (0.162)	2.71 (0.156)
SCRUB	-	0.887 (0.004)	0.863 (0.005)	0.837 (0.004)	0.716 (0.019)	0.639 (0.017)	0.537 (0.014)	2.21 (0.135)	2.64 (0.142)	2.88 (0.125)
ORTHO	-	0.883 (0.010)	0.860 (0.013)	0.812 (0.009)	0.682 (0.011)	0.631 (0.016)	0.517 (0.010)	2.28 (0.156)	2.62 (0.101)	2.62 (0.204)

Table R14: Diagnostic performance (AUROC) and unlearning effectiveness (MIA-AUROC and MI-KnnDist) of various methods on the mortality and shock prediction tasks of the eICU dataset.

		AUROC ↑ (SD)			MIA-AUROC →0.5 (SD)			MI-KnnDist ↑ (SD)		
Forgetting Ratio	0%	1%	5%	10%	1%	5%	10%	1%	5%	10%
Mortality										
Original	0.853	-	-	-	-	-	-	-	-	-
Retrained	-	0.847(0.003)	0.847(0.002)	0.847(0.002)	0.504(0.021)	0.483(0.020)	0.503(0.014)	20.80(0.249)	20.98(0.434)	21.29(0.283)
Unlearning Methods										
FU (Ours)	-	0.844(0.001)	**0.829**(0.002)	**0.811**(0.001)	0.617(0.017)	**0.506**(0.012)	0.514(0.011)	**17.97**(0.605)	**19.02**(0.453)	**18.68**(0.710)
GA	-	0.814(0.004)	0.773(0.022)	0.747(0.007)	0.702(0.022)	0.565(0.012)	0.463(0.032)	12.62(0.930)	15.40(0.410)	16.77(0.898)
CR	-	0.833(0.004)	0.805(0.009)	0.791(0.021)	0.665(0.009)	0.572(0.007)	0.515(0.040)	12.41(0.765)	18.22(0.461)	18.22(0.416)
CF	-	**0.848**(0.003)	0.826(0.003)	0.796(0.019)	**0.617**(0.013)	0.640(0.012)	0.484(0.038)	13.13(1.042)	14.37(0.856)	17.21(0.536)
SCRUB	-	0.840(0.005)	0.808(0.008)	0.784(0.003)	0.744(0.003)	0.709(0.003)	0.531(0.032)	12.42(0.795)	16.06(1.033)	18.62(0.510)
ORTHO	-	0.845(0.003)	0.816(0.004)	0.780(0.008)	0.665(0.009)	0.617(0.009)	**0.506**(0.012)	15.35(0.866)	16.02(0.604)	16.91(0.395)
Shock										
Original	0.862	-	-	-	-	-	-	-	-	-
Retrained	-	0.859(0.002)	0.858(0.001)	0.856(0.002)	0.503(0.021)	0.479(0.020)	0.504(0.014)	6.25(0.067)	6.07(0.149)	6.19(0.119)
Unlearning Methods										
FU (Ours)	-	**0.853**(0.000)	**0.841**(0.000)	**0.801**(0.000)	**0.585**(0.019)	**0.492**(0.010)	**0.509**(0.022)	**5.61**(0.176)	**5.62**(0.125)	**5.66**(0.109)
GA	-	0.845(0.002)	0.824(0.029)	0.749(0.038)	0.639(0.018)	0.544(0.017)	0.511(0.031)	4.31(0.350)	5.23(0.188)	5.37(0.085)
CR	-	0.852(0.002)	0.824(0.025)	0.788(0.036)	0.598(0.013)	0.525(0.008)	0.517(0.035)	4.58(0.301)	5.28(0.249)	5.19(0.149)
CF	-	0.846(0.006)	0.822(0.002)	0.790(0.008)	0.614(0.011)	0.642(0.012)	0.484(0.039)	5.50(0.270)	5.29(0.300)	5.38(0.175)
SCRUB	-	0.842(0.002)	0.806(0.004)	0.773(0.010)	0.784(0.003)	0.680(0.003)	0.510(0.032)	4.81(0.102)	5.17(0.213)	5.23(0.262)
ORTHO	-	0.845(0.002)	0.826(0.003)	0.784(0.018)	0.632(0.009)	0.584(0.009)	0.512(0.012)	4.69(0.312)	5.04(0.153)	5.23(0.214)

Table R15: Diagnostic performance (AUROC) and unlearning effectiveness (MIA-AUROC and MI-KnnDist) of various methods on the MIMIC-IV dataset.

		AUROC ↑ (SD)			MIA-AUROC →0.5 (SD)			MI-KnnDist ↑ (SD)		
Forgetting Ratio	0%	1%	5%	10%	1%	5%	10%	1%	5%	10%
Original	0.870	-	-	-	-	-	-	-	-	-
Retrained	-	0.860(0.006)	0.859(0.002)	0.856(0.004)	0.492(0.010)	0.512(0.007)	0.499(0.002)	12.88(1.287)	12.71(0.858)	12.95(1.910)
Unlearning Methods										
FU (Ours)	-	**0.853**(0.002)	**0.834**(0.000)	**0.824**(0.000)	**0.753**(0.026)	**0.614**(0.017)	**0.517**(0.009)	12.54(0.525)	**12.61**(0.312)	**12.88**(1.691)
GA	-	0.849(0.008)	0.815(0.009)	0.791(0.007)	0.775(0.031)	0.686(0.019)	0.522(0.023)	12.38(1.000)	12.20(0.783)	12.86(0.929)
CR	-	0.842(0.015)	0.826(0.010)	0.791(0.046)	0.763(0.021)	0.638(0.014)	0.522(0.019)	**12.66**(1.162)	11.97(0.657)	12.42(1.473)
CF	-	0.848(0.007)	0.826(0.009)	0.764(0.020)	0.791(0.019)	0.687(0.010)	0.454(0.032)	12.17(0.625)	12.37(1.078)	12.51(0.998)
SCRUB	-	0.847(0.004)	0.832(0.003)	0.805(0.006)	0.789(0.010)	0.749(0.033)	0.468(0.025)	12.05(1.237)	12.15(1.060)	12.22(0.448)
ORTHO	-	0.847(0.017)	0.826(0.013)	0.808(0.019)	0.808(0.010)	0.701(0.013)	0.541(0.019)	12.08(0.503)	11.94(0.857)	12.84(0.491)

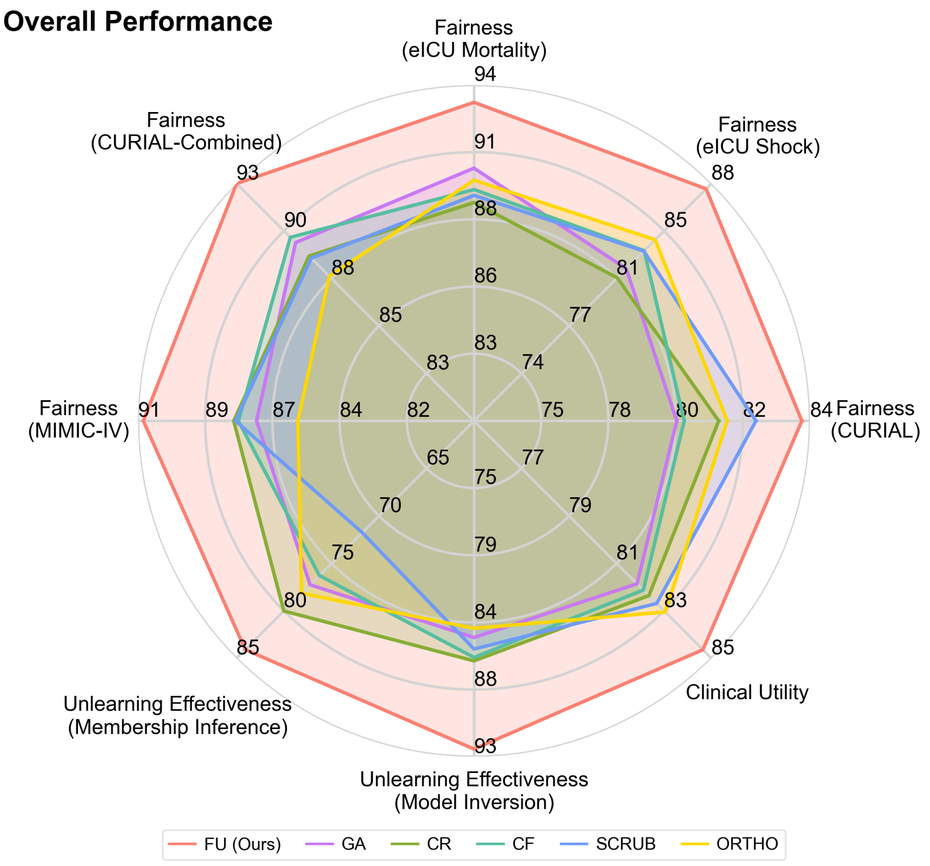

Figure R3: The proposed FU achieves superior performance than prevalent unlearning baselines on clinical utility, unlearning effectiveness, and, more desirably, algorithmic fairness across various real-world medical datasets and clinical tasks. Each radii in the radar plot represents a normalised, overall score of a specific unlearning criterion averaged across the 1%, 5%, and 10% forgetting ratios, which is the higher, the better.

Comment 2a. *On the transparency and explainability angle, the manuscript offers strong contribution. The use of equalised odds as a fairness metric is transparent and interpretable for non-technical stakeholders, and the data visualisation set presented are informative. The "Algorithmic insights" section gives a rare, welcome look into why their approach works, which can foster trust among health professionals.*
However, the clinical explainability of model decisions, particularly post-unlearning, is not explored as it deserves. The paper doesn't show, for instance, whether feature importances or decision logic change after unlearning. Health professionals may want to know how the model's "thinking" shifts, especially if the system is being actively modified in production. Making this point clearer would bring a more consistent explainability aspect for the proposed approach.
Comment 2b. *3. provide some analysis of post-unlearning model explainability for individual predictions.*

Reply 2. We adopt Shapley Additive exPlanations (SHAP)[18] to illustrate the effects of individual features on the model predictions for COVID-19 infections, before and after unlearning 10% training data with our FU, on the 1024 patient records of the OUH wave 2 cohort. The SHAP summary beeswarm plots for the top 15 most important features between the original and unlearned model are shown in Figure R4 a-b. We can see that the unlearning operation does not significantly alter the clinical explainability of model predictions. For example, "Haemoglobin" and "Haematocrit" remained the

two most important clinical variables for determining infection status, while the effects of “Estimated Glomerular Filtration Rate”, though ranked higher after forgetting, still exhibit a generally similar trend as the original model predictions. No substantial changes in explainability are observed in other top influential variables, despite some ranking differences.

We further investigate the five clinical variables with the most significant ranking variants after unlearning: “Eosinophil Count”, “C-reative protein”, “Estimated Glomerular Filtration Rate”, “Respiratory Rate”, and “Albumin”, by plotting their SHAP dependence plots in Figure R4 c-d. As can be seen from the plots, despite slight or moderate changes in several data points, removing medical records using our FU does not significantly alter the overall SHAP dependence trends of the clinical variables. These pieces of evidence imply that our FU algorithm will not significantly alter or impair clinical explainability, and that post-unlearning models could still provide feature importance explanations consistent with the original models to support clinical decision-making. We have revised Section “Results” in the manuscript (lines 403-420, pp. 18-19; Figure 8, p. 21) correspondingly.

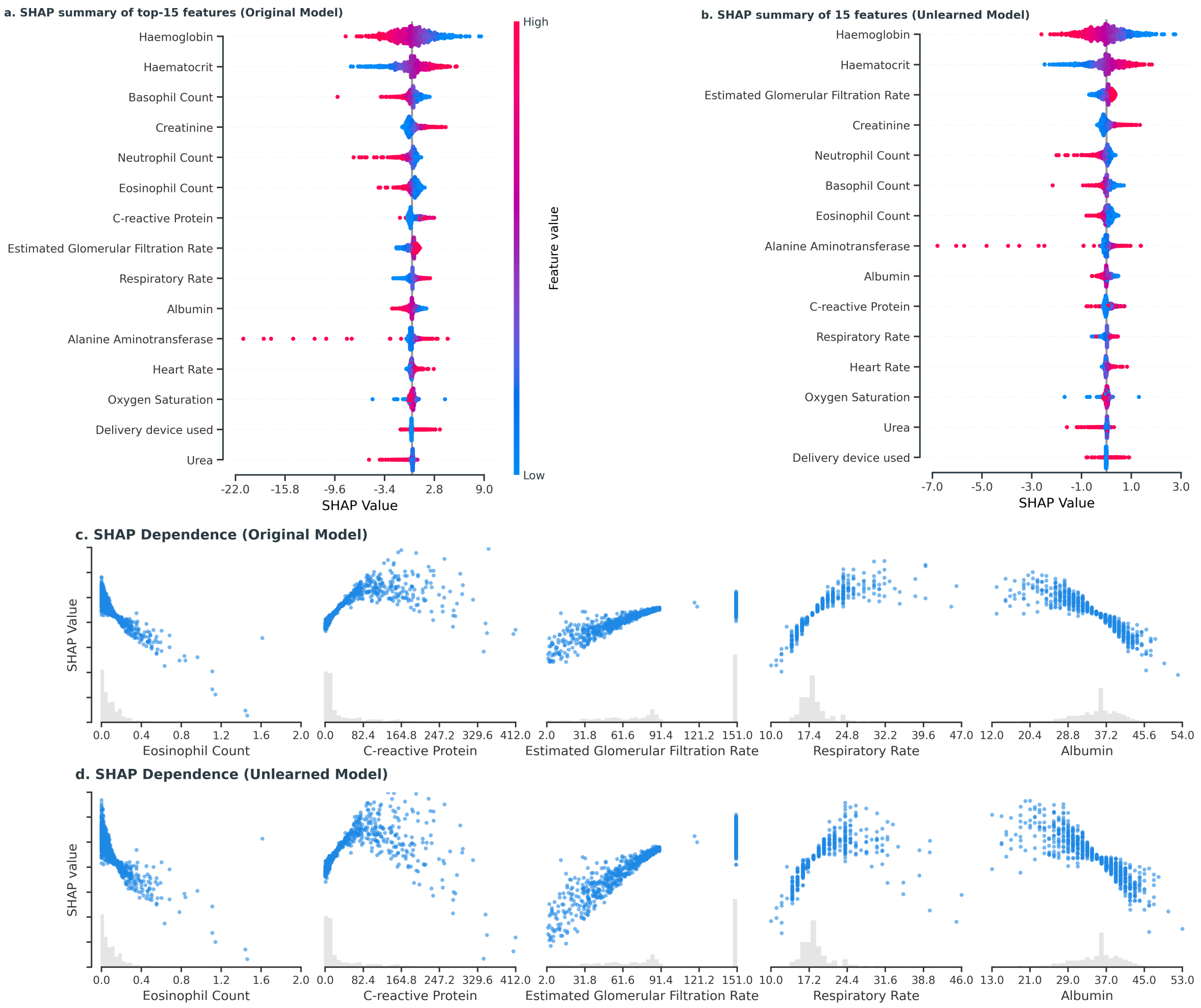

Figure R4: SHAP-based explainability analyses for the original and unlearned models for predicting COVID-19 infections on 1024 patient records of the OUH wave 2 cohort. Each dot represents a single patient record, and positive SHAP values reflect a change in the model’s prediction towards a positive COVID-19 outcome. **a-b**. SHAP summary beeswarm plots for the original model and the unlearned model. Variables are ordered by the mean absolute SHAP value, and the top 15 ones are displayed. **c-d**. SHAP dependence plots for the top-5 variables with the most significant ranking variants after unlearning, with points coloured by raw feature values.

Comment 3a. *On another aspect, fairness is assessed only along single dimensions (ethnicity, hospital), whereas intersectional unfairness—often where real-world disparities emerge—remains unaddressed. Similarly, the performance-fairness trade-off is recognized, but the paper could suggest practical thresholds for clinical deployment, which could be very useful for practitioners.*
Comment 3b. *Furthermore, intersectional fairness and explicit clinical thresholds for "acceptable" drops in model performance would help health systems make better informed decisions about deploying and maintaining these models.*

Reply 3. **Intersectional fairness**: Thanks for your comments. We agree that the capacity to address intersectional unfairness is also critical for clinical AI towards mitigating real-world disparities, an issue mostly overlooked in prior works on clinical algorithmic fairness. As such, we additionally evaluate the performance of various unlearning methods against intersectional unfairness on the CURIAL dataset by jointly stratifying patients by ethnicity and age groups. The cohort statistical information is summarised in Table R16, and this stratification yields a total of 21 ethnicity-age subgroups (e.g., whites aged 40-64, Chinese aged 65+, etc.), across which the intersectional unfairness is evaluated. Similar to our prior experiments on the CURIAL dataset, we first train an MLP model on OUH wave 1 data and OUH pre-pandemic controls, and we evaluate the unlearned model on the wave 2 data of OUH, PUH, PH, and wave 1 of UHB.

Moreover, we employ Demographic Parity (DP)[3] as an additional fairness metric to provide a more comprehensive evaluation. DP measures differences in the positive prediction rates across demographic groups, concentrating on selection-rate disparities independent of ground-truth labels and thus effectively complementing the adopted Equalised Odds (EO-TPs and EO-FPs). We compute DP as the standard deviation of positive prediction rates across sensitive subpopulations; lower DP indicates less disparities in selection rates and thus better fairness.

Table R17 reports the intersectional fairness results (EO-TP, EO-FP, and DP) across the four NHS trust cohorts. Compared to other baseline methods, our FU consistently and effectively mitigate intersectional disparities across the ethnicity-age subgroups on the four NHS trust cohorts. More favourably, as shown in Table R18, our FU still achieves superior predictive performance (higher AUROCs) than all unlearning baselines across the four cohorts, and it also more thoroughly removes patient information from the forgetting set, as evidenced by both higher MIA-AUROC and MI-KnnDist values. As such, we have shown that our FU can also generalise to the scenarios of intersectional unfairness, thereby increasing its potential for real-world applications.

We have correspondingly revised Section "Discussion" in the manuscript (lines 542-552, p. 24) and added Section "Mitigation of intersectional unfairness" in Supplementary Note 3 (pp. 19-21).

Table R16: Summary population characteristics of CURIAL dataset. Cohort characteristics across ethnicities and four UK NHS trusts. Data is divided into two COVID-19 pandemic waves. Models are trained on positive cases of OUH wave 1 data and OUH pre-pandemic controls. Prospective and external evaluations are performed on the wave 2 data of OUH, PUH, PH, and wave 1 of UHB.

	OUH (pre-pandemic)	OUH (wave 1)	OUH (wave 2)	PUH (wave 2)	UHB (wave 1)	BH (wave 2)
Cohort	1/12/2018-30/11/2019	13/3/2020-29/10/2020	01/11/2020-06/03/2021	01/11/2020-01/03/2021	18/05/2019-08/05/2020	01/01/2021-31/03/2021
n, total	85,607	855	18,499	13,592	95,236	1,863
n, positive	0	855	1,908 (10.3)	1,488 (10.9)	790 (0.8)	210 (11.3)
Ethnicity (%)						
White	69,234 (80.9)	575 (67.3)	14,035 (75.9)	10,205 (75.1)	59,683 (62.7)	1,585 (85.1)
Unknown	10,923 (12.8)	161 (18.9)	3,340 (18.0)	3,086 (22.7)	11,573 (12.2)	*
South Asian	2,007 (2.3)	38 (4.4)	369 (2.0)	65 (0.5)	13,883 (14.6)	123 (6.6)
Other	1,432 (1.7)	37 (4.3)	347 (1.9)	96 (0.7)	3,011 (3.2)	46 (2.5)
Black	1,016 (1.2)	29 (3.4)	238 (1.3)	72 (0.5)	4,669 (4.9)	76 (4.1)
Mixed	792 (0.9)	13 (1.5)	126 (0.7)	53 (0.4)	1,944 (2.0)	27 (1.4)
Chinese	203 (0.2)	*	44 (0.2)	15 (0.1)	473 (0.5)	*
Age (%)						
18-39	21,027 (24.6)	90 (10.5)	3,030 (16.4)	2,558 (18.8)	29,652 (31.1)	389 (21.3)
40-64	25,724 (30.0)	295 (34.5)	5,545 (30)	3,354 (24.7)	32,031 (33.6)	619 (33.8)
$>$65	38,856 (45.4)	470 (55.0)	9,924 (53.6)	7,680 (56.5)	33,553 (35.2)	822 (44.9)

$*$ We merged cases from these subgroups into the "Other" cohort for statistical disclosure controls. Age groups (18-39, 40-64, $>$65) are reported for adult patients with available age information. Patients younger than 18 years in the BH cohort (n = 33) were excluded from all analyses.

Table R17: Ethnicity-age intersectional algorithmic unfairness of unlearned MLP models of various machine unlearning methods on the CURIAL dataset.

	EO-TP ↓ (SD)				EO-FP ↓ (SD)				DP ↓ (SD)			
Forg. Ratio	0%	1%	5%	10%	0%	1%	5%	10%	0%	1%	5%	10%
OUH (wave 2)												
Original	0.131	-	-	-	0.062	-	-	-	0.121	-	-	-
Retrained	-	0.144(0.002)	0.146(0.005)	0.142(0.006)	-	0.060(0.006)	0.060(0.004)	0.065(0.004)	-	0.119(0.006)	0.120(0.003)	0.122(0.003)
Unlearning Methods												
FU (Ours)	-	**0.131**(0.001)	**0.127**(0.001)	**0.113**(0.002)	-	**0.062**(0.000)	**0.060**(0.000)	**0.055**(0.003)	-	**0.119**(0.000)	0.116(0.001)	**0.107**(0.001)
GA	-	0.143(0.010)	0.157(0.005)	0.152(0.012)	-	0.068(0.009)	0.136(0.014)	0.152(0.052)	-	0.126(0.006)	0.171(0.053)	0.165(0.038)
CR	-	0.140(0.007)	0.145(0.028)	0.158(0.021)	-	0.067(0.005)	0.082(0.024)	0.063(0.007)	-	0.123(0.004)	0.123(0.013)	0.109(0.017)
CF	-	0.144(0.008)	0.159(0.052)	0.147(0.005)	-	0.074(0.017)	0.115(0.065)	0.080(0.035)	-	0.129(0.011)	0.175(0.059)	0.165(0.006)
SCRUB	-	0.131(0.003)	0.146(0.008)	0.162(0.013)	-	0.063(0.003)	0.063(0.010)	0.069(0.010)	-	0.122(0.003)	**0.115**(0.003)	0.109(0.006)
ORTHO	-	0.149(0.019)	0.153(0.024)	0.153(0.020)	-	0.074(0.010)	0.096(0.028)	0.077(0.015)	-	0.124(0.010)	0.135(0.026)	0.137(0.022)
PUH (wave 2)												
Original	0.235	-	-	-	0.171	-	-	-	0.185	-	-	-
Retrained	-	0.201(0.046)	0.203(0.045)	0.187(0.043)	-	0.145(0.012)	0.142(0.018)	0.138(0.006)	-	0.167(0.010)	0.166(0.012)	0.157(0.010)
Unlearning Methods												
FU (Ours)	-	**0.168**(0.037)	**0.179**(0.017)	**0.165**(0.011)	-	**0.168**(0.001)	**0.156**(0.001)	**0.142**(0.000)	-	0.180(0.002)	**0.176**(0.003)	0.170(0.002)
GA	-	0.214(0.047)	0.226(0.043)	0.230(0.031)	-	0.174(0.013)	0.156(0.019)	0.157(0.037)	-	0.181(0.008)	0.176(0.014)	0.176(0.030)
CR	-	0.220(0.042)	0.215(0.038)	0.246(0.022)	-	0.169(0.010)	0.161(0.013)	0.167(0.013)	-	**0.179**(0.005)	0.179(0.011)	0.170(0.011)
CF	-	0.240(0.021)	0.209(0.010)	0.213(0.005)	-	0.171(0.011)	0.156(0.006)	0.171(0.025)	-	0.182(0.004)	0.179(0.011)	0.173(0.029)
SCRUB	-	0.219(0.033)	0.208(0.035)	0.230(0.042)	-	0.170(0.011)	0.162(0.012)	0.143(0.008)	-	0.180(0.006)	0.178(0.007)	**0.166**(0.011)
ORTHO	-	0.186(0.046)	0.218(0.036)	0.227(0.050)	-	0.168(0.004)	0.165(0.014)	0.168(0.043)	-	0.182(0.018)	0.176(0.006)	0.182(0.030)
UHB (wave 1)												
Original	0.174	-	-	-	0.044	-	-	-	0.046	-	-	-
Retrained	-	0.178(0.032)	0.188(0.034)	0.186(0.033)	-	0.049(0.006)	0.048(0.005)	0.046(0.005)	-	0.050(0.006)	0.049(0.005)	0.048(0.005)
Unlearning Methods												
FU (Ours)	-	0.230(0.000)	**0.239**(0.001)	**0.229**(0.002)	-	**0.045**(0.000)	0.064(0.002)	**0.049**(0.002)	-	**0.045**(0.000)	0.065(0.001)	**0.050**(0.001)
GA	-	0.230(0.013)	0.241(0.003)	0.236(0.010)	-	0.054(0.007)	0.095(0.024)	0.091(0.017)	-	0.055(0.008)	0.096(0.024)	0.091(0.016)
CR	-	0.236(0.042)	0.241(0.031)	0.238(0.023)	-	0.051(0.006)	**0.060**(0.015)	0.049(0.002)	-	0.052(0.006)	**0.060**(0.014)	0.051(0.003)
CF	-	0.233(0.019)	0.240(0.022)	0.237(0.031)	-	0.049(0.011)	0.099(0.013)	0.120(0.001)	-	0.051(0.011)	0.100(0.014)	0.121(0.001)
SCRUB	-	**0.224**(0.022)	0.242(0.018)	0.231(0.014)	-	0.046(0.004)	0.065(0.003)	0.050(0.006)	-	0.046(0.002)	0.065(0.003)	0.053(0.005)
ORTHO	-	0.236(0.025)	0.249(0.022)	0.231(0.021)	-	0.050(0.016)	0.068(0.025)	0.072(0.029)	-	0.051(0.016)	0.068(0.025)	0.073(0.029)
BH (wave 2)												
Original	0.269	-	-	-	0.154	-	-	-	0.238	-	-	-
Retrained	-	0.272(0.005)	0.292(0.028)	0.271(0.006)	-	**0.153**(0.004)	0.142(0.029)	0.124(0.026)	-	0.236(0.002)	0.233(0.007)	0.226(0.010)
Unlearning Methods												
FU (Ours)	-	**0.269**(0.000)	**0.241**(0.049)	**0.231**(0.013)	-	**0.153**(0.000)	0.168(0.000)	**0.159**(0.007)	-	**0.237**(0.000)	**0.231**(0.006)	**0.227**(0.012)
GA	-	0.277(0.032)	0.260(0.006)	0.279(0.028)	-	0.155(0.015)	0.167(0.015)	0.178(0.017)	-	0.250(0.039)	0.231(0.003)	0.239(0.014)
CR	-	0.288(0.022)	0.269(0.038)	0.273(0.039)	-	0.155(0.004)	**0.157**(0.041)	0.166(0.016)	-	0.242(0.005)	0.234(0.020)	0.228(0.013)
CF	-	0.269(0.004)	0.276(0.030)	0.256(0.000)	-	0.159(0.008)	0.170(0.020)	0.165(0.010)	-	0.240(0.007)	0.237(0.016)	0.234(0.017)
SCRUB	-	0.271(0.003)	0.278(0.026)	0.274(0.068)	-	0.155(0.005)	0.166(0.008)	0.161(0.027)	-	0.239(0.007)	0.235(0.012)	0.228(0.013)
ORTHO	-	0.269(0.006)	0.275(0.031)	0.260(0.072)	-	0.156(0.004)	0.168(0.013)	0.159(0.013)	-	0.237(0.010)	0.235(0.007)	0.229(0.015)

Table R18: Diagnostic performance (AUROC) and unlearning effectiveness (MIA-AUROC and MI-KnnDist) of various methods on the CURIAL for ethnicity-age intersectional unfairness evaluations. Note that MIA-AUROC and MI-KnnDist are evaluated by privacy attacks on the forgetting sets from the training data.

Prospective evaluation on OUH

	AUROC ↑ (SD) (OUH wave 2)				MIA-AUROC →0.5 (SD)			MI-KnnDist ↑ (SD)		
Forg. Ratio	0%	1%	5%	10%	1%	5%	10%	1%	5%	10%
Original	0.878	-	-	-	-	-	-	-	-	-
Retrained	-	0.875(0.001)	0.875(0.001)	0.874(0.002)	0.510(0.006)	0.473(0.005)	0.501(0.007)	4.05(0.313)	4.76(1.278)	4.22(0.428)
Unlearning Methods										
FU (Ours)	-	**0.876**(0.000)	**0.857**(0.001)	**0.851**(0.001)	0.746(0.019)	**0.629**(0.010)	**0.492**(0.022)	**3.36**(0.437)	**3.90**(0.617)	**3.93**(0.408)
GA	-	0.870(0.004)	0.833(0.015)	0.787(0.027)	0.799(0.018)	0.706(0.017)	0.524(0.031)	3.19(0.331)	3.45(0.281)	3.68(0.200)
CR	-	0.872(0.002)	0.822(0.054)	0.814(0.012)	0.767(0.013)	0.682(0.008)	0.457(0.035)	3.10(0.258)	3.51(0.331)	3.57(0.190)
CF	-	0.872(0.004)	0.794(0.014)	0.781(0.003)	0.744(0.011)	0.701(0.012)	0.489(0.039)	3.35(0.275)	3.24(0.332)	3.28(0.132)
SCRUB	-	0.874(0.002)	0.851(0.002)	0.831(0.005)	0.778(0.003)	0.748(0.003)	0.461(0.032)	3.16(0.279)	3.31(0.289)	3.86(0.429)
ORTHO	-	0.867(0.007)	0.849(0.006)	0.822(0.013)	**0.731**(0.009)	0.657(0.009)	0.539(0.012)	3.31(0.369)	3.40(0.123)	3.53(0.160)

External evaluations

	AUROC ↑ (SD) (PUH wave 2)				AUROC ↑ (SD) (UHB wave 1)				AUROC ↑ (SD) (BH wave 2)			
Forg. Ratio	0%	1%	5%	10%	0%	1%	5%	10%	0%	1%	5%	10%
Original	0.858	-	-	-	0.886	-	-	-	0.891	-	-	-
Retrained	-	0.859 (0.003)	0.856 (0.003)	0.851 (0.004)	-	0.884 (0.002)	0.884 (0.002)	0.883 (0.002)	-	0.893 (0.002)	0.890 (0.003)	0.890 (0.003)
Unlearning Methods												
FU (Ours)	-	0.853 (0.000)	**0.832** (0.001)	**0.828** (0.001)	-	**0.883** (0.000)	**0.866** (0.002)	**0.868** (0.001)	-	**0.890** (0.000)	**0.870** (0.001)	0.861 (0.001)
GA	-	0.852 (0.009)	0.816 (0.020)	0.772 (0.028)	-	0.876 (0.010)	0.852 (0.025)	0.804 (0.042)	-	0.885 (0.005)	0.856 (0.018)	0.816 (0.013)
CR	-	0.851 (0.004)	0.789 (0.076)	0.779 (0.015)	-	0.882 (0.003)	0.824 (0.064)	0.818 (0.024)	-	0.886 (0.003)	0.839 (0.046)	0.829 (0.020)
CF	-	0.854 (0.003)	0.774 (0.013)	0.768 (0.000)	-	0.881 (0.007)	0.794 (0.020)	0.803 (0.017)	-	0.880 (0.010)	0.835 (0.023)	0.827 (0.001)
SCRUB	-	**0.857** (0.002)	0.832 (0.005)	0.827 (0.010)	-	0.881 (0.002)	0.854 (0.009)	0.848 (0.011)	-	0.887 (0.002)	0.867 (0.011)	0.842 (0.013)
ORTHO	-	0.847 (0.016)	0.823 (0.023)	0.791 (0.042)	-	0.877 (0.009)	0.860 (0.012)	0.846 (0.029)	-	0.886 (0.004)	0.868 (0.010)	**0.864** (0.010)

Clinical thresholds: We agree that a practical guide on the threshold for acceptable performance drops can be necessary for clinical deployment. To address this issue, we first thoroughly investigate the performance of a retrained model and an unlearned model by our FU across a broader range of forgetting ratios. Specifically, on the MIMIC-IV dataset, we remove patient records from 1% to 25% forgetting ratios with a 1% increment, and plot the performance of FU and retraining at five forgetting ratios: 1%, 5%, 10%, 15%, 20% and 25% in Figure R5. As shown in the figure, FU exhibits a progressive decline in model utility, with AUROC dropping from 0.85 to 0.73, thereby widening the performance gap relative to the retrained baseline, which shows slightly lower AUROCs. FU's fairness metrics, such as EO-TP and EO-FP, generally exhibit superior performance over retraining across various forgetting ratios. Meanwhile, MIA-AUROC gradually approaches the retraining level (close to 0.5) as the forgetting ratio increases, illustrating more thorough forgetting and better privacy protections.

Those observations imply that model utility can be a more crucial metric for determining whether a full retraining is necessary, depending on the clinical scenarios encountered upon deployment. For example, a tiered monitoring system can be adopted on the predictive performance of the unlearning model with the following rules: a 3-5% AUROC drop will trigger a yellow warning flag indicating more

intensive monitoring, a 5-10% AUROC drop will incur a red flag for re-investigating the necessity of retraining, and a drop exceeding 10% will launch a retraining to be conducted immediately. Additional flagging systems for fairness or privacy protection metrics can be developed with similar criteria for performance drop and combined to support more comprehensive, real-time monitoring. However, it is worth noting that those criteria are set following empirical rules, and the complexity and challenges of clinical deployments will require those thresholds to be adjusted accordingly and dynamically.

We have correspondingly revised Section "Discussion" in the manuscript (lines 516-529, p. 23) and added Section "Determination of clinical thresholds" in Supplementary Note 3 (pp. 22).

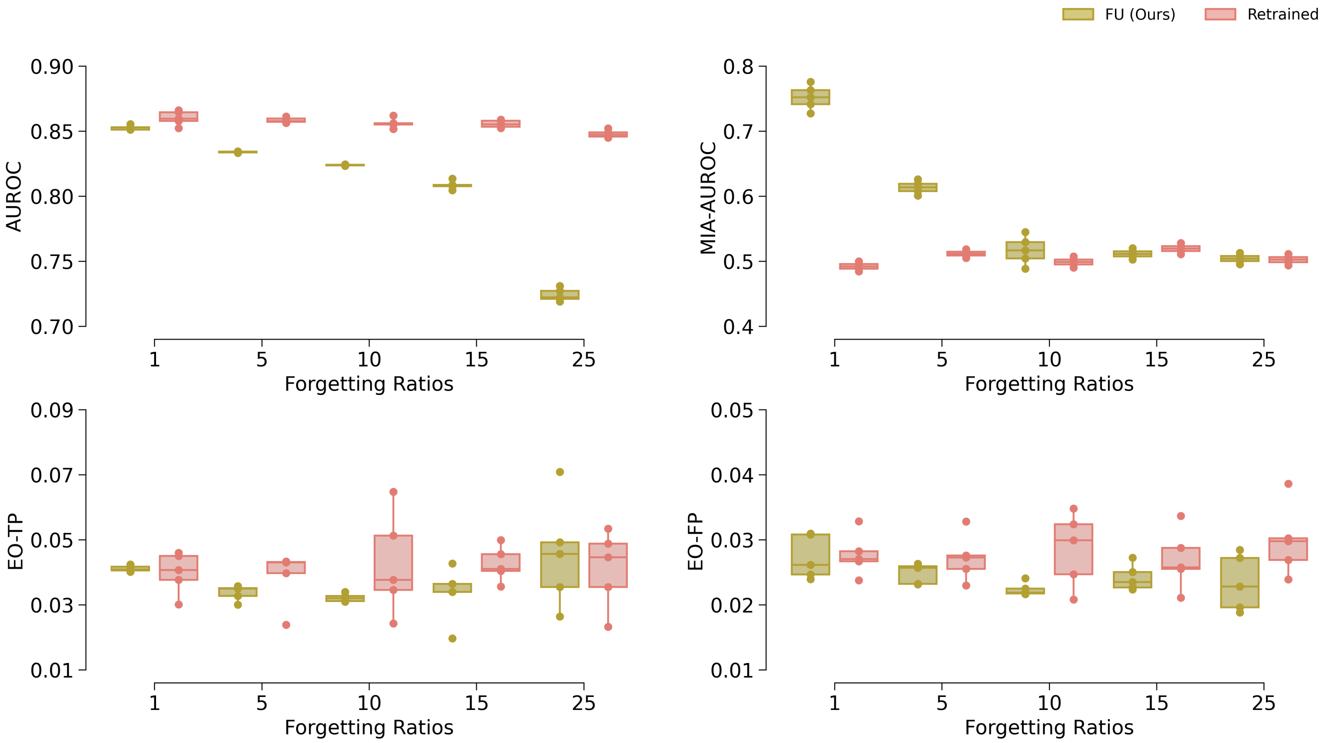

Figure R5: Performance comparisons of unlearned models by our FU and the retrained models across forgetting ratios from 1% to 25%, under the incremental forgetting protocol of consecutive 1% increments, on the mortality prediction task of MIMIC-IV.

Comment 4. *2. consider how fairness metrics and unlearning effectiveness could be communicated in real time to clinicians (for example, via dashboards or alerts or even some sort of system's ethics-panel;*

Reply 4. There can be several possible options for communicating the status of model fairness and unlearning effectiveness to clinicians, depending on the budget, available hardware, specific requirements, etc. For instance, with limited budgets, the status can be displayed as simple alert information through terminals or logs each time an unlearning operation is completed, e.g., "Warning: recent unlearning has caused ethnicity-related fairness to be reduced below the threshold, retraining is required". A more sophisticated, detailed dashboard can be developed, if necessary, to provide real-time monitoring of fairness metrics, unlearning effectiveness, diagnostic performance, and other relevant information, organised into tables, graphs, indicators, and related visualisations. This dashboard can be displayed on required devices, such as the central monitors of a care unit and wearable devices for doctors. Conversational interfaces can also be considered, in which clinicians can directly

consult the overall situations and seek advice from chatbots, e.g., whether unlearning is effective, or whether retraining should be done. Other IT communication tools, such as emails and Messages, can be used to deliver summary and alerting information to clinicians when necessary. Overall, we believe that real-time communication of unlearning performance could benefit clinicians in making better clinical decisions, while the method for conveying status should be determined by specific circumstances.

We have revised Section "Discussion" in the manuscript (lines 530-541, p. 23) to reflect this discussion.

Comment 5. *Finally, while the approach is tested on neural networks typical of today's clinical AI, some discussion of scaling the method to larger/complex architectures (such as transformers or multimodal models) would be relevant as a helpful perspective. That could help readers to understand what's is coming or even wide derivations of this research topic.*

Reply 5. To demonstrate the applicability of our approach to larger or more complex architectures, we first conduct additional evaluations on a 2-layer Transformer model[9] with 2.27M learnable parameters (detailed architecture outlined in Supplementary Table 5), in addition to the LSTM network with 20K learnable parameters on the eICU dataset for the mortality and shock prediction task. Tables R19 and R20 compare the performance of those two networks for the mortality and shock prediction tasks on the eICU dataset, respectively. As shown in the tables, FU consistently outperforms baselines across algorithmic fairness (EO-TP, EO-FP, and DP), model utility (AUROC), and unlearning effectiveness (MIA-AUROC and MI-KnnDist), for both the LSTM (20K parameters) and Transformer (2.27M) architectures across two clinical tasks. Those results demonstrate that, although we mainly adopt DNN architectures typical of today's clinical AI, such as LSTMs and MLPs, our FU method could also generalise to a more complex and larger DNN architecture, Transformer, which is the core architecture of modern large language models (LLMs) or foundation models.

The application of machine unlearning methods to medical foundation models with billion-scale parameters, though beyond the scope of this research, is a highly potential direction that deserves further discussion. As medical foundation models are usually trained on large-scale clinical records, the ability to rapidly remove specific EHRs from them to satisfy RtbF requirements can be crucial, as retraining a foundational model is impractical given the enormous computational resources required. How to achieve effective EHR removal in a medical foundation model without significantly impairing its utility, and, more importantly, how to maintain or improve the algorithmic fairness of the unlearned foundation model, can be interesting future investigations with significant scientific and clinical values.

We have accordingly revised Section "Discussion" in the manuscript (lines 578-588, pp. 24-25) and added Section "Scalability to more complex DNNs" in Supplementary Note 3 (p. 15).

Table R19: Performance comparisons of unlearning methods between an LSTM of 20K parameters and a Transformer with 2.27M parameters on the eICU dataset (mortality prediction) across the 1%, 5%, and 10% forgetting ratios.

	EO-TP $\downarrow$ (SD)				EO-FP $\downarrow$ (SD)				DP $\downarrow$ (SD)			
Forg. Ratio	0%	1%	5%	10%	0%	1%	5%	10%	0%	1%	5%	10%
**LSTM**												
Original	0.041	-	-	-	0.014	-	-	-	0.021	-	-	-
FU (Ours)	-	**0.032**(0.001)	**0.039**(0.002)	**0.036**(0.001)	-	**0.013**(0.001)	0.018(0.001)	**0.013**(0.001)	-	**0.019**(0.001)	**0.020**(0.000)	**0.019**(0.001)
GA	-	0.043(0.006)	0.048(0.010)	0.057(0.003)	-	0.018(0.002)	0.019(0.002)	0.026(0.003)	-	0.020(0.004)	0.037(0.005)	0.030(0.003)
CR	-	0.044(0.008)	0.077(0.023)	0.068(0.024)	-	0.013(0.003)	0.024(0.005)	0.028(0.010)	-	0.022(0.006)	0.036(0.021)	0.034(0.009)
CF	-	0.048(0.004)	0.070(0.005)	0.078(0.014)	-	0.014(0.003)	0.020(0.010)	0.019(0.003)	-	0.024(0.006)	0.024(0.004)	0.031(0.006)
SCRUB	-	0.047(0.009)	0.075(0.014)	0.075(0.019)	-	0.019(0.008)	0.023(0.004)	0.020(0.005)	-	0.023(0.008)	0.027(0.007)	0.027(0.004)
ORTHO	-	0.047(0.014)	0.068(0.006)	0.075(0.023)	-	0.017(0.002)	**0.017**(0.006)	0.022(0.003)	-	0.022(0.002)	0.021(0.004)	0.026(0.004)
**Transformer**												
Original	0.051	-	-	-	0.018	-	-	-	0.014	-	-	-
FU (Ours)	-	**0.048**(0.002)	**0.042**(0.001)	**0.039**(0.004)	-	**0.016**(0.002)	**0.014**(0.002)	**0.013**(0.001)	-	**0.012**(0.001)	**0.013**(0.002)	**0.012**(0.001)
GA	-	0.055(0.009)	0.055(0.004)	0.089(0.009)	-	0.024(0.005)	0.044(0.004)	0.047(0.002)	-	0.023(0.004)	0.040(0.004)	0.048(0.003)
CR	-	0.048(0.008)	0.060(0.030)	0.072(0.019)	-	0.024(0.004)	0.024(0.003)	0.023(0.006)	-	0.023(0.005)	0.024(0.002)	0.022(0.005)
CF	-	0.059(0.020)	0.066(0.020)	0.085(0.016)	-	0.024(0.006)	0.045(0.002)	0.043(0.003)	-	0.024(0.003)	0.040(0.002)	0.045(0.002)
SCRUB	-	0.050(0.014)	0.071(0.010)	0.103(0.009)	-	0.019(0.005)	0.019(0.008)	0.016(0.006)	-	0.019(0.006)	0.025(0.006)	0.024(0.008)
ORTHO	-	0.053(0.015)	0.049(0.028)	0.051(0.016)	-	0.027(0.004)	0.026(0.008)	0.031(0.010)	-	0.028(0.007)	0.027(0.007)	0.029(0.009)

	AUROC $\uparrow$ (SD)				MIA-AUROC $\rightarrow$0.5 (SD)			MI-KnnDist $\uparrow$ (SD)		
For. Ratio	0%	1%	5%	10%	1%	5%	10%	1%	5%	10%
**LSTM**										
Original	0.853	-	-	-	-	-	-	-	-	-
FU (Ours)	-	0.844(0.001)	**0.829**(0.002)	**0.811**(0.001)	0.617(0.017)	**0.506**(0.012)	0.514(0.011)	**17.97**(0.605)	**19.02**(0.453)	**18.68**(0.710)
GA	-	0.814(0.004)	0.773(0.022)	0.747(0.007)	0.702(0.022)	0.565(0.012)	0.463(0.032)	12.62(0.930)	15.40(0.410)	16.77(0.898)
CR	-	0.833(0.004)	0.805(0.009)	0.791(0.021)	0.665(0.009)	0.572(0.007)	0.515(0.040)	12.41(0.765)	18.22(0.461)	18.22(0.416)
CF	-	**0.848**(0.003)	0.826(0.003)	0.796(0.019)	**0.617**(0.013)	0.640(0.012)	0.484(0.038)	13.13(1.042)	14.37(0.856)	17.21(0.536)
SCRUB	-	0.840(0.005)	0.808(0.008)	0.784(0.003)	0.744(0.003)	0.709(0.003)	0.531(0.032)	12.42(0.795)	16.06(1.033)	18.62(0.510)
ORTHO	-	0.845(0.003)	0.816(0.004)	0.780(0.008)	0.665(0.009)	0.617(0.009)	**0.506**(0.012)	15.35(0.866)	16.02(0.604)	16.91(0.395)
**Transformer**										
Original	0.874	-	-	-	-	-	-	-	-	-
FU (Ours)	-	0.852(0.001)	0.835(0.002)	**0.813**(0.003)	**0.768**(0.013)	0.696(0.003)	**0.574**(0.004)	18.15(0.301)	**20.93**(0.705)	**19.63**(0.452)
GA	-	0.851(0.005)	0.813(0.006)	0.785(0.010)	0.807(0.013)	0.702(0.009)	0.599(0.006)	**18.19**(0.241)	15.99(0.463)	16.68(0.559)
CR	-	0.847(0.012)	**0.838**(0.011)	0.795(0.025)	0.819(0.011)	0.715(0.029)	0.582(0.020)	12.62(0.910)	18.18(0.565)	17.71(0.646)
CF	-	0.847(0.005)	0.813(0.001)	0.790(0.006)	0.786(0.022)	0.712(0.012)	0.604(0.011)	12.74(0.896)	13.77(0.623)	15.26(0.657)
SCRUB	-	**0.854**(0.004)	0.827(0.007)	0.797(0.006)	0.794(0.027)	**0.691**(0.012)	0.617(0.014)	14.33(0.354)	18.42(0.558)	18.19(0.721)
ORTHO	-	0.840(0.005)	0.829(0.012)	0.796(0.024)	0.797(0.028)	0.704(0.014)	0.601(0.019)	12.40(0.931)	18.94(0.615)	14.67(0.875)

Table R20: Performance comparisons of unlearning methods between an LSTM of 20K parameters and a Transformer of 2.27M parameters on the eICU dataset (shock prediction) across the 1%, 5%, and 10% forgetting ratios.

	EO-TP ↓ (SD)				EO-FP ↓ (SD)				DP ↓ (SD)			
Forg. Ratio	0%	1%	5%	10%	0%	1%	5%	10%	0%	1%	5%	10%
LSTM												
Original	0.070	-	-	-	0.045	-	-	-	0.052	-	-	-
FU (Ours)	-	**0.058**(0.001)	**0.054**(0.008)	**0.048**(0.001)	-	**0.040**(0.001)	**0.040**(0.003)	**0.035**(0.001)	-	**0.044**(0.001)	**0.038**(0.001)	**0.027**(0.001)
GA	-	0.083(0.008)	0.083(0.003)	0.097(0.020)	-	0.044(0.003)	0.050(0.005)	0.076(0.009)	-	0.047(0.010)	0.047(0.011)	0.077(0.014)
CR	-	0.084(0.004)	0.087(0.010)	0.088(0.009)	-	0.050(0.008)	0.062(0.007)	0.056(0.005)	-	0.079(0.011)	0.062(0.012)	0.059(0.009)
CF	-	0.064(0.016)	0.091(0.012)	0.093(0.007)	-	0.045(0.010)	0.056(0.019)	0.056(0.006)	-	0.055(0.015)	0.048(0.007)	0.044(0.004)
SCRUB	-	0.083(0.016)	0.078(0.013)	0.089(0.017)	-	0.049(0.010)	0.056(0.008)	0.061(0.017)	-	0.045(0.007)	0.049(0.006)	0.044(0.012)
ORTHO	-	0.075(0.011)	0.077(0.009)	0.094(0.037)	-	0.051(0.004)	0.046(0.005)	0.037(0.008)	-	0.053(0.004)	0.049(0.004)	0.041(0.006)
Transformer												
Original	0.064	-	-	-	0.038	-	-	-	0.042	-	-	-
FU (Ours)	-	0.060(0.000)	**0.052**(0.001)	**0.049**(0.001)	-	0.036(0.007)	**0.034**(0.001)	**0.031**(0.001)	-	**0.031**(0.000)	**0.030**(0.001)	**0.030**(0.005)
GA	-	0.064(0.005)	0.062(0.003)	0.096(0.013)	-	0.046(0.014)	0.051(0.007)	0.072(0.022)	-	0.048(0.012)	0.046(0.008)	0.066(0.018)
CR	-	0.092(0.026)	0.107(0.045)	0.081(0.013)	-	**0.035**(0.009)	**0.034**(0.004)	0.037(0.009)	-	0.032(0.007)	0.037(0.005)	0.037(0.012)
CF	-	**0.052**(0.022)	0.067(0.008)	0.068(0.005)	-	0.036(0.007)	0.048(0.006)	0.049(0.012)	-	0.039(0.008)	0.046(0.005)	0.046(0.010)
SCRUB	-	0.085(0.020)	0.095(0.024)	0.081(0.016)	-	0.050(0.008)	0.050(0.010)	0.054(0.008)	-	0.050(0.008)	0.057(0.009)	0.061(0.007)

	AUROC ↑ (SD)				MIA-AUROC →0.5 (SD)			MI-KnnDist ↑ (SD)		
Forgetting Ratio	0%	1%	5%	10%	1%	5%	10%	1%	5%	10%
LSTM										
Original	0.862	-	-	-	-	-	-	-	-	-
FU (Ours)	-	**0.853**(0.000)	**0.841**(0.000)	**0.801**(0.000)	**0.585**(0.019)	**0.492**(0.010)	**0.509**(0.022)	**5.61**(0.176)	**5.62**(0.125)	**5.66**(0.109)
GA	-	0.845(0.002)	0.824(0.029)	0.749(0.038)	0.639(0.018)	0.544(0.017)	0.511(0.031)	4.31(0.350)	5.23(0.188)	5.37(0.085)
CR	-	0.852(0.002)	0.824(0.025)	0.788(0.036)	0.598(0.013)	0.525(0.008)	0.517(0.035)	4.58(0.301)	5.28(0.249)	5.19(0.149)
CF	-	0.846(0.006)	0.822(0.002)	0.790(0.008)	0.614(0.011)	0.642(0.012)	0.484(0.039)	5.50(0.270)	5.29(0.300)	5.38(0.175)
SCRUB	-	0.842(0.002)	0.806(0.004)	0.773(0.010)	0.784(0.003)	0.680(0.003)	0.510(0.032)	4.81(0.102)	5.17(0.213)	5.23(0.262)
ORTHO	-	0.845(0.002)	0.826(0.003)	0.784(0.018)	0.632(0.009)	0.584(0.009)	0.512(0.012)	4.69(0.312)	5.04(0.153)	5.23(0.214)
Transformer										
Original	0.874	-	-	-	-	-	-	-	-	-
FU (Ours)	-	**0.859**(0.000)	**0.844**(0.001)	**0.831**(0.001)	**0.687**(0.015)	**0.608**(0.025)	0.511(0.009)	**5.76**(0.223)	**5.69**(0.300)	**5.76**(0.127)
GA	-	0.853(0.012)	0.834(0.005)	0.735(0.006)	0.706(0.032)	0.627(0.020)	0.545(0.016)	5.41(0.131)	4.48(0.190)	4.68(0.180)
CR	-	0.853(0.009)	0.828(0.009)	0.800(0.034)	0.716(0.013)	0.664(0.014)	0.570(0.012)	4.51(0.075)	5.05(0.105)	5.46(0.088)
CF	-	0.848(0.007)	0.811(0.001)	0.803(0.015)	0.737(0.026)	0.646(0.020)	**0.498**(0.017)	4.84(0.063)	5.50(0.120)	5.36(0.150)
SCRUB	-	0.849(0.011)	0.826(0.008)	0.806(0.006)	0.747(0.019)	0.674(0.022)	0.538(0.010)	5.00(0.155)	5.04(0.164)	5.59(0.265)
ORTHO	-	0.845(0.009)	0.823(0.008)	0.801(0.010)	0.725(0.014)	0.664(0.036)	0.524(0.012)	3.99(0.157)	5.49(0.131)	5.12(0.174)

Comment 6. *Remarks on code availability: Overall, the code as-is is hard to run "out of the box". Imports break because there's no clear package hierarchy, magic numbers (e.g. hard-coded input dims) and a missing forward() in the LSTM mean nothing actually executes, hyperparameters and seeding live in three different scripts, and there's no requirements file to tell you how to install dependencies or fetch the CURIAL/eICU data. You'd spend more time fixing broken imports, wiring up data loaders, and guessing which flags to pass than actually experimenting with unlearning methods. A simple reorganization into a proper models/, training/, and utils/ package, a unified config (or shared flags), correct docstrings, global seeding, plus a short README and pinned requirements.txt would make it usable and reproducible.*

Reply 6. We have thoroughly revised the codebase and fixed all the problems mentioned. In the revised release, we reorganise the repository into a clear package structure with a single entry point (`main.py`) and provide two options for installing dependencies, a script (`build_env.sh`) for ubuntu installation via `bash` or a pinned dependency file (`requirements.txt`) for general `pip` installation. All model components, including the LSTM, expose an explicit `forward` method. We consolidate all hyperparameters, including input dimensions and random seeds, into a single YAML configuration file passed to the main script (e.g., `python main.py --config_path config.yaml`), which can be easily modified as needed. We rewrite the README file to provide detailed instructions for reproducing the experimental results, including dependency installation, dataset fetching and preprocessing, explanations of key configurations, customisation of data loaders and configurations for new datasets, etc. Due to data protection laws and regulations, we are not permitted to share or distribute the datasets directly; however, the README includes detailed instructions for obtaining the raw data from the official sources and performing the required preprocessing steps to reproduce the experimental results. Our codebase can be downloaded from the following link: `https://drive.google.com/drive/folders/138JC7uSureRrTnXz7bOAqFJ1nz8W5r4X?usp=sharing`.

References

1. Johnson, A.E., Bulgarelli, L., Shen, L., Gayles, A., Shammout, A., Horng, S., Pollard, T.J., Hao, S., Moody, B., Gow, B. et al. (2023). Mimic-iv, a freely accessible electronic health record dataset. Scientific data *10*, 1.

2. Gupta, M., Gallamoza, B., Cutrona, N., Dhakal, P., Poulain, R., and Beheshti, R. (2022). An Extensive Data Processing Pipeline for MIMIC-IV. In Proceedings of the 2nd Machine Learning for Health symposium vol. 193 of *Proceedings of Machine Learning Research*. PMLR pp. 311–325. URL: `https://proceedings.mlr.press/v193/gupta22a.html`.

3. Madras, D., Creager, E., Pitassi, T., and Zemel, R. (2018). Learning adversarially fair and transferable representations. In International Conference on Machine Learning. PMLR pp. 3384–3393.

4. Fredrikson, M., Jha, S., and Ristenpart, T. (2015). Model inversion attacks that exploit confidence information and basic countermeasures. In Proceedings of the 22nd ACM SIGSAC conference on computer and communications security. pp. 1322–1333.

5. Zhou, Z., Zhu, J., Yu, F., Li, X., Peng, X., Liu, T., and Han, B. (2024). Model inversion attacks: A survey of approaches and countermeasures. arXiv preprint arXiv:2411.10023.

6. Shamsian, A., Shaar, E., Navon, A., Chechik, G., and Fetaya, E. (2025). Go beyond your means: Unlearning with per-sample gradient orthogonalization. arXiv preprint arXiv:2503.02312.

7. El Emam, K., and Arbuckle, L. (2013). Anonymizing Health Data: Case Studies and Methods to Get You Started. 1st ed.. Sebastopol: O'Reilly Media, Inc. ISBN 1449363075.

8. Abadi, M., Chu, A., Goodfellow, I., McMahan, H.B., Mironov, I., Talwar, K., and Zhang, L. (2016). Deep learning with differential privacy. In Proceedings of the 2016 ACM SIGSAC conference on computer and communications security. pp. 308–318.

9. Vaswani, A., Shazeer, N., Parmar, N., Uszkoreit, J., Jones, L., Gomez, A.N., Kaiser, Ł., and Polosukhin, I. (2017). Attention is all you need. Advances in neural information processing systems *30*.

10. Lin, S., Zhang, X., Susilo, W., Chen, X., and Liu, J. (2024). Gdr-gma: Machine unlearning via direction-rectified and magnitude-adjusted gradients. In Proceedings of the 32nd ACM International Conference on Multimedia. pp. 9087–9095.

11. Feng, Q., Tu, J., Kang, M., Zhao, H., Zhang, C., and Qian, H. (2025). Fg-oriu: Towards better forgetting via feature-gradient orthogonality for incremental unlearning. In Proceedings of the IEEE/CVF International Conference on Computer Vision. pp. 1957–1967.

12. Ateniese, G., Mancini, L.V., Spognardi, A., Villani, A., Vitali, D., and Felici, G. (2015). Hacking smart machines with smarter ones: How to extract meaningful data from machine learning classifiers. International Journal of Security and Networks *10*, 137–150.

13. Ganju, K., Wang, Q., Yang, W., Gunter, C.A., and Borisov, N. (2018). Property inference attacks on fully connected neural networks using permutation invariant representations. In Proceedings of the 2018 ACM SIGSAC conference on computer and communications security. pp. 619–633.

14. Tramèr, F., Zhang, F., Juels, A., Reiter, M.K., and Ristenpart, T. (2016). Stealing machine learning models via prediction {APIs}. In 25th USENIX security symposium (USENIX Security 16). pp. 601–618.

15. Wang, B., and Gong, N.Z. (2018). Stealing hyperparameters in machine learning. In 2018 IEEE symposium on security and privacy (SP). IEEE pp. 36–52.

16. Konečnỳ, J., McMahan, H.B., Yu, F.X., Richtárik, P., Suresh, A.T., and Bacon, D. (2016). Federated learning: Strategies for improving communication efficiency. arXiv preprint arXiv:1610.05492.

17. Xu, J., Glicksberg, B.S., Su, C., Walker, P., Bian, J., and Wang, F. (2021). Federated learning for healthcare informatics. Journal of healthcare informatics research *5*, 1–19.

18. Lundberg, S.M., and Lee, S.I. (2017). A unified approach to interpreting model predictions. Advances in neural information processing systems *30*.
